# A monomer–dimer switch modulates the activity of plant adenosine kinase

**DOI:** 10.1093/jxb/eraf094

**Published:** 2025-03-10

**Authors:** David Jaroslav Kopečný, Armelle Vigouroux, Jakub Bělíček, Martina Kopečná, Radka Končitíková, Jaroslava Friedecká, Václav Mik, Klára Supíková, Jan František Humplík, Marine Le Berre, Stephan Plancqueel, Miroslav Strnad, Klaus von Schwartzenberg, Ondřej Novák, Solange Moréra, David Kopečný

**Affiliations:** Department of Experimental Biology, Faculty of Science, Palacký University, Olomouc CZ-77900, Czech Republic; Université Paris-Saclay, CEA, CNRS, Institute for Integrative Biology of the Cell (I2BC), F-91198 Gif-sur-Yvette, France; Department of Experimental Biology, Faculty of Science, Palacký University, Olomouc CZ-77900, Czech Republic; Department of Experimental Biology, Faculty of Science, Palacký University, Olomouc CZ-77900, Czech Republic; Department of Experimental Biology, Faculty of Science, Palacký University, Olomouc CZ-77900, Czech Republic; Laboratory of Growth Regulators, Faculty of Science, Palacký University & Institute of Experimental Botany of the Czech Academy of Sciences, Olomouc CZ-77900, Czech Republic; Department of Experimental Biology, Faculty of Science, Palacký University, Olomouc CZ-77900, Czech Republic; Department of Experimental Biology, Faculty of Science, Palacký University, Olomouc CZ-77900, Czech Republic; Department of Chemical Biology, Faculty of Science, Palacký University, Olomouc CZ-77900, Czech Republic; Université Paris-Saclay, CEA, CNRS, Institute for Integrative Biology of the Cell (I2BC), F-91198 Gif-sur-Yvette, France; Université Paris-Saclay, CEA, CNRS, Institute for Integrative Biology of the Cell (I2BC), F-91198 Gif-sur-Yvette, France; Laboratory of Growth Regulators, Faculty of Science, Palacký University & Institute of Experimental Botany of the Czech Academy of Sciences, Olomouc CZ-77900, Czech Republic; Institute for Plant Science and Microbiology, Universität Hamburg, D-22609 Hamburg, Germany; Laboratory of Growth Regulators, Faculty of Science, Palacký University & Institute of Experimental Botany of the Czech Academy of Sciences, Olomouc CZ-77900, Czech Republic; Université Paris-Saclay, CEA, CNRS, Institute for Integrative Biology of the Cell (I2BC), F-91198 Gif-sur-Yvette, France; Department of Experimental Biology, Faculty of Science, Palacký University, Olomouc CZ-77900, Czech Republic; Cardiff University, UK

**Keywords:** Adenosine kinase, crystal structure, cytokinin, overexpression, *Physcomitrella patens*, purine, riboside, SnRK, *Zea mays*

## Abstract

Adenosine undergoes ATP-dependent phosphorylation catalyzed by adenosine kinase (ADK). In plants, ADK also phosphorylates cytokinin ribosides, transport forms of the hormone. Here, we investigated the substrate preferences, oligomeric states, and structures of ADKs from moss (*Physcomitrella patens*) and maize *(Zea mays*) alongside metabolomic and phenotypic analyses. We showed that dexamethasone-inducible *ZmADK* overexpressor lines in Arabidopsis can benefit from a higher number of lateral roots and larger root areas under nitrogen starvation. We discovered that maize and moss enzymes can form dimers upon increasing protein concentration, setting them apart from the monomeric human and protozoal ADKs. Structural and kinetic analyses revealed a catalytically inactive unique dimer. Within the dimer, both active sites are mutually blocked. The activity of moss ADKs, exhibiting a higher propensity to dimerize, was 10-fold lower compared with maize ADKs. Two monomeric structures in a ternary complex highlight the characteristic transition from an open to a closed state upon substrate binding. This suggests that the oligomeric state switch can modulate the activity of moss ADKs and probably other plant ADKs. Moreover, dimer association represents a novel negative feedback mechanism, helping to maintain steady levels of adenosine and AMP.

## Teaser

The switch from active monomers to inactive dimers in plant adenosine kinases impacts overall enzyme activity and represents a novel negative feedback loop mechanism to maintain steady levels of adenosine and AMP.

## Introduction

Cytokinins (*N*^6^-substituted adenine/adenosine derivatives) regulate cell division and many developmental events (Mok and Mok, 2001; Sakakibara, 2006). They are effective long-distance signaling molecules transported within the xylem and phloem. The xylem-transported cytokinin is predominantly *trans*-zeatin riboside (*t*ZR), while isopentenyl adenosine (iPR) and *cis*-zeatin riboside (*c*ZR) exist in the phloem. Mainly bases (iP, *t*Z, and *c*Z) bind to histidine kinase receptors and trigger the signaling cascade leading to primary hormone responses (Sakakibara, 2006). Cytokinin ribosides are thus considered long-distance transport forms of active hormone (Hirose *et al.*, 2008), and together with other nucleosides they are moved to the cytosol by members of the equilibrative nucleoside transporter (ENT) family (Wormit *et al.*, 2004; Girke *et al.*, 2014). Increased nucleoside import into the cytosol has been reported upon nitrogen starvation (Cornelius *et al.*, 2012; Melino *et al.*, 2018).

**Fig. 1.**
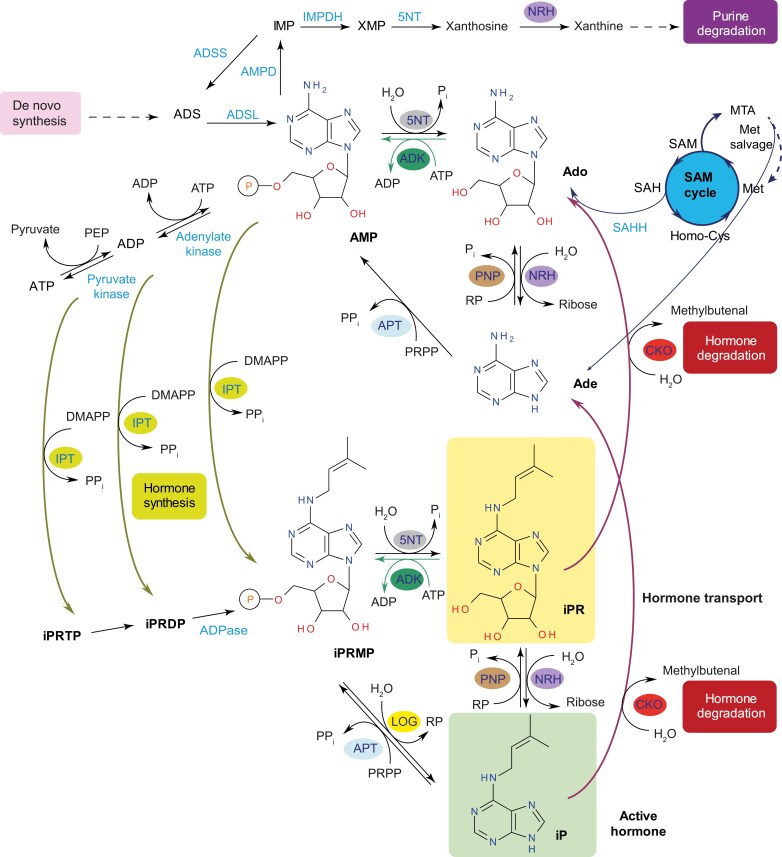
A scheme of adenosine and cytokinin riboside interconversion to their nucleobase and monophosphate derivatives. Interconversion between nucleoside monophosphates and nucleosides is catalyzed by adenosine kinase (ADK) and 5'-nucleotidase (5NT). Nucleoside N-ribohydrolase (NRH) and purine nucleoside phosphorylase (PNP) facilitate the conversion between bases and ribosides. Adenine phosphoribosyltransferases (APT) and cytokinin phosphoribohydrolase, known as LONELY GUY (LOG), catalyze the conversion between nucleobase and riboside monophosphate. Additional enzymes involved in the process include AMP dehydrogenase (AMPD), ADS lyase (ADSL), ADS synthetase (ADSS), cytokinin oxidase/dehydrogenase (CKO), IMP dehydrogenase (IMPDH), isopentenyl transferase (IPT), and SAH hydrolase (SAHH).

Nucleosides are hydrolyzed by nucleosidases (NRHs) to the corresponding bases and ribose (Jung *et al.*, 2009, 2011; Kopečná *et al.*, 2013; Baccolini and Witte, 2019). Purine nucleosides and bases are recycled to nucleoside monophosphates (a salvage pathway) to reduce the energy for *de novo* synthesis (Zrenner *et al.*, 2006; Ashihara *et al.*, 2018). An important role has been shown for adenosine (Ado) kinase (ADK; Moffatt *et al.*, 2000, 2002; Schoor *et al.*, 2011) and adenine phosphoribosyltransferase (APT) (Moffatt and Somerville, 1988; Allen *et al.*, 2002), which both produce AMP (Fig. 1). ADK regulates intracellular Ado pools and extracellular adenylate levels. It phosphorylates Ado-derived analogs at the 5'-hydroxyl group, including cytokinin ribosides, and uses ATP as a co-substrate as reported for isoforms from wheat, yellow lupin, moss, Arabidopsis, or tobacco (Chen and Eckert, 1977; Guranowski, 1979; von Schwartzenberg *et al.*, 1998; Moffatt *et al.*, 2000; Kwade *et al.*, 2005). Resulting cytokinin riboside monophosphates (iPRMP, *t*ZRMP, and *c*ZRMP) are hydrolyzed into an active hormone by the LONELY GUY phosphoribohydrolase (LOG) (Kuroha *et al.*, 2009).

Maintenance of AMP levels is essential for its further interconversion to ADP and ATP, energy-rich molecules critical for all metabolic pathways. AMP is also needed to maintain the purine nucleotide pools and catabolism. Although AMP is also *de novo* synthesized in plastids, *adk* and *apt* mutants exhibit a reduced or a complete loss of fertility, changes in transmethylation reactions, and abnormal cell walls, confirming their essential role in AMP production (Allen *et al.*, 2002; Schoor *et al.*, 2011). ADK-deficient plants have impaired root growth, and stamen and petal development, crinkled rosette leaves, reduced meristem size, and increased cytokinin content. The primary sources of Ado, apart from its import into cells and degradation of RNA in vacuoles, are *S*-adenosyl homocysteine (SAH) hydrolase (SAHH) and purine nucleoside phosphorylase (PNP) (Sauter *et al.*, 2013; Bromley *et al.*, 2014) that catalyzes the ribosylation reaction of adenine. ADK must steadily remove Ado to prevent the feedback inhibition of SAHH, which belongs to the *S*-adenosyl-l-methionine (SAM) cycle. Consequently, this maintains the rate of SAM-dependent transmethylation reactions (Moffatt *et al.*, 2002). Apart from the NRH reaction, the primary source of adenine is the hydrolysis of methyl-5'-thioAdo (MTA) in reactions leading from SAM to polyamines, ethylene, and nicotinamide (Sauter *et al.*, 2013). Finally, Ado and adenine appear as products of cytokinin degradation by cytokinin oxidase/dehydrogenase (CKO/CKX; reviewed in Schmülling *et al.*, 2003), while AMP, ADP, or ATP are used by isopentenyl transferase (IPT) for cytokinin biosynthesis (Fig. 1).

Ado is one of the most ancient signaling molecules. Ado and other purine and pyrimidine nucleotides trigger signaling through purinergic receptors. Four subtypes of P1 receptors bind Ado, whereas two subtypes of P2 receptors bind tri- and dinucleotides (Burnstock, 2007; Khakh and Burnstock, 2009). The first plant Ado receptor was discovered only 10 years ago (Choi *et al.*, 2014). Because plants lack adenine/Ado deaminase, only AMP is deaminated to IMP by AMP deaminase (Xu *et al.*, 2005; Han *et al.*, 2006). IMP then undergoes either oxidation to XMP and further to xanthine into the purine degradation pathway or is converted to adenylosuccinate (ADS) by ADS synthetase and further back to AMP by ADS lyase (Hatch, 1966) (Fig. 1). It is known that protozoan parasites such as *Toxoplasma gondii*, which cannot synthesize purines *de novo*, access nucleosides from their mammalian host mainly via ADK, and use them for DNA synthesis. (Schwartzman and Pfefferkorn, 1982).

There are several documented structures of ADK from *T. gondii* (Schumacher *et al.*, 2000; Zhang *et al.*, 2007), *Trypanosoma brucei* (Kuettel *et al*, 2011; Timm *et al.*, 2014), or humans (Mathews *et al.*, 1998; Muchmore *et al.*, 2006). In each case, the enzyme is composed of a large and a small domain. The large domain comprises the low-affinity binding co-substrate ATP site, and the groove between the large and small domains contains the high-affinity Ado-binding site. Upon substrate binding, the small domain moves from an open to a closed conformation and seals the cavity. Substrate derivatives, such as 2-deoxyriboside and arabinoside derivatives, are very poor substrates for rabbit ADK (Miller *et al.*, 1979a). Adenine arabinoside (AraA) is an efficient inhibitor of TgADK *in vivo* (Pfefferkorn and Pfefferkorn, 1976). However, there are more potent ligands, such as *N*^6^-(*p*-methoxybenzoyl)Ado or 7-iodo-7-deazaAdo (iodotubercidin) (Iltzsch *et al.*, 1995).

Here, we focused on the metabolism of nucleosides through the function of ADK from the seed plant model maize (*Zea mays*) and the early divergent model plant moss *Physcomitrella patens*, *in vitro* and *in planta*. Given the known adverse effects of lower ADK activity on plant growth (Allen *et al.*, 2002; Schoor *et al.*, 2011), we prepared homozygous dexamethasone-inducible *ZmADK* overexpressor lines (for all three maize *ADK* genes) in *Arabidopsis thaliana* to test the hypothesis that plants benefit from increased ADK activity upon stress conditions. Our findings demonstrate the benefits of increased ADK activity *in planta.* We show the crucial role of the monomer for ADK activity, and report, for the first time, the ability of plant ADKs to associate into an inactive dimer. The existence of the dimer was further confirmed by X-ray crystallography on ZmADK2, and the structure explains why this oligomeric state is not functional. Furthermore, structural analysis of two ternary complexes of ZmADK3 and PpADK1 together with kinetic and binding studies provide insight into nucleoside recognition and conformational changes of the enzyme.

## Materials and methods

### Cloning, expression, and purification of ADKs from *P. patens* and *Z. mays*

Total RNA was extracted from various maize (*Z. mays* cv. Cellux, Morseva) organs and *Physcomitrella* (‘Gransden 2004’ strain) at the protonema stage using the RNAqueous kit and plant RNA Isolation Aid. The cDNA was synthesized with Superscript III reverse transcriptase and oligo(dT) primers (www.thermofisher.com). The sequences encoding maize ADKs, specifically *ZmADK1* (Zm00001d051157, 1029 bp), *ZmADK2* (Zm00001d017271, 1029 bp), and *ZmADK3* (Zm00001d003017, 1035 bp), were cloned with Platinum™ SuperFi™ DNA Polymerase (www.thermofisher.com) into a pCDFDuet His-tag vector (Novagen, www.merckmillipore.com) using two forward primers containing a *Sac*I site and a common reverse primer with a *Kpn*I site (see Supplementary Table S1). Similarly, two sequences for moss ADKs were cloned, namely *PpADK1* (Pp3c3_10800, 1032 bp) and *PpADK2* (Pp3c13_10550, 1026 bp) using primer pairs containing *Sac*I/*Kpn*I and *Bam*HI/*Xho*I sites (Supplementary Table S1. The constructs were transformed into T7 Express *I*^*q*^*Escherichia coli* cells (www.neb.com).

Protein production was conducted at 200 rpm and 18 °C overnight with 0.5 mM isopropylthio-β-galactoside. The enzymes were subsequently purified using a Nickel-HiTrap IMAC FF column (https://www.cytivalifesciences.com) on an NGC Medium-Pressure Liquid Chromatography System (https://www.bio-rad.com) and further purified by gel filtration chromatography on a Superdex 200 10/30 HR column (www.gelifesciences.com) into 20 mM Tris–HCl buffer, pH 7.5, 100 mM NaCl with 1% glycerol. The yield after purification was ~4–6 mg per 100 ml of culture. Cells were analyzed for the expression of the His-tagged protein by NuPAGE (www.thermofisher.com) and using activity measurements. NuPAGE electrophoresis was performed in MES buffer on a 4–12% Bis-Tris gel. Protein content was quantified using the Coomassie Plus (Bradford) protein assay kit (www.thermofisher.com).

### Site-directed mutagenesis of *ZmADK3*

All *ZmADK3* mutants were cloned by 30 cycle PCRs using tail-to-tail oriented phosphorylated primers, with the mutation being located at the 5′ end of one of the primers. The following mutants were cloned using the primers given in Supplementary Table S1: D19A, R132A, N222A, E225A, N295A, and D299A. The products were treated with *Dpn*I restriction enzyme, gel purified, and ligated using T4 DNA ligase (www.thermofisher.com). Clones were transformed into T7 express *I*^*q*^*E. coli* competent cells.

### Synthesis of *N*^6^-methylAdo, *N*^6^,*N*^6^-dimethylAdo, *N*^6^-isopropylAdo, and *N*^6^-isobutylAdo

*N* ^6^-MethylAdo was prepared by mixing a suspension of 6-chloropurine riboside (1 g, 3.49 mmol) heated in 33% methylamine solution in absolute ethanol (4.33 ml, 34.9 mmol) at 90 °C for 4 h. *N*^6^,*N*^*6*^-DimethylAdo was prepared by mixing a suspension of 6-chloropurine riboside (1 g, 3.49 mmol) with dimethylamine hydrochloride (0.569 g, 6.98 mmol) and triethylamine (1.7 ml, 12.21 mmol), and heating in 20 ml of methanol at 100 °C for 4 h. *N*^6^-IsopropylAdo and *N*^6^*-*isobutylAdo were prepared in a one-step reaction by heating 6-chloropurine riboside with the corresponding amine in *n*-propanol at 85 °C, with triethylamine serving as an auxiliary base. A suspension of 6-chloropurine riboside (100 mg, 0.349 mmol), either isopropylamine (5 eq., 149 μl, 1.745 mmol) or isobutylamine (1.2 eq., 42 µl, 0.419 mmol), along with triethylamine (1.2 eq., 121 μl, 0.872 mmol), was heated in pressure tubes in 0.25 M *n-*propanol at 85 °C for 4 h.

The reaction mixtures were concentrated under reduced pressure and the product was purified by silica column chromatography using chloroform/methanol as a mobile phase, starting from 25:1 (v/v) with a methanol gradient. The progress of the reaction was monitored by TLC using Silica F254 aluminum plates (www.avantorsciences.com), and the spots were detected by 254 nm UV light and/or by vanillin staining. Chromatographic purity and mass of the prepared compounds were determined using the AQUITY UPLC H-Glass system (www.waters.com) on a Symmetry C18 column (150×2.1 mm, particle size 5 µm, www.waters.com). NMR spectra were recorded on an ECA-500 (www.jeol.com) operating at a frequency of 500 MHz (^1^H) and 125 MHz (^13^C). Substances were dissolved in DMSO-*d*_6_, and chemical shifts were calibrated to the residual/solvent peak DMSO-*d*_5_, 2.49 ppm for ^1^H and DMSO-*d*_6_, 39.5 ppm for ^13^C. High-resolution mass spectra were obtained on a 1290 Infinity II Autoscale preparative LC/MSD system (www.agilent.com) coupled simultaneously to a 6230 Time-of-Flight LC/MS mass spectrometer (LC/TOF, Agilent) with an electrospray ionization source Dual AJS ESI.

The final yield of *N*^6^*-*methylAdo [6-methylamino-9*H-*purine-9-β-d-riboside] was 942 mg (96%), that of *N*^6^,*N*^6^*-*dimethylAdo [6-(dimethylamino)-9*H-*purine-9-β-d-riboside] was 995 mg (97%), that of *N*^6^-isopropylAdo was 93 mg (86%), and that of *N*^6^-isobutylAdo was 80 mg (71%).

### Enzyme kinetics

Substrate analogs were purchased from Sigma-Aldrich (www.sigmaaldrich.com). The ADK activity was measured spectrophotometrically on an Agilent 8453 UV-Vis spectrophotometer (www.agilent.com) at 30 °C using a coupled reaction with pyruvate kinase (PK) and lactate dehydrogenase (LDH) (www.sigmaaldrich.com) (Blondin *et al.*, 1994). In this assay, PK converts ADP produced by ADK and phosphoenolpyruvate (PEP) to pyruvate. Subsequently, LDH then converts pyruvate to lactate using the coenzyme NADH, with the conversion monitored at 340 nm. The reaction was conducted in 50 mM Tris–HCl buffer at pH 7.5, containing 50 mM KCl, 6 mM MgCl_2_, 300 µM PEP, 200 µM NADH, 10 mM ATP, 2 U of PK, and 2.5 U of LDH, using various nucleoside substrates. Kinetic constants were determined using GraphPad Prism 8.0 software (www.graphpad.com). Each measurement was performed six times using repetitive purified enzymes. The activities of all five ADKs were also measured by HPLC (Moffatt *et al.*, 1991). The reaction mixture comprised 50 mM Tris–HCl pH 7.5, 5 mM MgCl_2_, 0.5 mM DTT, 30 mM NaF, 5 mM ATP, 40 µM substrate, and 2–150 ng of purified ADK, depending on the isoform. The reaction was incubated at 30 °C for up to 1 h. A conversion of Ado to AMP was analyzed in UV mode using a diode array detector after separation on a LiChrosphere 60 RP-Select B (5 µm) LiChroCART column 250-4 (www.merckmillipore.com) equilibrated with 10 mM ammonium formate at pH 3.7 and gradually eluted with 100% acetonitrile. Similarly, the conversion of cytokinin ribosides to monophosphates was followed on a LiChrosphere 60 RP-Select B (5 µm) LiChroCART column 125-4 column equilibrated with 10 mM triethylamine at pH 5.4 plus 10% methanol, and stepwise eluted with 100% methanol.

### Affinity, thermal stability, and small-angle X-ray scattering, analytical ultracentrifugation, and dynamic light scattering measurements

The microscale thermophoresis (MST) method was used to determine the binding affinity of various ribosides for ZmADK2 and PpADK1. Proteins were fluorescently labeled with RED-tris-NTA dye (www.nanotemper-technologies.com) using a 1:1 dye/protein molar ratio. The labeled protein was adjusted to 100–300 nM in 50 mM HEPES buffer pH 7.5, 1 mM MgCl_2_, and 0.2% Tween. Measurements were performed in premium capillaries on a Monolith NT.115 instrument at 30 °C with 5 s/30 s/5 s laser off/on/off times, respectively, with continuous sample fluorescence recording. Thermal stability was measured by nano-differential scanning fluorimetry on a Prometheus NT.48 instrument (www.nanotemper-technologies.com) in various buffers covering a pH (7.0–9.5) and temperature range (25–95 °C), with a heating rate of 1 °C min^–1^, and using NT melting control software. Protein unfolding was measured by detecting the temperature-induced change in tryptophan fluorescence intensity at emission wavelengths of 330±5 nm and 350±5 nm. The melting temperature (*T*_m_) was deduced from the maximum of the first derivative of the fluorescence ratios F_350_/F_330_.

Small-angle X-ray scattering (SAXS) data were measured on a SWING beamline at the SOLEIL synchrotron (www.synchrotron-soleil.fr) using an EigerX-4M detector. Exposure time was 500 ms, detector distance was 1790 mm, and X-ray wavelength was 1.033 Å. Purified proteins were analyzed in 50 mM Tris–HCl, pH 7.5 at three concentrations up to 5 mg ml^–1^. Data were analyzed using the ATSAS v3.2.1 package, namely Primus software (Manalastas-Cantos *et al.*, 2021).

Analytical ultracentrifugation (AUC) in sedimentation velocity mode was performed using a ProteomeLab XL-I analytical ultracentrifuge (Beckman Coulter, Indianapolis, IN, USA) equipped with an An-60 Ti rotor. Samples of ZmADK2 and PpADK1 were diluted in 20 mM Tris–HCl pH 7.5 with 150 mM NaCl, 10 mM MgCl_2_, 10 µM DTT, and 1% glycerol, and equilibrated at 4 °C overnight. Absorbance data were collected at 25 °C and at a rotor speed of 168 000 *g*. Scans were collected at 280 nm at 5 min intervals and 0.003 cm spatial resolution in continuous scan mode. The partial specific volume of the protein and the solvent density and viscosity were calculated from the amino acid sequence and buffer composition, respectively, using the Sednterp software (http://bitcwiki.sr.unh.edu). The data were analyzed with the continuous c(s) distribution model implemented in the program SEDFIT 15.01b, using a confidence level of 0.95 for the regularization procedure (Schuck, 2000). The plots of c(s) distributions were created in GUSSI 1.3.1 (Brautigam, 2015).

The hydrodynamic diameter was determined by dynamic light scattering (DLS) in 20 mM Tris–HCl pH 7.5 and 1 mM MgCl_2_ alone and in the presence of 2 mM ATP or 2 mM diadenosine pentaphosphate (AP5A) at 22 °C. Measurement was performed using the Zetasizer Nano ZS (Malvern Instruments, UK). Hydrodynamic diameter values were calculated by Zetasizer Software v7.13 (173° angle measurement, approximation fit to the sphere).

### Crystallization and structure determination

Crystallization conditions of all three maize ADKs were screened using Qiagen kits (www.qiagen.com) and Morpheus I screen (https://moleculardimensions.com) with a Cartesian nanodrop robot (Genomic solutions) or a Mosquito (SPT Labtech). Crystals of ZmADK2 were obtained in hanging drops by mixing equal volumes of protein solution (9 mg ml^–1^) and a precipitant solution containing 2.1 M ammonium sulfate, 0.2 M NaF, and 0.1 M sodium acetate at pH 5.5. Crystals were soaked with 10 mM AMP-PCP for 10 min. ZmADK3 (11 mg ml^–1^) was co-crystallized with 10 mM AP5A in a precipitant solution containing 20% polyethylene glycol (PEG) 8000, 0.5 M LiCl in 0.1 M Tris–HCl buffer at pH 8.0. PpADK1 (23.6 mg ml^–1^) was co-crystallized in the presence of 5 mM Ado and 5 mM ADP in a precipitant solution containing a 37.5% mixture of 2-methyl-2,4-pentanediol (MPD), PEG1000, and PEG3350 with 0.1 M carboxylic acids in 0.1 M MES/imidazole buffer pH 6.5. Crystals were transferred to a cryoprotectant solution composed of the mother liquor supplemented with 30% glycerol (ZmADK2) or 20% PEG400 (ZmADK3 and PpADK1), and flash-frozen in liquid nitrogen.

Diffraction data were collected at 100 K on the PROXIMA 1 and 2 beamlines at the SOLEIL synchrotron (www.synchrotron-soleil.fr). Intensities were integrated using the XDS program (Kabsch, 2010) and further reprocessed by Staraniso (Tickle *et al.*, 2016). Data quality was assessed using the correlation coefficient CC_1/2_ (Karplus and Diederichs, 2012) (Supplementary Table S2). Crystal structures were determined by performing molecular replacement with Phaser (Storoni *et al.*, 2004) using the structure of human ADK (PDB 1BX4, Mathews *et al.*, 1998; PDB 2I6B, Muchmore *et al.*, 2006) as a search model. Models were refined with NCS restraints and TLS using Buster 2.10 (Bricogne *et al.*, 2011) and with ligand occupancies set to 1. Electron density maps were evaluated using COOT (Emsley and Cowtan, 2004). MolProbity was used for structure validation (Chen *et al.*, 2010). Molecular graphics images were generated using PYMOL v 2.5 (www.pymol.org). Ligand interactions were analyzed using Discovery Studio Visualizer (BIOVIA, San Diego, CA, USA).

### Docking of cytokinin riboside into the active site of plant ADKs and human ADK

*In silico* docking was performed to compare the binding of Ado and iPR to the active site of plant ADK in open (ZmADK2) and closed conformation (ZmADK3, PpADK1) with that of human ADK (PDB 1BX4) in closed conformation, using FLARE v 8.0 (Cheeseright *et al.*, 2006; Bauer and Mackey, 2019; http://www.cresset-group.com/flare/). The proteins were prepared for docking using rule-based protonation predicted for pH 7.0 and intelligent capping. Energy grids for docking were 12×12×12 Å in dimension and centered on the ribose moiety of the Ado ligand. Docking calculations were carried out by the Lead Finder docking algorithm, with three independent docking runs and keeping the best poses overall (Kuhn *et al.*, 2020). The resulting ligand orientations and conformations were scored based on their binding free energies and the Lead Finder rank score (Stroganov *et al.*, 2008).

### Quantitative PCR analysis

Total RNA was extracted from 3- to 13-day-old and 3-month-old maize plants (*Z, mays* cv. Celux, Morseva) using the phenol–chloroform method with TRIzol™ reagent (www.thermofisher.com). The moss *P. patens* was grown in liquid Knop’s medium and then exposed to 10 µM hormone (abscisic acid, cytokinin, or synthetic auxin), 200 mM NaCl, or 400 mM sorbitol for 4 d. RNA was extracted using the citrate/citric acid method. First-strand cDNA was synthesized using the LunaScript RT SuperMix Kit (www.neb.com). RNA from four biological replicates was transcribed in two independent reactions, and PCR was performed in triplicate. Diluted cDNA samples served as templates for quantitative PCR (qPCR) conducted with the RT Luna Universal Probe qPCR Master Mix (www.neb.com) on a Quant Studio 5 Real-Time PCR System (www.thermofisher.com). Primers and FAM-TAM probes were designed using Primer Express 3.0 software and are listed in Supplementary Table S3. Plasmids carrying *ZmADK* genes were used as a template for the calibration curve to determine the PCR efficiencies of designed probes and primer pairs, and to verify their specificity. Cycle threshold values were normalized to maize elongation factor 1α and β-actin genes (Končitíková *et al.*, 2015).

### Preparation of ZmADK-overexpressing lines in *A. thaliana*

ORFs of three *ZmADK* genes (*Z. mays* cv. Cellux 225) were cloned into the pENTR2B cloning vector (www.thermofisher.com) using *Sal*I and *Xho*I restriction sites and then subcloned into the pOpON2.1 vector possessing dexamethasone-inducible expression *in planta* and derived from the pOpOff2 vector (Wielopolska *et al.*, 2005) using Gateway LR cloning (vector/insert ratio 1/1). These constructs were introduced into *Agrobacterium tumefaciens* strain GV3101 by electroporation and then to *A. thaliana* (Col-0 ecotype) using a floral dip method. In the T_0_ generation, resistant plants were selected on Murashige and Skoog (MS) medium with kanamycin. Resistant plants were genotyped with specific primers for *ZmADK* genes and the *actin 8* gene (AT1G49240) as an internal control. At least 20 transgenics for each *ZmADK* gene were grown, and lines with multiple insertions or silenced transgenics were discarded in the next T_1_ generation. In the T_2_ generation, homozygous lines were selected based on segregation. Finally, at least three T_3_-independent homozygous and mono-locus transformant lines harboring the *pOpOn2.1:*:*ZmADK* construct were selected for each *ZmADK* gene for phenotype and metabolite analyses. The promoter function was tested by β-glucuronidase (GUS) staining upon immersion of 7-day-old seedlings [wild type (WT) and transgenic plants] in half MS medium supplemented with 10 mM dexamethasone. The solution was removed after 72 h and plants were GUS stained using a GUS reporter gene staining kit (www.sigmaaldrich.com). Positive GUS staining served as validation of the functional transgenic line.

### Western blot analysis

Plant samples were frozen in liquid nitrogen and homogenized using an oscillating mill Retsch MM 400 (www.retsch.com) for 2 min. Samples were boiled with 2× Laemmli buffer for 10 min at 100 °C. NuPAGE electrophoresis was performed in MES buffer on a 4–12% Bis-Tris gel in the presence of a NuPAGE reducing agent (www.thermofisher.com). Proteins were electrotransferred to a polyvinylidene fluoride (PVDF) membrane (www.merckmillipore.com) under semi-dry conditions on a Trans-Blot Turbo system (www.bio-rad.com) and using a Trans-Blot Turbo RTA mini PVDF transfer kit. The membrane was washed in a blocking buffer [2% polyvinylpyrrolidone (PVP)-40, 0.1% (w/v) Tween-20 in phosphate-buffered saline (PBS)] and incubated with 1000 times diluted 6×His-tag monoclonal antibody (HIS.H8, www.thermofisher.com) for 1 h. Membranes were further reacted with mouse IgGκ BP–horseradish peroxidase (HRP) conjugate (www.scbt.com) (diluted 1:2000) and visualized with Clarity Max ECL Western Blotting Substrates (BioRad) staining solution.

### Determination of purine and cytokinin metabolites

Two-week-old *ZmADK A. thaliana* overexpressors were induced by 20 µM dexamethasone and, at 72 h after the treatment, samples were collected and purified in triplicate (10 mg FW per sample). For quantification of the purine bases, ribosides, and monophosphates, the samples (20 mg FW per sample) were homogenized, extracted in cold water with 25% ammonia (ratio 4:1), and purified by solid-phase extraction using an Oasis MAX anion-exchange column (30 mg/1 ml) (www.waters.com) with the addition of stable iosotope-labeled internal standards including [^13^C_5_]Ade, [^15^N_5_]Ado, [^15^N_4_]Hyp, [^15^N_2_]Xan, and [^15^N_4_]Ino (www.medchemexpress.com) (Kopečná *et al.*, 2013). All samples were analyzed using a liquid chromatograph SciexExionLC system coupled to a QTRAP6500+ tandem mass spectrometer (www.sciex.com) (LC-MS/MS) using an aminopropyl column (Luna 3 μm NH_2_, 100×2 mm) (www.phenomenex.com) according to a previous protocol (Karlíková *et al.*, 2016). Quantification of cytokinin metabolites was performed according to the described method (Svačinová *et al.*, 2012), including modifications (Plačková *et al.*, 2017). Samples (10 mg FW per sample) were homogenized and extracted in 1 ml of modified Bieleski buffer (60% MeOH, 10% HCOOH, and 30% H_2_O) together with a cocktail of stable isotope-labeled internal standards (0.2 pmol of cytokinin bases, ribosides, *N*-glucosides, 0.5 pmol of cytokinin *O*-glucosides, and nucleotides were added per sample). The extracts were purified by an Oasis MCX cation-exchange column (30 mg/1 ml) (www.waters.com), and then cytokinin levels were determined by LC-MS/MS using stable isotope-labeled internal standards as a reference including D_6_-iP, [^13^C_5_]*t*Z, [^13^C_5_]*c*Z, D_3_-DHZ, D_6_-iPR, D_5_-*t*ZR, D_3_-DHZR, D_6_-iPRMP, D_5_-*t*ZRMP, D_3_-DHZRMP, D_6_-iP9G, D_5_-*t*Z9G, D_3_-DHZ9G, D_5_-*t*ZOG, D_5_-*t*ZROG, D_9_-DHZOG, D_6_-iP7G, and D_5_-*t*Z7G (www.olchemim.cz), while [^15^N_4_]*c*ZR, [^15^N_4_]*c*Z7G, and [^15^N_4_]*c*Z7G were synthesized (Tranová *et al.*, 2019). Separation was performed on an Acquity UPLC i-Class System equipped with an Acquity UPLC BEH Shield RP18 column (150×2.1 mm, 1.7 μm), and the effluent was introduced into the electrospray ion source of a triple quadrupole mass spectrometer Xevo™ TQ-S MS (www.waters.com).

### Extraction and UHPLC-MS/MS quantification of selected amino acids

Amino acids were extracted from plant samples using a previously described method (Supíková *et al.*, 2022) with modifications. The material (10 mg DW) was extracted with methanol (1 ml) containing 0.1% formic acid. The extracts were then evaporated under nitrogen, redissolved in 100 μl of 20% methanol, and analyzed by UHPLC-MS/MS (www.waters.com) with a PDA detector (Acquity Ultra Performance) coupled with a tandem mass spectrometer Synapt G2-Si (www.waters.com) comprising an electrospray and a quadrupole time-of-flight (QqTOF) mass analyzer. Extracts were injected on an ARION Polar C18 column (5 μm, 250 mm×4.6 mm, www.chromservis.eu) at 30 °C. Mobile phases A (acetonitrile) and B (5 mM formic acid) were mixed in a gradient: 0 min 5% A, 0.1 min 5% A, 7 min 10% A, 12 min 35% A, 17 min 70% A, 17.5 min 100% A, 19 min 100% A, 19.5 min 5% A, 22 min 5% A. The data were acquired in a data-dependent acquisition mode. Metabolite integration and quantification with external calibration were performed using TargetLynx (www.waters.com). All standards were purchased from Sigma-Aldrich (www.sigmaaldrich.com).

### Phenotyping of root and shoot growth

To study the root phenotype of transgenics, seeds of four independent lines and the WT were germinated on half MS medium. Stratified 3-day-old seedlings were transferred onto square Petri dishes containing 20 µM dexamethasone and grown for the next 11 d on the MS medium supplemented with 0.8% gellan gum without ammonium nitrate (No N) and standard (Normal N). Plants were grown at 21 °C, 70% relative humidity, and light intensity of 110 µmol m^–2^ s^–1^ with a photoperiod of 16/8 h. The length of the primary root, total root area, and number of lateral roots were monitored daily by an RGB camera on vertical plates. Growth parameters were calculated. The rosette area was followed using the 24-well plates. At least 24 seedlings (biological replicates) per line and growth condition were included in each experiment. In the case of the WT, 48 seedlings were analyzed. Image analysis was performed using FIJI (Schindelin *et al.*, 2012), and the resulting values were statistically analyzed using a *t*-test with Statistica software (StatSoft, USA).

## Results

### Maize ADKs are monomeric, while moss ADKs are dimeric in solution

**Fig. 2.**
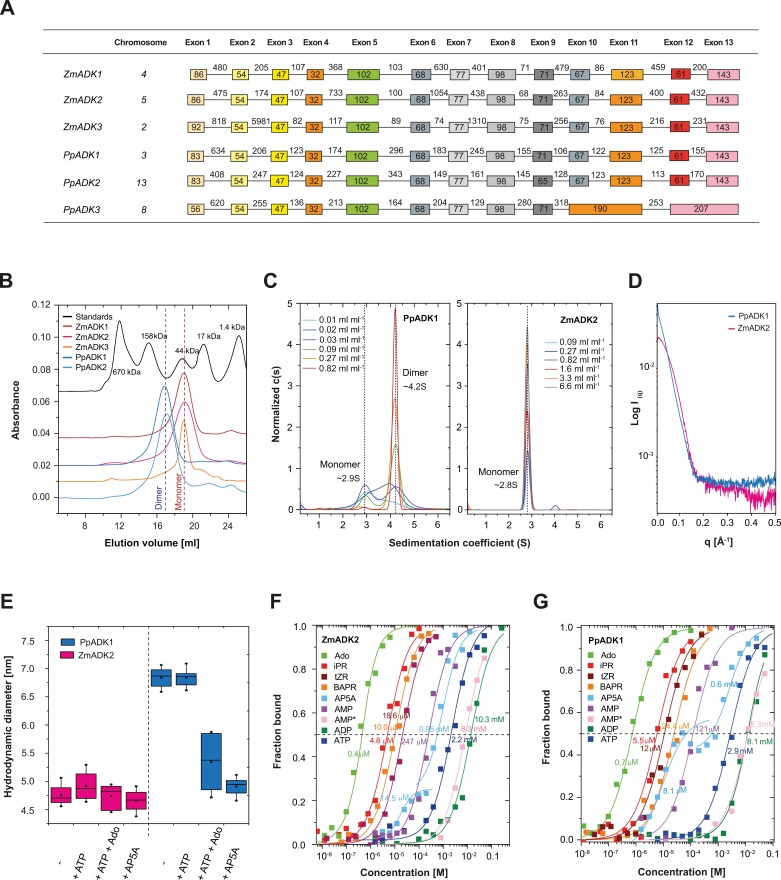
Molecular and binding properties of plant ADKs. (A) An overview of *ADK* gene models based on cloning in this work and genomic DNA sequences available at Phytozome (https://phytozome-next.jgi.doe.gov/). (B) Gel permeation chromatography profiles of five plant ADKs on a Superdex 200 10/30 HR column in 20 mM Tris–HCl buffer, pH 7.5, 100 mM NaCl, with calibration performed using a gel filtration standard (Bio-Rad). (C) The analytical ultracentrifugation plots of c(s) distributions for ZmADK2 and PpADK1 as analyzed in sedimentation velocity mode. Performed in 20 mM Tris–HCl pH 7.5 with 150 mM NaCl, 10 mM MgCl_2_, 10 µM DTT, and 1% glycerol. Scans were collected at a rotor speed of 168 000 *g* at 280 nm at 5 min intervals and 0.003 cm spatial resolution in continuous scan mode. (D) Plots of SAXS data measured with selected ADKs. Buffer values were subtracted. (E) Hydrodynamic diameter determined by DLS for selected ADKs. Measurements for apoforms were in 20 mM Tris–HCl pH 7.5 and 1 mM MgCl_2_ with 2 mM ATP or 2 mM AP5A at 22 °C. (F, G) Binding affinity curves of ZmADK2 and PpADK1 for selected riboside ligands, ADP, ATP, and AP5A. Data were measured by MST in 50 mM HEPES buffer pH 7.5, 1 mM MgCl_2_, and 0.2% Tween.

Maize and moss genomes (https://phytozome-next.jgi.doe.gov/) each has three putative *ADK* genes. Maize *ADK* genes coding for ZmADK1, ZmADK2, and ZmADK3, respectively, are located on chromosomes 4 (Zm00001d051157), 5 (Zm00001d017271), and 2 (Zm00001d003017), and contain 13 exons (Fig. 2A). We cloned three maize ORFs to produce these recombinant proteins in bacteria. ZmADK1 (342 amino acids) shares a sequence identity of 96.5% with ZmADK2 (342 amino acids) and 87.2% with ZmADK3 (344 amino acids). Two moss *ADK* genes, each composed of 13 exons, are located on chromosomes 3 (Pp3c3_10800) and 13 (Pp3c13_10550), while the third one is on chromosome 8 (Pp3c8_25260) and comprises 11 exons. We cloned the ORFs of *PpADK1* and *PpADK2* and expressed them in *E. coli*; *PpADK3* cloning was unsuccessful. PpADK1 (343 amino acids) and PpADK2 (341 amino acids) share <70% sequence identity. Although ADK activity in moss and the sequence of PpADK1 have been reported in the past (von Schwartzenberg *et al.*, 1998), there has been no in-depth characterization of these enzymes so far.

We purified the five above N-terminal His-tagged ADK isoforms (theoretical molecular weight [MW] of ~39 kDa) and investigated their molecular properties. Gel permeation chromatography analysis showed that all three maize ADKs appeared to be monomeric (MW 38±3 kDa) (Fig. 2B) as well as dimeric in a small proportion at higher concentrations (30 mg ml^–1^, Supplementary Fig. S1). In contrast, both moss ADKs were observed to dimerize already at a low concentration from 0.01 mg ml^–1^ (0.25 µM) and became mainly dimeric (MW 81±4 kDa) above 0.3 mg ml^–1^ (7.7 µM) (Fig. 2B). Thermostability measurements by nanoDSF revealed similar melting temperatures (*T*_m_) between 53 °C and 64 °C (Supplementary Fig. S1) for all isoforms, with a minor stabilizing effect of ATP (up to 4 °C). Notably, ZmADK2 and PpADK2 exhibited slightly greater stability with a *T*_m_ of 64 °C.

Measurements by AUC in sedimentation velocity mode, SAXS, and DLS revealed differences between the selected and the two most readily available representatives, ZmADK2 and PpADK1 (Fig. 2C–E). AUC data showed that the dominant component in the ZmADK2 sample is the monomer, with a sedimentation coefficient value of ~2.8 S. The MW estimate of 36–39 kDa determined using the Svedberg equation corresponds to the expected monomer mass of 39 kDa. In contrast, PpADK1 was monomeric at a low concentration, with a sedimentation coefficient of ~2.9 S, and dimeric at a higher concentration (>0.3 mg ml^–1^), with a sedimentation coefficient of ~4.2 S (Fig. 2C). Guinier analysis of SAXS data (Fig. 2D) using ATSAS software (Manalastas-Cantos *et al.*, 2021) yielded a radius of gyration (*R*_g_) of 36.77±0.15 Å for PpADK1, in contrast to 22.68±0.02 Å for ZmADK2. Subsequently, the calculated Bayesian miolecular weight estimate was 91.2±4.6 kDa and 43.8±3.4 kDa for PpADK1 and ZmADK2, respectively. These estimates are in good agreement with dimeric and monomeric states, respectively.

### Moss ADKs are dimeric upon ATP binding and monomeric upon both ATP and substrate binding

DLS revealed a difference in the hydrodynamic diameter (d_H_) between PpADK1 (d_H_ of 6.9±0.14 nm) and ZmADK2 (d_H_ of 5.0±0.15 nm) (Fig. 2E). While the presence of ATP did not affect particle diameter compared with the apoform for both enzymes, the concurrent addition of Ado substrate with ATP induced a shift towards a smaller diameter of PpADK1 (d_H_ of 5.6±0.50 nm). Moreover, incubation with the inhibitor AP5A, mimicking bound Ado with ATP, resulted in the same small size of PpADK1 (d_H_ of 5.1±0.12 nm). These data indicate that the PpADK1 dimer dissociates upon ternary complex formation.

### Maize ADKs are more active than moss enzymes

Enzyme activity was first measured using both HPLC (Moffatt *et al.*, 1991) and a coupled reaction assay (Blondin *et al.*, 1994). Later, the coupled reaction was favored because it yielded higher specific activities. We screened the activities with Ado and several cytokinin ribosides at a saturating 100 µM concentration (Table 1). Although all five plant ADKs exhibited the highest activities with Ado, all three maize ADKs were much more active (~30–40 nkat mg^–1^) than both moss enzymes (~4–5 nkat mg^–1^). Activities with cytokinin ribosides such as iPR, *t*ZR, or 6-benzylaminopurine riboside (BAPR) were comparable within each isoform and up to 10 times lower than those with Ado. Because iPR and *t*ZR are *N*^6^-isopentenylAdo derivatives, we also prepared four other Ado derivatives (Supplementary Figs S2–S6), namely *N*^6^-isopropylAdo, *N*^6^-isobutylAdo, *N*^6^,*N*^6^-dimethylAdo, and *N*^6^-methylAdo, which all have a shorter side chain than naturally occurring cytokinins. All were phosphorylated at rates comparable with those observed for cytokinin ribosides. No significant variation was observed among the studied ADK isoforms (Table 1). The *K*_m_ values for Ado were in the high nanomolar range (0.5–0.7 µM) for all five ADKs, whereas those for iPR were in the low micromolar range (4–12 µM) (Table 2). Maize ADKs displayed catalytic efficiency (*k*_cat_/*K*_m_) values 50- to 150-fold higher for Ado than for iPR (Table 2), similar to moss ADKs.

**Table 1.** Specific activities of five plant ADKs with several putative nucleoside substrates at 100 µM concentration

Substrate	Specific activity (nmol s^–1^ mg^–1^)				
	ZmADK1	ZmADK2	ZmADK3	PpADK1	PpADK2
Ado	30.5±0.4	40.3±1.4	30.0±1.1	5.09±0.26	4.70±0.05
iPR	2.8±0.3	4.1±0.4	5.1±0.3	0.93±0.11	0.83±0.08
* t*ZR	2.7±0.3	3.8±0.4	2.7±0.4	0.74±0.06	0.83±0.08
* BAPR*	3.3±0.2	5.1±0.6	5.3±0.4	0.84±0.04	0.89±0.07
*N* ^6^-IsopropylAdo	2.2±0.2	3.9±0.5	3.8±0.3	0.96±0.09	0.83±0.05
*N* ^6^-IsobutylAdo	2.1±0.4	4.2±0.6	4.8±0.5	0.90±0.06	0.75±0.08
*N* ^6^,*N*^6^-DimethylAdo	3.0±0.3	3.2±0.0	5.1±0.4	0.91±0.02	0.86±0.03
*N* ^6^-MethylAdo	2.7±0.2	2.9±0.3	5.1±0.4	0.89±0.00	0.86±0.03

Measurements were made in a reaction mixture of 50 mM Tris–HCl buffer pH 7.5 containing 50 mM KCl, 6 mM MgCl_2_, 300 µM PEP, 0.5 mM DTT, 200 µM NADH, 10 mM ATP, 2 U of PK, and 2.5 U of LDH at 30 °C.

**Table 2.** Kinetic parameters of plant ADKs from moss and maize for Ado and iPR

Enzyme	Ado			iPR		
	*K*_m_ (µM)	*k*_cat_ (s^–1^)	*k*_cat_ */K*_m_ (M^–1^ s^–1^)	*K*_m_ (µM)	k_cat_ (s^–1^)	*k*_cat_/*K*_m_ (M^–1^ s^-1^)
ZmADK1	0.62±0.11	1.14±0.04	1.8±0.3×10^3^	8.5±0.7	0.10±0.002	1.2±0.4×10^1^
ZmADK2	0.65±0.09	1.55±0.04	2.4±0.3×10^3^	9.8±1.5	0.19±0.007	2.0±0.3×10^1^
ZmADK3	0.51±0.04	1.18±0.03	2.3±0.8×10^3^	4.3±0.4	0.22±0.007	5.0±1.8×10^1^
PpADK1	0.71±0.05	0.21±0.01	3.0±0.7×10^2^	12.1±2.5	0.03±0.002	2.5±0.6
PpADK2	0.54±0.05	0.18±0.01	3.3±0.8×10^2^	4.0±0.3	0.03±0.001	7.3±0.4

Saturation curves were measured by a coupled reaction with PK and LDH in 50 mM Tris–HCl buffer pH 7.5 containing 50 mM KCl, 6 mM MgCl_2_, 300 µM PEP, 0.5 mM DTT, 200 µM NADH, and 10 mM ATP at 30 °C. Kinetic constants *K*_m_ and *k*_cat_ were determined using GraphPad Prism 8.0 software (http://www.graphpad.com). SDs were calculated from three repetitive measurements.

Binding affinities (*K*_D_ values) for the tested substrates, such as cytokinin ribosides (transport form of the hormone), were further determined by MST, with the two selected representatives, ZmADK2 and PpADK1 (Fig. 2F, G; Table 3), displaying a similar binding pattern, with *K*_D_ values close to *K*_m_ values. The best affinity was observed for Ado (*K*_D_ of 0.4–0.7 µM), followed by iPR, *t*ZR, and BAPR with *K*_D_ values ranging between 5 µM and 25 µM. Interestingly, the large molecule of the inhibitor AP5A was bound in two sequential steps, the first *K*_D_ value corresponding to its binding into the Ado site and the second corresponding to its binding into the ATP site. For PpADK1, the lower *K*_D_ value was 8.1±1.1 µM, while the higher *K*_D_ value was 0.63±0.12 mM. Affinities for the co-substrate ATP and its product ADP were relatively weak and in the low millimolar range. The measured *K*_D_ values for ATP were 2.2±0.6 mM with ZmADK2 and 2.9±0.7 mM with PpADK1.

**Table 3.** Binding affinities and *K*_m_ values of several Ado and ATP analogs with ZmADK2

Nucleoside/nucleotide ligand	*K*_D_ (µM)	*K*_m_ (µM)	*k*_cat_ (s^–1^)
Ado	0.40±0.14	0.65±0.09	1.55±0.04
	1480±230*		
*N* ^*6*^-MethylAdo	8.7±2.5	10.7±1.5	0.24±0.02
*N* ^6^,*N*^6^-DimethylAdo	12.3±4.6	11.2±2.8	0.20±0.02
*N* ^6^-IsopropylAdo	52.8±13.9	54.8±5.8	0.19±0.01
*N* ^6^-IsobutylAdo	45.9±9.3	70.4±8.9	0.21±0.01
iPR	4.8±1.1	9.8±0.4	0.19±0.01
2′-DeoxyAdo	505±94	506±39	0.46±0.02
3′-DeoxyAdo (cordycepin)	175±9	173±11	0.43±0.01
7-DeazaAdo (tubercidin)	2.2±0.7	3.4±0.4	0.17±0.01
AraA (vidarabine)	>>500, ND	524±51	0.24±0.02
Inosine	440±180	420±44	0.57±0.02
Guanosine	217±107	218±23	0.48±0.02
Xanthosine	166±77	178±10	0.55±0.01
Uridine	>>5000, ND	>>1000, ND	0.15±0.03
Cytidine	286±10	313±28	0.23±0.01
AP5A	14.5±9.2	–	–
	949±46*		
AMP	247±71	ND	ND
	8299±2070*		
ADP	10 300±1190	ND	ND
ATP	2200±620	ND	ND

Activities were measured in a coupled reaction with PK and LDH at 30 °C in 50 mM Tris–HCl buffer pH 7.5 using 10 mM ATP. Affinity was measured by MST in 50 mM HEPES buffer pH 7.5, 1 mM MgCl_2_, and 0.1% Tween. ND, not determined; *second *K*_D_ value.

We analyzed the binding properties of several other purine ribosides, deaza, and deoxy derivatives with the most active enzyme, ZmADK2 (Table 3; Supplementary Fig. S1). None of the ligands reached *k*_cat_ values or had an affinity as high as Ado. The four Ado derivatives were synthesized to analyze the effect of the side chain on the activity and binding. While *k*_cat_ values were comparable, *N*^6^-methylAdo and *N*^6^,*N*^6^-dimethylAdo had slightly lower affinities than iPR, whereas the two longer compounds displayed a 10-fold weaker affinity than iPR. Similar to the interactions with AP5A, Ado with AMP displayed two binding events, suggesting that they can bind into both the substrate and ATP sites. Ado and AMP exhibited the second binding event in the low millimolar range [*K*_D(2)_ values of 1.48±0.23 mM and 8.3±2.0 mM, respectively] for their binding in the second site.

AraA was a very poor ligand, with a *K*_D_ value exceeding 500 µM, making it challenging to measure due to solubility issues. Both 2′-deoxyAdo and 3′-deoxyAdo derivatives displayed ~1200- and 400-fold weaker affinity compared with Ado, with *K*_D_ values of 505±94 µM and 175±9 µM, respectively (Table 3). This underscores the crucial role of both the O2' and O3' hydroxyl groups in nucleoside binding. In contrast, binding of the 7-deazaAdo derivative was significantly less affected, displaying a *K*_D_ value of 2.2±0.7 µM, which is ~5.5-fold weaker affinity than Ado. ZmADK2 could also bind and phosphorylate oxopurine nucleosides such as inosine, xanthosine, or guanosine. Measured *k*_cat_ values were around one-third of what was observed with Ado. *K*_D_ and *K*_m_ values fell within the high micromolar range, indicating that these compounds are relatively weak substrates.

### The crystal structure of maize ADK2 reveals a unique dimer formation among plant ADKs

Of the two selected representatives, ZmADK2 and PpADK1, we only obtained crystals of the apo form of ZmADK2. We solved the structure (PDB 8RF7) by molecular replacement using the structure of the human ADK in the open conformation (PDB 2I6B; Muchmore *et al.*, 2006) as a search model (Supplementary Table S2). ZmADK2 shares ~58% sequence identity with human ADK (HsADK; Supplementary Fig. S7).

**Fig. 3.**
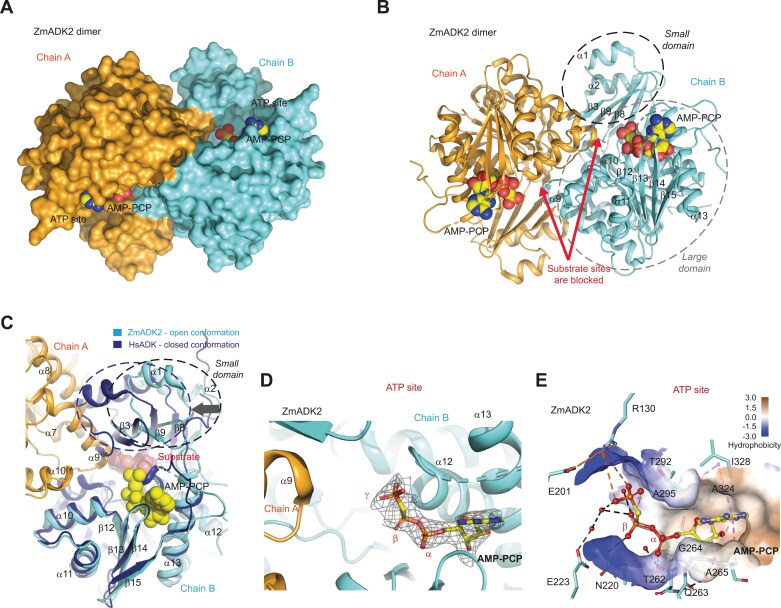
Dimeric structure of plant maize ADK2 in open conformation. (A) Dimeric structure of ZmADK2 (this work, PDB 8RGJ) in surface representation colored in cyan/orange with the bound AMP-PCP in yellow. (B) Cartoon representation of the dimeric structure of ZmADK2 with bound AMP-PCP, featuring a topology description. Both small and large domains are labeled. Red arrows indicate the position of each substrate-binding site blocked by the neighboring subunit. (C) Superposition of ZmADK2 in the open conformation (chain B, cyan) with HsADK in the closed conformation (PDB 2I6A, dark blue; Muchmore *et al.*, 2006) containing bound nucleoside. The black arrow denotes the rotation of the small domain. Chain A of the ZmADK2 dimer is in orange. (D) Binding of the AMP-PCP in the ATP site of ZmADK2 in its annealing *F*_o_–*F*_c_ omit map (black mesh) contoured at 2.5 σ. (E) Binding interactions of AMP-PCP in ZmADK2 and hydrophobicity of the ATP site. Hydrogen bonds are shown in black dashed lines, while electrostatic and hydrophobic interactions are shown as orange and pink dashed lines.

Crystallization conditions of ZmADK2 favored the dimeric form to be present in the asymmetric unit (Fig. 3A, B), which contains two ZmADK2 molecules, each in the open conformation, and resembles that of HsADK, with an average root mean square deviation (RMSD) of ~1.2 Å and 1.4 Å for all Cα atoms. Each monomer is composed of two domains: a large 10-stranded α/β Rossmann-like nucleotide-binding domain (residues 1–14, 64–117, and 137–340) comprising the ATP-binding site; and a small five-stranded α/β domain (residues 17–61 and 120–134). The small domain encompases β2, β3, β4, β8, and β9 sheets, plus α1 and α2 helices. Both domains are connected via four peptide hinges (Leu15–Leu16, Gly62–Gly63, Thr118–Gly119, and Asn135–Leu136). The nucleoside substrate pocket is located in a cleft between the two domains.

The dimer interface covers an area of 1577 Å^2^ as calculated by the PISA server (www.ebi.ac.uk/pdbe/pisa/) involving ~50 residues per monomer and making it characteristic of biological interactions (Janin *et al.*, 2007) (Supplementary Fig. S7). The solvation free energy gained upon interface formation is –19.7 kcal mol^–1^. This involves side chains of both domains: the regions 35–43 and 130–136 of the small domain and the regions 142–145, 167–174, 196–206, and 222–236 of the large domain. So far, such a dimer arrangement has not been reported for any other ADK.

Substrate binding induces the closed conformation, resulting in a rotation of the small domain by ~30°, as documented in previous studies (Mathews *et al.*, 1998; Muchmore *et al.*, 2006). Remarkably, the dimer interface prevents displacement of the small domains. Indeed, the superposition of HsADK in the closed conformation (PDB 1BX4; Mathews *et al.*, 1998) on one subunit of the ZmADK2 dimer showed that helices α8 and α9 of the large domain of the other subunit block the rotation of its small domain (α1–β3–α2 segment) and thus proper binding of the nucleoside substrate (Fig. 3C). Therefore, to be active, moss and maize ADK dimers must be dissociated into monomers.

### ATP binding has no effect on the dimeric state

In agreement with DLS measurements showing that ATP binding did not affect the oligomeric state of plant ADKs, soaking crystals of the apo ZmADK2 dimer with the non-hydrolyzable ATP analog led to a dimeric complex, with AMP-PCP (PDB 8RGJ) occupying the ATP-binding site. Its γ-phosphate moiety is oriented toward the substrate-binding site where the ribose moiety of a nucleoside substrate would be located (Fig. 3D, E). The Ado moiety of ATP is surrounded by hydrophobic residues. Indeed, its ring is located between Ile328 and Ala295 on one side, and Ala265 with Gly264 on the other, forming multiple hydrophobic contacts (Fig. 3E). The O2' atom of the ribose moiety is bound directly to the SG atom of Cys321, and the O3' atom is H-bonded via a water molecule to the main chain oxygen atom of Gly264 and the nitrogen of Gln263. The α-phosphate group forms a hydrogen bond with the OG1 atom of Thr262. The β-phosphate group is H-bonded to Asn220, and both the β- and γ-phosphate groups interact with Glu223 via two water molecules. Finally, the negatively charged γ-phosphate moiety establishes an electrostatic interaction with the positively charged side chain of Arg130.

The role of Asn220 and Glu223 residues was studied by site-directed mutagenesis on ZmADK3 (Table 4). The N222A variant (ZmADK3 numbering is shifted by two amino acids) showed a 7-fold lower affinity for ATP with half the activity compared with the WT. Remarkably, the E225A variant displayed an ~6-fold lower affinity for ATP and only 6% activity compared with the WT.

**Table 4.** Characterization of active site variants of ZmADK3.

Variant	Specific activity (nkat mg^–1^)	Relative rate (%)	*K*_D_	
			Ado (µM)	ATP (mM)
WT ZmADK3	31.0±0.8	100.0	0.28±0.05	2.9±1.1
D19A	3.3±0.4	10.6	989±161	4.9±1.5
R132A	7.4±0.3	23.7	0.27±0.04	8.5±2.1
N222A	13.4±0.7	43.4	0.30±0.13	20.7±4.4
E225A	1.9±0.4	6.2	0.32±0.09	16.0±4.5
N295A	10.1±0.1	32.5	274±10	41.8±10
D299A	1.8±0.1	5.8	10.3±4.1	9.1±2.1

Activities were measured with 100 µM Ado in a coupled reaction with PK and LDH at 30 °C in 50 mM Tris-HCl buffer pH 7.5 using 10 mM ATP. *K*_D_ values were measured by MST in 50 mM HEPES buffer pH 7.5, 1 mM MgCl_2_, and 0.1% Tween.

### Closed conformation is associated with the monomeric state and ternary complex formation

**Fig. 4.**
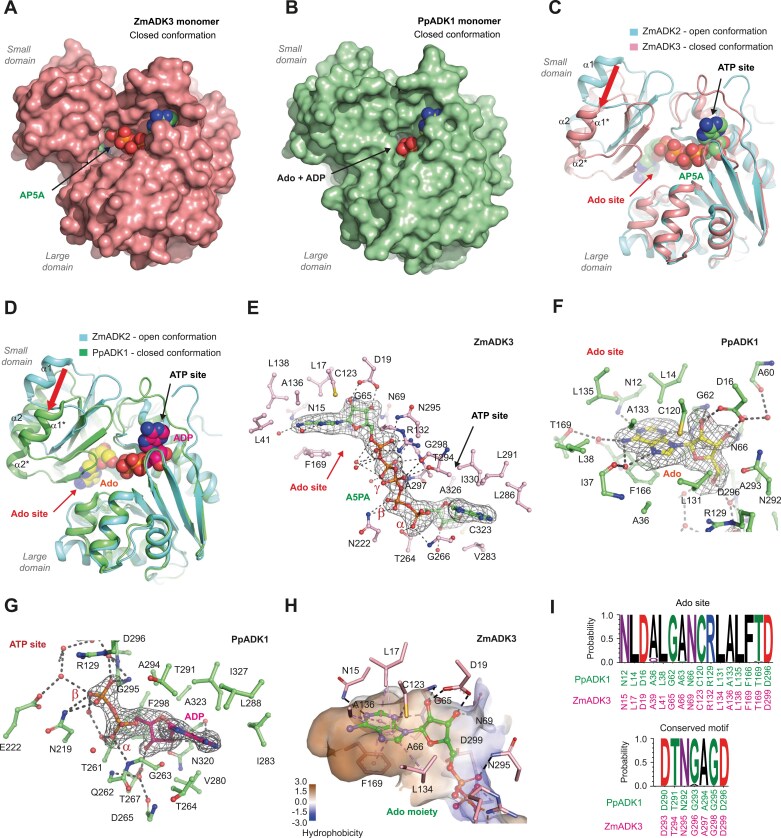
Monomeric structure of plant ADKs in closed conformation. (A) The monomeric structure of ZmADK3 (this work, PDB 8RPA) colored in pink with the bound AP5A in green. (B) The monomeric structure of PpADK1 (this work, PDB 9FW6) colored in green with both bound Ado and ADP. (C) Superposition of ZmADK2 in an open conformation (cyan) with ZmADK3 with AP5A in the closed conformation (pink). The red arrow indicates the shift of the small domain. (D) Superposition of ZmADK2 in an open conformation (cyan) with PpADK1 with Ado and ADP in the closed conformation (green). The red arrow indicates the shift of the small domain. (E) Interactions between ZmADK3 and the bound AP5A inhibitor. AP5A (in green) are shown in its annealing *F*_o_–*F*_c_ omit map (black mesh) contoured at 3.0 σ. (F) Interactions of Ado ligand, colored in yellow, in the Ado-binding site of PpADK1 with the enzyme. The Ado is shown in its annealing *F*_o_–*F*_c_ omit map (black mesh) contoured at 3.0 σ. (G) Binding of the ADP ligand in the ATP site of PpADK1 in its annealing *F*_o_–*F*_c_ omit map (black mesh) contoured at 3.0 σ and its interactions with the enzyme. (H) Hydrophobic and hydrogen-binding interactions in the adenosine pocket of ZmADK3 with AP5A ligand. (I) The conservation of amino acid residues in plant ADKs forming the Ado site and the conserved motif involved in binding of the β- and γ-phosphate of ATP. The sequence logo was made using WebLogo 3 (http://weblogo.threeplusone.com).

Co-crystallization of ZmADK3 and PpADK1 in the presence of AP5A and a mixture of Ado and ADP, respectively, led to two high-resolution crystal structures of a ternary complex (PDB 8RPA and 9FW6; Supplementary Table S2; Fig. 4A, B). The asymmetric unit contains one monomer for the ZmADK3–AP5A complex (Fig. 4A) and two monomers for the PpADK1–Ado–ADP complex, all in the closed conformation. Structural comparison between ZmADK2 (open state) and the closed state of both ZmADK3 and PpADK1 reveals that AP5A (or Ado) binding induced a rigid-body motion of the smaller domain towards the large domain, resulting in the closed conformation. Two helices, α1 and α2, shift by up to 12 Å to shield the Ado site from the solvent (Fig. 4C, D).

The electron density map of the bisubstrate analog AP5A in ZmADK3 is very well defined (Fig. 4E). The ligand binds in the groove between the large and small domains. The electron density maps of Ado and ADP in PpADK1 show clearly that both substrates bind in the Ado- and ATP-binding sites, respectively (Fig. 4F, G). When superimposed, Ado in PpADK1 overlaps the Ado part of AP5A in a highly hydrophobic pocket (Fig. 4H). Analysis of all available plant ADK sequences in the Phytozome 13 database revealed a high conservation of residues forming the Ado pocket, as illustrated in Fig. 4I. This finding explains the remarkably similar substrate properties observed among the studied ADK isoforms. The unique DTN(G/A)AGD motif near the C-terminus (Supplementary Fig. S7), typical for ADKs, is fully conserved and comprises residues involved in binding β- and γ-phosphates of ATP.

The purine ring of Ado stacks on the phenyl ring of Phe169 (in ZmADK3 numbering; PpADK1 numbering is shifted lower by three amino acids) and establishes hydrophobic interactions with Leu17, Ala66, Cys123, Leu134, and Ala136. The N1 and N3 atoms of the purine ring are H-bonded to the side chain of Asn15 and the main chain nitrogen atom of Ala66, respectively. Both O2' and O3' atoms of the ribose moiety bind to the side chain of Asp19. In addition, the O3' atom interacts with Asn69, while the O2' atom is H-bonded to the main chain nitrogen atom of Gly65. The phosphate group, transferred to Ado to form AMP, interacts with the side chains of Asn295 and Arg302.

AP5A in ZmADK3 and AMP-PCP in ZmADK2 adopt the same conformation and position in the ATP-binding site. They establish similar hydrogen and hydrophobic interactions. The adenine part of ADP in PpADK1 is slightly shifted compared with the adenine moiety of the ATP part of AP5A due to the presence of a threonine at position 264 in PpADK1 instead of an alanine (Ala265) in ZmADK3. The γ-phosphate in AP5A forms a hydrogen bond with the OG1 atom of Thr294 and the side chain of Arg132.

The crucial role of Asp19 in riboside binding was further confirmed by site-directed mutagenesis (Table 4). The D19A variant exhibited a 10-fold reduction in specific activity and a poor affinity for Ado. The *K*_D_ value for Ado shifted from 0.28 µM to 989 µM. Although Asp299 is placed behind the ribose of Ado moiety of AP5A with no direct H-bonds (Fig. 3I), it could bind the free O5' hydroxyl group. The D299A variant displayed only ~6% activity and ~37 times lower affinity for Ado than the WT. Also, the Arg residue (Arg130 in ZmADK2 and Arg132 in ZmADK3), situated between Ado and ATP pockets, plays a vital role in catalysis. This residue holds the γ-phosphate of ATP, as seen in the ZmADK2 structure (Fig. 3E), and the phosphate molecule bound to Ado during AMP formation, as observed in the ZmADK3 structure (Fig. 4E). The R132A variant displayed ~24% activity compared with the WT, and while the affinity to Ado remained unaffected, the affinity to ATP was three times lower.

Finally, *in silico* docking of cytokinin riboside (iPR) into the Ado site of ZmADK3/PpADK1 revealed enough space to accommodate the isoprenoid chain of cytokinin next to the side chains of L41/L38 and F200/F197, which can adopt different rotamer orientations. The position of iPR overlaps that of Ado (Supplementary Fig. S8), and the binding energies of both ligands were nearly identical (Supplementary Table S4).

### All *ADK* genes are highly expressed in maize, but only one in moss

**Fig. 5.**
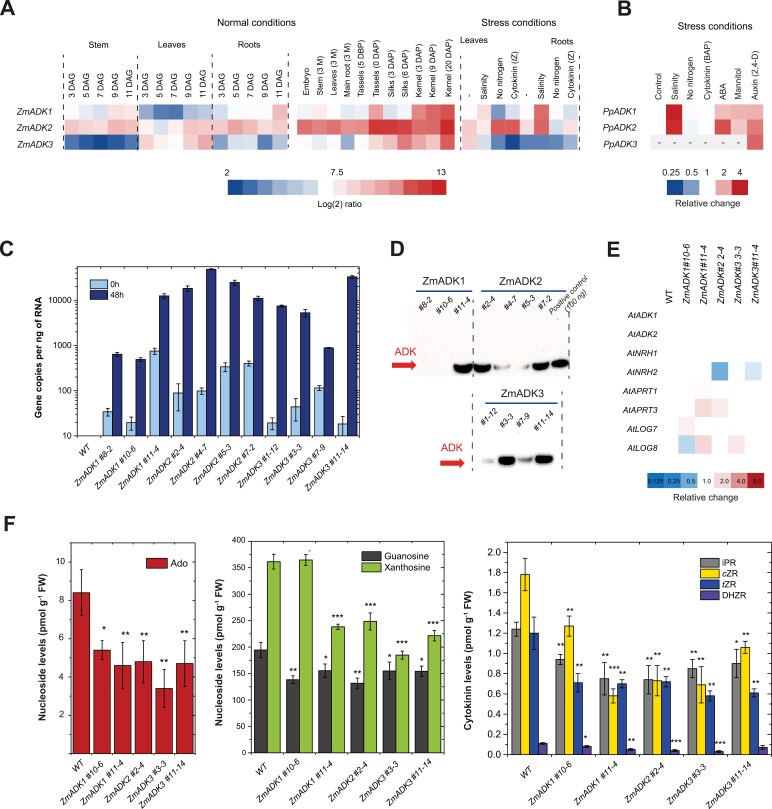
Expression of *ADK* genes in maize and moss, and characterization of *ZmADK* transgenic lines. (A) *ADK* expression in maize was followed for 2 weeks after germination and later in various organs under normal conditions (left side) and during various stresses (right side). The heat map illustrates transcript levels detected in 1 ng of total RNA. All values are expressed as log_2_ ratios. DAG, days after germination; DAP, days after pollination; DBP, days before pollination; M, months. (B) *ADK* expression in moss grown in liquid Knop’s medium and then exposed to 10 μM abscisic acid, cytokinin (BA), auxin (2,4-D), or methyl jasmonate, 200 mM NaCl, or 400 mM mannitol for 4 d. (C, D) Dexamethasone-inducible expression of *ZmADK* genes in homozygous Arabidopsis seedlings after 48 h at 14 DAG, assessed at RNA (C) and protein levels (D). The expression was tested using qPCR with FAM-TAMRA probes, and protein production was evaluated from whole plantlet extracts via western blot. The PVDF membrane was probed with a monoclonal anti-6His-tag mouse antibody and reacted with mouse IgG BP–HRP conjugate for chemiluminescent detection. (E) The expression of related genes as analyzed by qPCR using labeled probes upon 48 h dexamethasone induction of *pOpON::ZmADK* transgenic lines at 14 DAG. (F) Nucleoside levels (pmol g^-1^ FW) in induced *pOpON::ZmADK* overexpressor lines including Ado, guanosine, xanthosine, and cytokinin ribosides. Three technical replicates were performed. Asterisks indicate statistically significant differences in induced transgenic lines versus WT Col-0 in a Student’s *t*-test (*, **, and *** correspond to *P*-values of 0.05>*P*>0.01, 0.01>*P*>0.001, and *P*<0.001, respectively).

The expression of the three maize *ADK* genes was detected in roots and leaves during early developmental stages, as assessed by qPCR using FAM-TAMRA probes (Fig. 5A; Supplementary Table S5). In young seedlings, *ZmADK2* transcripts were highly abundant in stems, leaves, and roots, while *ZmADK3* transcripts were primarily found in leaves and *ADK1* transcripts were observed in roots. In older plants (3 months), *ZmADK2* expression remained high across various samples, including leaves, roots, tassels, silks, and kernels. Transcripts of *ZmADK3* were abundant in silks and kernels, while *ZmADK1* transcripts were mainly present in kernels. This expression pattern suggests a vital role for all three ADKs in developing seeds and reproductive organs containing dividing cells. Under salt stress, the expression of *ZmADK1* and *ZmADK2* increased in roots. Treatment with active cytokinin (*t*Z) did not induce significant changes in *ADK* expression levels in either leaves or roots. Although nitrogen starvation is known to up-regulate *ENT* genes and lead to increased nucleoside import from degraded RNAs (Cornelius *et al.*, 2012; Melino *et al.*, 2018), the transcript abundance of *ADK* genes was not up-regulated upon nitrogen starvation in maize.

In *P. patens* (protonemal stage) cultivated in liquid Knop medium, we observed that *PpADK1* transcripts were highly abundant, reaching ~13 800 copies ng^–1^ of RNA (Supplementary Table S6). Conversely, the amount of *PpADK2* transcripts was two orders of magnitude lower (~80 copies ng^–1^ of RNA) and transcripts of *PpADK3* were not detectable. When exposed to salt stress, the expression of both *PpADK1* and *PpADK2* was ~3-fold higher than in the control (Fig. 5B). Additionally, abscisic acid and mannitol treatments increased the expression of both genes ~2-fold. In contrast, treatment with active cytokinin (BAP) or nitrogen starvation did not induce significant changes in *ADK* expression levels, mirroring our findings in maize. The *PpADK3* transcripts were observed exclusively after treatment with the synthetic auxin 2,4-dichlorophenoxyacetic acid (2,4-D).

In summary, the genome of *Physcomitrella* lacks the *PNP* gene; *PpADK2* and *PpADK3* genes are poorly expressed; and both tested moss enzymes, PpADK1 and PpADK2, display weak activities compared with maize ADKs. These findings imply that the pathway from adenine to AMP via Ado is generally low, and the direct conversion from adenine to AMP via APTs plays a more significant role in *Physcomitrella*.

### ZmADK overexpressors contain lower levels of adenosine and cytokinin ribosides

*Arabidopsis thaliana* Col-0 plants were stably transformed with expression constructs containing cDNAs of three maize ADKs placed under the control of a dexamethasone-inducible promoter. Independent T_3_ homozygous transgenic lines were selected for each overexpression construct according to the induced gene expression level identified by qPCR (Fig. 5C). Transcript levels of *ZmADK* genes, measured 72 h after induction, displayed significant variations among the studied lines. At least three lines, namely *pOpOn::ZmADK1*#10-6, *ZmADK1*#8-2, and *ZmADK3*#7-9, had a lower number of transcript copies (<1000 per ng of RNA) than the others (>10 000 copies). Similarly, the above lines accumulated a lower amount of ZmADKs at the protein level. On the other hand, five other lines contained much higher levels of ZmADK1/2/3 (Fig. 5D). We selected both representatives with weak and strong *ZmADK* overexpression for further characterization *in planta*.

The qPCR analysis conducted on whole seedlings of all examined transgenics 2 d after induction (Fig. 5E) revealed no significant changes in the expression of the two Arabidopsis *ADK* genes, namely *AtADK1* (At3g09820) and *AtADK2* (At5g03300), or of both *NRH* genes, namely *AtNRH1* (At2g36310) and *AtNRH2* (At1g05620). Additionally, a subtle up-regulation of *AtAPT3* (At4g22570) was observed in two transgenic lines, while no significant changes were detected for *AtAPT1* (At1g27450). Two *pOpOn::ZmADK* lines showed a minor up-regulation of *AtLOG8* (At5g11950) and one line an up-regulation of *AtLOG7* (At5g06300) expression. Taken together, no clear pattern in the expression of these genes was observed.

Quantitative analysis of purine and cytokinin bases, nucleosides, and nucleotides in 2-week-old and dexamethasone-induced seedlings was conducted using UPLC-MS/MS as outlined in prior studies (Kopečná *et al.*, 2013). In all analyzed transgenic lines, there was a discernible impact on purine and cytokinin metabolism, as shown in Fig. 5F and detailed in Supplementary Table S7. These alterations were consistent with the substrate preferences of ZmADKs, leading to decreased levels of various nucleosides. Specifically, levels of Ado were reduced to ~45–65%, while cytokinin ribosides iPR, *t*ZR, *c*ZR, and DHZR were decreased to ~50–70% compared with WT Col-0 plants. Adenine levels were slightly reduced and levels of cytokinin bases remained unchanged. Although AMP levels could not be adequately assessed due to the lack of an internal standard, the levels of cytokinin monophosphates iPRMP, *t*ZRMP, *c*ZRMP, and DHZRMP were not elevated; instead, they were similar or slightly lower (by ~10%) compared with the WT. In addition to these changes, among other purine nucleosides, guanosine levels were also slightly reduced to ~80%, and xanthosine levels decreased to ~50–65% in the *pOpOn::ZmADK* transgenics compared with the WT. Among other cytokinin metabolites, no changes were observed among *N*^7^-glucosides, *N*^9^-glucosides, or *O*-glucosides (Supplementary Table S7).

### Larger roots are observed in ZmADK overexpressors under nitrogen-limiting conditions

**Fig. 6.**
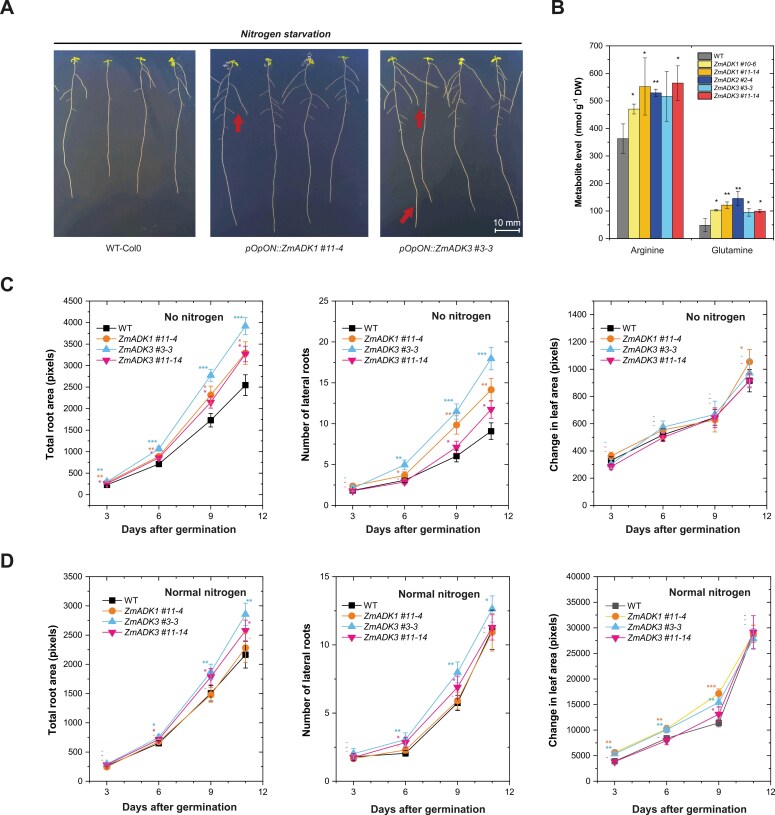
Phenotyping of *A. thaliana pOpON::ZmADK* overexpressors under abiotic stress. (A) An illustration of primary and lateral root growth in WT and *pOpOn::ZmADK3* transgenics over 9 d on MS medium without nitrogen. (B) Levels of arginine and glutamine in induced *pOpON::ZmADK* overexpressor lines upon nitrogen starvation. Asterisks indicate statistically significant differences in a Student’s *t*-test (*, **, and *** correspond to *P*-values of 0.05>*P*>0.01, 0.01>*P*>0.001, and *P*<0.001, respectively). (C) Growth of nitrogen-starved plants showing the number of lateral roots, total root area, and total rosette area. (D) Growth of plants cultivated on standard MS medium for up to 14 d. Each experiment included a minimum of 24 seedlings (biological replicates) per *ZmADK* line and growth condition. Asterisks indicate statistically significant differences in transgenic lines compared with WT controls, determined in a paired Student’s *t*-test (*, **, and *** correspond to *P*-values of 0.05>*P*>0.01, 0.01>*P*>0.001, and *P*<0.001, respectively).

Knowing that nitrogen starvation induces RNA catabolism (Kraft *et al.*, 2008), making nucleosides including Ado available, we conducted phenotyping experiments on the Arabidopsis lines overexpressing *ZmADK1/2/3*, explicitly focusing on root and rosette growth. The induced *pOpON::ZmADK* lines exhibited noticeable variations in root growth under nitrogen starvation conditions, including slightly longer primary roots and an increased number of lateral roots (Fig. 6A). Thus, we performed a detailed screening involving four transgenic *ZmADK* lines (using 24 biological replicates per line) spanning a 2 week period to obtain relevant data. Plants were cultivated under standard nitrogen conditions and nitrogen starvation. Detailed statistics for total root and leaf area are given in Supplementary Tables S8 and S9.

Notably, nitrogen-starved transgenic plants exhibited a statistically significant increase in the number of lateral roots and total root area (quantified in pixels) (Fig. 6C). At 11 d after germination, mean values for the number of lateral roots in all *ZmADK* transgenics were 30–90% higher than those of the WT. Similarly, mean values for the total root area in ZmADK1#11-4, ZmADK3#3-3, and ZmADK3#11-14 transgenic lines were from 30% to 50% higher than in the WT, with ZmADK2 #2-4 behaving similarly to the WT (not shown). In contrast, only modest changes in rosette size were observed, ranging from 89% to 116% compared with plants, but they were not statistically different (Fig. 6C). Under normal nitrogen conditions, the differences between the induced *ZmADK* transgenics and the WT were less apparent (Fig. 6D).

Finally, to assess the nitrogen released from purines under nitrogen starvation, we analyzed levels of six free amino acids, namely ornithine, Arg, Glu, Gln, Asp, and Asn (Supplementary Table S10). The induced overexpressor lines contained a higher amount of two nitrogen-rich amino acids Arg and Gln (both by ~50%, Fig. 6B). While Gln joins purine catabolism with the Krebs cycle via Glu, Arg stands at the beginning of polyamine synthesis.

## Discussion

### Plant ADKs exist as monomers and dimers

This work presents a comprehensive study of plant ADKs from maize and moss, combining *in vivo* and *in vitro* approaches. We showed that plant ADKs can form dimers *in vitro*, unlike all other homologous ADKs studied so far (Andres and Fox, 1979; Guranowski, 1979; Miller *et al.*, 1979b; Mathews *et al.*, 1998; Darling *et al.*, 1999; Schumacher *et al.*, 2000; Muchmore *et al.*, 2006; Zhang *et al.*, 2007; Kuettel *et al.*, 2011; Romanello *et al.*, 2013; Timm *et al.*, 2014). *Mycobacterium tuberculosis* ADK (MtADK) folds into a dimer not comparable with the ZmADK2 dimer, in agreement with a low 22% sequence identity between both enzymes (Supplementary Fig. S9; Long *et al.*, 2003; Reddy *et al.*, 2007). Its structure contains two additional helices in the small domain, which participate in the interface to form the active dimer (Reddy *et al.*, 2007).

We further showed that the ADK dimer can exist only when both subunits are in an open conformation, as observed in the ZmADK2 structure. When in a dimeric state, the enzyme cannot be catalytically active as the rotation of the small domain and closure of the substrate cavity for catalysis is blocked. To form the closed ternary complex and bring the two subunits close together, the enzyme must be monomeric as shown by the structures of both ZmADK3 bound to AP5A and PpADK1 bound to ADP and Ado. DLS on PpADK1 alone or in the presence of ATP with Ado or AP5A highlighted changes in the hydrodynamic diameter of the enzyme upon ligand addition. Indeed, the diameter of PpADK1 (dimeric in solution) became smaller, reaching that of the monomer and demonstrating that dimeric PpADK1 dissociates to monomers during a ternary complex formation in order to be active.

In line with the above, soaking crystals of apo open-state ZmADK2 with the substrates Ado or iPR or Ado and co-substrate analog AMP-PCP was unsuccessful due to the lack of closure of the substrate cavity within the dimer inside the crystals. Since the ATP site was accessible, we obtained a complex of ZmADK2 with AMP-PCP in the open conformation. The closed conformation was previously observed only in the structures where the substrate site was occupied, usually together with the ATP site, including human and *Toxoplasma* ADK with bound Ado (PDB 1BX4, Mathews *et al.*, 1998; PDB 1LIK, 1LII and 1LIJ, Schumacher *et al.*, 2000). This has also been observed for bound Ado with AMP-PNP (PDB 4N09) and AP5A in trypanosomal ADK structures (PDB 3OTX) (Kuettel *et al.*, 2011).

We infer that dimer dissociation is likely to be the rate-limiting step in the catalysis of both moss enzymes and it is consistent with their up to 10-fold lower kinase activities compared with the predominantly monomeric maize ADKs. How the substrate induces the dimer dissociation remains unclear. *In vivo* concentrations of Ado range from nanomolar to low micromolar (Kopečná *et al.*, 2013; Ľuptáková *et al.*, 2024), depending on factors such as plant growth conditions, phosphate availability, and the methods used for extraction. The concentrations fall within the range of *K*_D_ or *K*_m_ values reported here and from previous studies for various ADKs. Consequently, along with ATP, these concentrations can influence the oligomeric state of ADKs. Comparable Ado concentrations have also been observed in mammals (Hellsten *et al.*, 1998).

### Substrate preferences among various ADKs

Substrate preferences have been analyzed in the past for ADKs from the human placenta (Andres and Fox, 1979), rabbit liver (Miller *et al.*, 1979a), *T. gondii* (Iltzsch *et al.*, 1995; Darling *et al.*, 1999), *T. brucei* (Vodnala *et al.*, 2008), and several other species. Substrate specificity screenings with rabbit and *Toxoplasma* ADKs showed Ado as the best substrate, followed by 7-deaza, 1-deaza, and 3-deaza Ado derivatives. Poor activities of the latter two were explained by the missing H-bond interactions with the N1 and N3 atoms within the active site (Mathews *et al.*, 1998). Many substituted nucleosides have been tested for antitubercular activity against mycobacterial ADK from *M. tuberculosis* (MtADK; Long and Parker, 2006). For example, 2-methylAdo was a much better substrate and selective against human ADK (Long *et al.*, 2003). It was shown that the difference in catalytic efficiency was three orders of magnitude in favor of MtADK when phosphorylating 2-methylAdo. The activities and *K*_m_ values measured in this work for the cytokinin ribosides iPR, ZR, and BAPR with five plant ADKs (Tables 1, 2) align with previous reports for lupin, wheat, and tobacco ADKs (Chen and Eckert, 1977; Guranowski, 1979; Kwade *et al.*, 2005) as well as for two Arabidopsis ADKs (Moffatt *et al.*, 2000).

Because of the remarkable conservation of the Ado pocket among plant and mammalian ADK sequences (Fig. 3I), it is reasonable to deduce that mammalian ADKs can naturally bind plant-derived cytokinin ribosides. Indeed, rabbit ADK phosphorylates cytokinin ribosides (Miller *et al.*, 1979a). While the *K*_m_ for Ado was 0.4 µM, those for iPR, ZR, and BAPR were 7, 20, and 12 µM, respectively. The enzyme showed 69% activity with iPR, 29% with BAPR, and 6.3% with ZR compared with Ado. Our *in silico* docking analysis showed almost no differences in the binding of iPR to ZmADK3 and human ADK (Supplementary Fig. S8). Phe201 and Leu40 have to flip to leave more space for the side chain. However, the binding energies between Ado and iPR are comparable, suggesting similar binding modes. Moreover, previous analysis of rabbit ADK revealed that 2′-deoxyAdo and 3′-deoxyAdo derivatives had much higher *K*_m_ values of 575 µM and 254 µM than Ado itself (Miller *et al.*, 1979a). Our data measured for ZmADK2 (Table 3) show a remarkable similarity in *K*_D_ and *K*_m_ values of ~500 µM and 170 µM, respectively, for each ligand. This similarity can be attributed to preserving active site residues, particularly the aspartate residue (D17 in ZmADK2, D18 in human ADK) which binds the O2' and O3' hydroxyl groups of the ribose substrate.

Although ATP is the preferred phosphate donor for ADKs, other molecules such as GTP, ITP, and dATP have also been shown to be donors, although they are much weaker. Nevertheless, the reported *K*_m_ and affinity values are very inconsistent, ranging from low micromolar to low millimolar concentrations. For example, *K*_m_ values of 20 µM and 0.8 mM for ATP have been measured with rat ADK (Yamada *et al.*, 1980; Fisher and Newsholme, 1984). *K*_m_ values of 0.3–0.4 mM for ATP were measured with plant ADKs from lupin and Arabidopsis, which are in the range of reported intracellular ATP levels (Guranowski, 1979; Moffatt *et al.*, 2000).

Here, we used MST to determine *K*_D_ values, as high ATP concentrations inhibit the coupled reaction. Using this assay, the binding could be measured without interference from the Ado substrate. The calculated *K*_D_ value for ATP (in the presence of magnesium ions) with ZmADK2 was 2.2±0.6 mM (Table 3). The affinity for ADP was even lower (*K*_D_ of 10.3±1.2 mM), which is logical since reaction products usually have lower affinities than substrates. The millimolar affinity for ATP was confirmed by Ado, showing the second binding event with a *K*_D_ value of ~1.5 mM (Table 3; Supplementary Fig. S6). Indeed, Ado has been described as a competitive inhibitor of ATP binding (Fisher and Newsholme, 1984) and Ado has been observed bound to the ATP site of human ADK (Mathews *et al.*, 1998). Similarly, the AMP exhibited the first *K*_D_ value of ~250 µM, corresponding to the product binding in the Ado pocket, and the second *K*_D_ value of ~8 mM, corresponding to binding in the ATP pocket.

### Comparison of elevated ADK and NRH activities on metabolites and phenotype

NRHs and ADKs share the same Ado substrate but they catalyze opposite reactions. Specifically, ADKs add a phosphate group to Ado to produce AMP, whereas NRHs hydrolyze Ado into adenine, which can be directly converted to AMP by APTs instead of the two-step process involving PNPs followed by ADKs (Fig. 1). AMP can either be utilized by protein kinases for ATP synthesis, participate in purine interconversion reactions, or enter the purine degradation pathway. In the degradation pathway, AMP undergoes irreversible hydrolysis to IMP, which is further degraded to hypoxanthine monophosphate and then to xanthosine. The next reaction catalyzed by NRHs transforms xanthosine into xanthine, which with other downstream metabolites acts as a nitrogen source (Melino *et al.*, 2018).

Under nitrogen-limiting conditions, in *pOpON::ZmNRH* transgenic plants with elevated NRH activity, we recently highlighted accelerated purine and pyrimidine catabolism. Except for Ado, levels of uridine, xanthosine, inosine, guanosine, and iPR were significantly decreased, leading to the accumulation of nitrogen-rich amino acids such as Gln, Asn, Arg, citrulline, and agmatine, as well as polyamines (Ľuptáková *et al.*, 2024). In this study, we focused on the relevance of elevated ADK activity for purine catabolism. In inducible *pOpON::ZmADK* transgenic plants, we observed a significant decrease in ribosides such as Ado and cytokinin ribosides along with higher levels of nitrogen-rich amino acids Arg and Gln. Thus, *ADK* overexpression activates the purine degradation pathway.

From a phenotypic point of view, nitrogen deficiency can cause chlorophyll bleaching and growth retardation. Overexpression of ureide permease, another enzyme in the purine degradation pathway, has been shown to increase chlorophyll content, tiller number, and shoot and root biomass in nitrogen-starved rice (Redillas *et al.*, 2019). In *ZmNRH2b/3* overexpressor lines, root elongation was ~50% faster under nitrogen starvation than in the WT; the rosette area and green leaf index were larger (Ľuptáková *et al.*, 2024). In nitrogen-starved *ZmADK* overexpressor lines, we observed a statistically significant increase in the number of lateral roots and a larger root area, showing benefits similar to those seen in *ZmNRH* overexpressors. However, no phenotypic effects were observed in the shoot regarding plant size, rosette area, or seed number. The milder phenotypic effects of *ADK* overexpression compared with that of *NRH* behind the same inducible promotor is likely to be due to the fact that the ADK reaction product, AMP, can be diverted to other interconversion reactions before its further conversion to xanthosine, as shown in Fig. 1.

### The connection between ADK and SnRK activities in Arabidopsis

It is well known that ADK activity maintains the rate of SAM-dependent transmethylation reactions in plants by reducing free Ado levels (Moffatt *et al.*, 2002). ADK activity has been observed to increase by up to 2.8-fold in response to methyl demand (Weretilnyk *et al.*, 2001). Insufficient ADK activity has been reported to inhibit root and stem elongation and cause developmental defects in stamens and siliques, as observed in *ADK*-silenced Arabidopsis lines (Moffatt *et al.*, 2002). The effect has been attributed to the inhibition of the SAM cycle and reduced methylation reactions, because Ado must be steadily removed by ADK to prevent the inhibition of SAHH, which is part of the SAM cycle (Fig. 1).

In Arabidopsis, AtADK2 (AT5G03300) is known to form a protein complex both *in vivo* and *in vitro* with the sucrose non-fermenting 1 (SNF1)-related kinase abbreviated as SnRK1 (AT3G29160, Mohannath *et al.*, 2014). AMP prevents the dephosphorylation and thus the inactivation of SnRK1. When various stresses deplete energy stored in ATP, increased levels of AMP help to maintain SnRK1 activity, which turns off energy-consuming biosynthetic pathways and turns on alternative ATP-generating reactions (Halford and Hey, 2009; van Leene *et al.*, 2022). AtADK2 is also known to be inhibited by the geminivirus AL2 and L2 proteins, which interact with and inactivate SnRK1 kinase (Wang *et al.*, 2003). We can suggest that one of the AtADK2-binding sites is partially blocked or the rotation of a small domain is affected.

SnRK1 kinases function *in vivo* as heterotrimeric complexes composed of an α catalytic subunit containing the kinase domain (KD), and γ and β subunits. The N-terminal kinase domain of the α subunit of AtSnRK1 and its inactive K49R variant have been shown to form a complex with AtADK2, increasing ADK activity by up to 7-fold (Mohannath *et al.*, 2014). It has been hypothesized that the AtADK2/AtSnRK1-KD complex may facilitate cellular stress responses, with AtADK2 potentially providing AMP to maintain SnRK1 activity. Simultaneously, it has been suggested that the direct interaction with SnRK1 may induce conformational changes in AtADK2, leading to increased AtADK2 activity. Although the oligomeric state of AtADK2 has never been reported so far, we can speculate that the KD of AtSnRK1 might prevent AtADK2 dimerization by forming the AtADK2/AtSnRK1-KD complex. Our preliminary results with 6×His-AtADK2 expressed in *E. coli* indicate that this enzyme exists as both a monomer and a dimer at 1.2 mg ml^–1^, as evidenced by gel permeation chromatography (Supplementary Fig. S10). DLS data further reveal that AtADK2 is monomeric at lower concentrations (0.5 mg ml^–1^) and dimeric at higher concentrations (5 mg ml^–1^). An AlphaFold3 prediction model, constructed using the sequences AT3G29160 and AT5G03300 along with one ATP and two AMP molecules, shows a convincing AtADK2/AtSnRK1-KD heterodimer structure (Supplementary Fig. S10).

### A proposed model for ADK oligomerization in plants

**Fig. 7.**
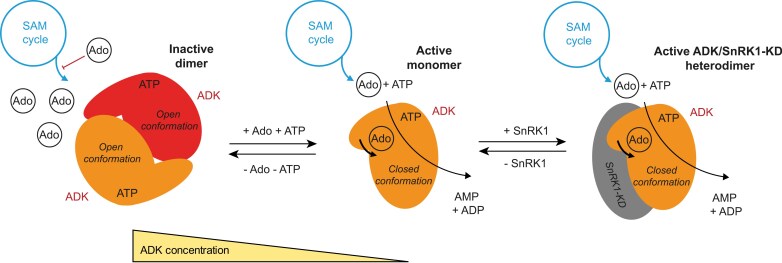
A proposed model for regulation of ADK activity in plants. Plant ADKs can associate from an active monomer to an inactive dimer in a concentration-dependent manner. The ADK dimer (left panel) adopts an open conformation. Accumulation of Ado and ATP shifts the equilibrium toward the active monomer, which can adopt a closed conformation needed for the activity (middle panel). The ADK monomer can associate with the kinase domain of SnRK1 to form the ADK–SnRK1 heterodimer (right panel), which is highly active. SnRK1 may modulate the activity of the *S*-adenosyl-l-methionine (SAM) cycle by promoting the monomeric active state of ADK through protein–protein interactions. Ado accumulation inhibits the SAHH enzyme, a key component of the SAM cycle.

Based on this work, we hypothesize that the amount of active ADK (monomer) can be significantly reduced due to the formation of inactive ADK dimers in a concentration-dependent manner. The dimerization concentration varies among species, with PpADKs dimerizing at a low concentration from 0.01 mg ml^–1^, ZmADKs at 10 mg ml^–1^ (crystallization conditions), and AtADK2 at ~1 mg ml^–1^. However, the equilibrium can shift back towards the monomeric form upon both Ado and ATP accumulation or the formation of the ADK–SnRK1 complex, which is highly active (Mohannath *et al.*, 2014). Thus, ADK activity can dynamically change based on the availability of SnRK1 during energy-demanding processes, which are known to be regulated by SnRK1 family protein kinases (Halford and Hey, 2009; van Leene *et al.*, 2022). In this way, SnRK1 could also modulate the activity of the SAM cycle via protein–protein interactions by keeping ADK monomeric (Fig. 7). Further experiments are needed to verify this hypothesis. So far, there is only evidence that SAM can regulate the AMPK pathway in mammalian hepatocytes (Martínez-Chantar *et al.*, 2006). Since there are four *SnRK1* genes in the maize genome (Zm00001d010811, Zm00001d038745, Zm00001d005108, and Zm00001d028733), two in moss (Pp6c1_19700 and Pp6c2_3600), and two in Arabidopsis (AT3G01090 and AT3G29160), it remains to be determined whether each SnRK1 can bind/activate all ADKs within a given species or if these interactions are more specific and selective.

## Supplementary data

The following supplementary data are available at *JXB* online.

Table S1. Primer pairs used for *ADK* cloning.

Table S2. Data collection and refinement statistics of maize ADKs.

Table S3. Primer pairs and probes used for RT–qPCR determination.

Table S4. Results of docking calculations into both sites in selected ADK isoforms.

Table S5. Transcript abundance of three *ADK* genes in maize tissues.

Table S6. Transcript abundance of three *ADK* genes in moss.

Table S7. Nucleoside levels in *A. thaliana ZmADK* overexpressors.

Table S8. Total root area of *pOpOn::ZmADK* transgenic lines in nitrogen-varying conditions.

Table S9. Change of leaf area in *pOpOn::ZmADK* transgenic lines in nitrogen-varying conditions.

Table S10. Levels change among selected amino acids in *pOpOn::ZmADK* transgenic lines in the absence of nitrogen.

Fig. S1. Thermal stability, molecular and kinetic properties focused on ZmADK2.

Fig. S2. Reaction scheme for the synthesis of two purine ribosides.

Fig. S3. NMR spectra of *N*^6^-methylAdo.

Fig. S4. NMR spectra of *N*^6^,*N*^6^-dimethylAdo.

Fig. S5. NMR spectra of *N*^6^-isopropylAdo.

Fig. S6. NMR spectra of *N*^6^-isobutylAdo.

Fig. S7. Sequence alignment of the selected plant ADKs and human ADK.

Fig. S8. *In silico* docking of cytokinin riboside in the active site of ADK.

Fig. S9. Comparison between the two known ADK dimers.

Fig. S10. Oligomeric state of ADK2 from *Arabidopsis thaliana*.

eraf094_suppl_Supplementary_Tables_S1-S9_Figures_S1-S10

## Data Availability

The atomic coordinates and structure factors have been deposited in the Protein Data Bank (www.wwpdb.org) under accession codes 8RF7 for ZmADK2 apoenzyme, 8RGJ for the ZmADK2 complex with AMP-PCP, 8RPA for the ZmADK3 complex with AP5A and 9FW6 for the PpADK1 complex with adenosine and ADP. Oligomeric state data and ligand binding data are deposited in the Dryad repository at https://doi.org/10.5061/dryad.qrfj6q5sj (Kopečný *et al.*, 2025). Other data are available in the Supplementary data.

## Abbreviations:

ADK: adenosine kinase
Ado: adenosine
AP5A: diadenosine pentaphosphate
APT: adenine phosphoribosyltransferase
BAPR: 6-benzylaminopurine riboside
*c*Z: *cis*-zeatin
*c*ZR: *cis*-zeatin riboside
*c*ZRMP: *cis*-zeatin riboside 5'-monophosphate
DLS: dynamic light scattering
iP: *N* ^6^-isopentenyl adenine
iPR: *N* ^6^-isopentenyl adenosine
iPRMP: *N* ^6^-isopentenyl adenosine 5'-monophosphate
LDH: lactate dehydrogenase
LOG: LONELY GUY phosphoribohydrolase
MST: microscale thermophoresis
NRH: nucleoside N-ribohydrolase
PK: pyruvate kinase
PNP: purine nucleoside phosphorylase
SAM: *S*-adenosyl-l-methionine
SnRK1: sucrose non-fermenting 1 (SNF1)-related kinase 1
*t*Z: *trans*-zeatin
*t*ZR: *trans*-zeatin riboside
WT: wild type

## References

[CIT0001] Allen M , Qin W, Moreau F, Moffatt B. 2002. Adenine phosphoribosyltransferase isoforms of Arabidopsis and their potential contributions to adenine and cytokinin metabolism. Physiologia Plantarum 115, 56–68. 12010467 10.1034/j.1399-3054.2002.1150106.x

[CIT0002] Andres CM , Fox IH. 1979. Purification and properties of human placental adenosine kinase. Journal of Biological Chemistry 254, 11388–11393. 227870

[CIT0003] Ashihara H , Stasolla C, Fujimura T, Crozier A. 2018. Purine salvage in plants. Phytochemistry 147, 89–124. 29306799 10.1016/j.phytochem.2017.12.008

[CIT0004] Baccolini C , Witte CP. 2019. AMP and GMP catabolism in Arabidopsis converge on xanthosine, which is degraded by a nucleoside hydrolase heterocomplex. The Plant Cell 31, 734–751. 30787180 10.1105/tpc.18.00899 PMC6482636

[CIT0005] Bauer MR , Mackey MD. 2019. Electrostatic complementarity as a fast and effective tool to optimize binding and selectivity of protein–ligand complexes. Journal of Medicinal Chemistry 62, 3036–3050. 30807144 10.1021/acs.jmedchem.8b01925

[CIT0006] Blondin C , Serina L, Wiesmüller L, Gilles AM, Bârzu O. 1994. Improved spectrophotometric assay of nucleoside monophosphate kinase activity using the pyruvate kinase/lactate dehydrogenase coupling system. Analytical Biochemistry 220, 219–221. 7978251 10.1006/abio.1994.1326

[CIT0007] Brautigam CA. 2015. Calculations and publication—quality illustrations for analytical ultracentrifugation data. Methods in Enzymology 562, 109–133. 26412649 10.1016/bs.mie.2015.05.001

[CIT0008] Bricogne G , Blanc E, Brandl M, et al 2011. BUSTER version 2.1.0. Cambridge: Global Phasing Ltd.

[CIT0009] Bromley JR , Warnes BJ, Newell CA, Thomson JC, James CM, Turnbull CG, Hanke DE. 2014. A purine nucleoside phosphorylase in *Solanum tuberosum* L. (potato) with specificity for cytokinins contributes to the duration of tuber endodormancy. The Biochemical Journal 458, 225–237. 24325449 10.1042/BJ20130792

[CIT0010] Burnstock G. 2007. Purine and pyrimidine receptors. Cellular and Molecular Life Sciences 64, 1471–1483. 17375261 10.1007/s00018-007-6497-0 PMC11149472

[CIT0011] Cheeseright T , Mackey M, Rose S, Vinter A. 2006. Molecular field extrema as descriptors of biological activity: definition and validation. Journal of Chemical Information and Modeling 46, 665–676. 16562997 10.1021/ci050357s

[CIT0012] Chen CM , Eckert RL. 1977. Phosphorylation of cytokinin by adenosine kinase from wheat germ. Plant Physiology 59, 443–447. 16659870 10.1104/pp.59.3.443 PMC542421

[CIT0013] Chen VB , Arendall WB 3rd, Headd JJ, Keedy DA, Immormino RM, Kapral GJ, Murray LW, Richardson JS, Richardson DC. 2010. MolProbity: all-atom structure validation for macromolecular crystallography. Acta Crystallographica. Section D, Biological Crystallography 66, 12–21. 20057044 10.1107/S0907444909042073 PMC2803126

[CIT0014] Choi J , Tanaka K, Cao Y, Qi Y, Qiu J, Liang Y, Lee SY, Stacey G. 2014. Identification of a plant receptor for extracellular ATP. Science 343, 290–294. 24436418 10.1126/science.343.6168.290

[CIT0015] Cornelius S , Traub M, Bernard C, Salzig C, Lang P, Möhlmann T. 2012. Nucleoside transport across the plasma membrane mediated by equilibrative nucleoside transporter 3 influences metabolism of Arabidopsis seedlings. Plant Biology 14, 696–705. 22372734 10.1111/j.1438-8677.2012.00562.x

[CIT0016] Darling JA , Sullivan WJ Jr, Carter D, Ullman B, Roos DS. 1999. Recombinant expression, purification, and characterization of *Toxoplasma gondii* adenosine kinase. Molecular and Biochemical Parasitology 103, 15–23. 10514077 10.1016/s0166-6851(99)00109-7

[CIT0017] Emsley P , Cowtan K. 2004. Coot: model-building tools for molecular graphics. Acta Crystallographica. Section D, Biological Crystallography 60, 2126–2132. 15572765 10.1107/S0907444904019158

[CIT0018] Fisher MN , Newsholme EA. 1984. Properties of rat heart adenosine kinase. The Biochemical Journal 221, 521–528. 6089741 10.1042/bj2210521 PMC1144068

[CIT0019] Girke C , Daumann M, Niopek-Witz S, Möhlmann T. 2014. Nucleobase and nucleoside transport and integration into plant metabolism. Frontiers in Plant Science 5, 443. 25250038 10.3389/fpls.2014.00443 PMC4158802

[CIT0020] Guranowski A. 1979. Plant adenosine kinase: purification and some properties of the enzyme from *Lupinus luteus* seeds. Archives of Biochemistry and Biophysics 196, 220–226. 41481 10.1016/0003-9861(79)90569-1

[CIT0021] Halford NG , Hey SJ. 2009. Snf1-related protein kinases (SnRKs) act within an intricate network that links metabolic and stress signalling in plants. The Biochemical Journal 419, 247–259. 19309312 10.1042/BJ20082408

[CIT0022] Han BW , Bingman CA, Mahnke DK, Bannen RM, Bednarek SY, Sabina RL, Phillips GN Jr. 2006. Membrane association, mechanism of action, and structure of Arabidopsis embryonic factor 1 (FAC1). Journal of Biological Chemistry 281, 14939–14947. 16543243 10.1074/jbc.M513009200

[CIT0023] Hatch MD. 1966. Adenylosuccinate synthetase and adenylosuccinate lyase from plant tissues. The Biochemical Journal 98, 198–203. 5938642 10.1042/bj0980198 PMC1264815

[CIT0024] Hellsten Y , Maclean D, Rådegran G, Saltin B, Bangsbo J. 1998. Adenosine concentrations in the interstitium of resting and contracting human skeletal muscle. Circulation 98, 6–8. 9665052 10.1161/01.cir.98.1.6

[CIT0025] Hirose N , Takei K, Kuroha T, Kamada-Nobusada T, Hayashi H, Sakakibara H. 2008. Regulation of cytokinin biosynthesis, compartmentalization and translocation. Journal of Experimental Botany 59, 75–83. 17872922 10.1093/jxb/erm157

[CIT0026] Iltzsch MH , Uber SS, Tankersley KO, el Kouni MH. 1995. Structure–activity relationship for the binding of nucleoside ligands to adenosine kinase from *Toxoplasma gondii*. Biochemical Pharmacology 49, 1501–1512. 7763293 10.1016/0006-2952(95)00029-y

[CIT0027] Janin J , Rodier F, Chakrabarti P, Bahadur RP. 2007. Macromolecular recognition in the Protein Data Bank. Acta Crystallographica. Section D, Biological Crystallography 63, 1–8. 17164520 10.1107/S090744490603575X PMC2483476

[CIT0028] Jung B , Flörchinger M, Kunz HH, Traub M, Wartenberg R, Jeblick W, Neuhaus HE, Möhlmann T. 2009. Uridine-ribohydrolase is a key regulator in the uridine degradation pathway of Arabidopsis. The Plant Cell 21, 876–891. 19293370 10.1105/tpc.108.062612 PMC2671717

[CIT0029] Jung B , Hoffmann C, Möhlmann T. 2011. Arabidopsis nucleoside hydrolases involved in intracellular and extracellular degradation of purines. The Plant Journal 65, 703–711. 21235647 10.1111/j.1365-313X.2010.04455.x

[CIT0030] Kabsch W. 2010. XDS. Acta Crystallographica. Section D, Biological Crystallography 66, 125–132. 20124692 10.1107/S0907444909047337 PMC2815665

[CIT0031] Karlíková R , Široká J, Friedecký D, et al 2016. Metabolite profiling of the plasma and leukocytes of chronic myeloid leukemia patients. Journal of Proteome Research 15, 3158–3166. 27465658 10.1021/acs.jproteome.6b00356

[CIT0032] Karplus PA , Diederichs K. 2012. Linking crystallographic model and data quality. Science 336, 1030–1033. 22628654 10.1126/science.1218231 PMC3457925

[CIT0033] Khakh BS , Burnstock G. 2009. The double life of ATP. Scientific American 301, 84–90, 92. 10.1038/scientificamerican1209-84 PMC2877495 20058644

[CIT0034] Končitíková R , Vigouroux A, Kopečná M, Andree T, Bartoš J, Šebela M, Moréra S, Kopečný D. 2015. Role and structural characterization of plant aldehyde dehydrogenases from family 2 and family 7. The Biochemical Journal 468, 109–123. 25734422 10.1042/BJ20150009

[CIT0035] Kopečná M , Blaschke H, Kopečný D, Vigouroux A, Končitíková R, Novák O, Kotland O, Strnad M, Moréra S, von Schwartzenberg K. 2013. Structure and function of nucleoside hydrolases from *Physcomitrella patens* and maize catalyzing the hydrolysis of purine, pyrimidine, and cytokinin ribosides. Plant Physiology 163, 1568–1583. 24170203 10.1104/pp.113.228775 PMC3850210

[CIT0036] Kopečný DJ , Vigouroux A, Bělíček A, et al 2025. Data from: A monomer–dimer switch modulates the activity of plant adenosine kinase. Dryad Digital Repository 10.5061/dryad.qrfj6q5sj PMC12369479 40063605

[CIT0037] Kraft C , Deplazes A, Sohrmann M, Peter M. 2008. Mature ribosomes are selectively degraded upon starvation by an autophagy pathway requiring the Ubp3p/Bre5p ubiquitin protease. Nature Cell Biology 10, 602–610. 18391941 10.1038/ncb1723

[CIT0038] Kuettel S , Greenwald J, Kostrewa D, Ahmed S, Scapozza L, Perozzo R. 2011. Crystal structures of *T. b. rhodesiense* adenosine kinase complexed with inhibitor and activator: implications for catalysis and hyperactivation. PLoS Neglected Tropical Diseases 5, e1164. 21629723 10.1371/journal.pntd.0001164 PMC3101181

[CIT0039] Kuhn M , Firth-Clark S, Tosco P, Mey ASJS, Mackey M, Michel J. 2020. Assessment of binding affinity via alchemical free-energy calculations. Journal of Chemical Information and Modeling 60, 3120–3130. 32437145 10.1021/acs.jcim.0c00165

[CIT0040] Kuroha T , Tokunaga H, Kojima M, Ueda N, Ishida T, Nagawa S, Fukuda H, Sugimoto K, Sakakibara H. 2009. Functional analyses of *LONELY GUY* cytokinin-activating enzymes reveal the importance of the direct activation pathway in Arabidopsis. The Plant Cell 21, 3152–3169. 19837870 10.1105/tpc.109.068676 PMC2782294

[CIT0041] Kwade Z , Swiatek A, Azmi A, Goossens A, Inzé D, Van Onckelen H, Roef L. 2005. Identification of four adenosine kinase isoforms in tobacco By-2 cells and their putative role in the cell cycle-regulated cytokinin metabolism. Journal of Biological Chemistry 280, 17512–17519. 15731114 10.1074/jbc.M411428200

[CIT0042] Long MC , Escuyer V, Parker WB. 2003. Identification and characterization of a unique adenosine kinase from *Mycobacterium tuberculosis*. Journal of Bacteriology 185, 6548–6555. 14594827 10.1128/JB.185.22.6548-6555.2003 PMC262096

[CIT0043] Long MC , Parker WB. 2006. Structure–activity relationship for nucleoside analogs as inhibitors or substrates of adenosine kinase from *Mycobacterium tuberculosis*. I. Modifications to the adenine moiety. Biochemical Pharmacology 71, 1671–1682. 16620788 10.1016/j.bcp.2006.03.006

[CIT0044] Ľuptáková E , Vigouroux A, Končitíková R, et al 2024. Plant nucleoside N-ribohydrolases: riboside binding and role in nitrogen storage mobilization. The Plant Journal 117, 1432–1452. 38044809 10.1111/tpj.16572

[CIT0045] Manalastas-Cantos K , Konarev PV, Hajizadeh NR, et al 2021. *ATSAS 3.0*: expanded functionality and new tools for small-angle scattering data analysis. Journal of Applied Crystallography 54, 343–355. 33833657 10.1107/S1600576720013412 PMC7941305

[CIT0046] Martínez-Chantar ML , Vázquez-Chantada M, Garnacho M, et al 2006. S-Adenosylmethionine regulates cytoplasmic HuR via AMP-activated kinase. Gastroenterology 131, 223–232. 16831604 10.1053/j.gastro.2006.04.019

[CIT0047] Mathews II , Erion MD, Ealick SE. 1998. Structure of human adenosine kinase at 1.5 Å resolution. Biochemistry 37, 15607–15620. 9843365 10.1021/bi9815445

[CIT0048] Melino VJ , Casartelli A, George J, Rupasinghe T, Roessner U, Okamoto M, Heuer S. 2018. RNA catabolites contribute to the nitrogen pool and support growth recovery of wheat. Frontiers in Plant Science 9, 1539. 30455708 10.3389/fpls.2018.01539 PMC6230992

[CIT0049] Miller RL , Adamczyk DL, Miller WH. 1979b. Adenosine kinase from rabbit liver. I. Purification by affinity chromatography and properties. Journal of Biological Chemistry 254, 2339–2345. 218933

[CIT0050] Miller RL , Adamczyk DL, Miller WH, Koszalka GW, Rideout JL, Beacham LM 3rd, Chao EY, Haggerty JJ, Krenitsky TA, Elion GB. 1979a. Adenosine kinase from rabbit liver. II. Substrate and inhibitor specificity. Journal of Biological Chemistry 254, 2346–2352. 218934

[CIT0051] Moffatt B , Pethe C, Laloue M. 1991. Metabolism of benzyladenine is impaired in a mutant of *Arabidopsis thaliana* lacking adenine phosphoribosyltransferase activity. Plant Physiology 95, 900–908. 16668070 10.1104/pp.95.3.900 PMC1077622

[CIT0052] Moffatt B , Somerville C. 1988. Positive selection for male-sterile mutants of Arabidopsis lacking adenine phosphoribosyl transferase activity. Plant Physiology 86, 1150–1154. 16666047 10.1104/pp.86.4.1150 PMC1054643

[CIT0053] Moffatt BA , Stevens YY, Allen MS, Snider JD, Pereira LA, Todorova MI, Summers PS, Weretilnyk EA, Martin-McCaffrey L, Wagner C. 2002. Adenosine kinase deficiency is associated with developmental abnormalities and reduced transmethylation. Plant Physiology 128, 812–821. 11891238 10.1104/pp.010880 PMC152195

[CIT0054] Moffatt BA , Wang L, Allen MS, Stevens YY, Qin W, Snider J, von Schwartzenberg K. 2000. Adenosine kinase of Arabidopsis. Kinetic properties and gene expression. Plant Physiology 124, 1775–1785. 11115893 10.1104/pp.124.4.1775 PMC59874

[CIT0055] Mohannath G , Jackel JN, Lee YH, Buchmann RC, Wang H, Patil V, Adams AK, Bisaro DM. 2014. A complex containing SNF1-related kinase (SnRK1) and adenosine kinase in Arabidopsis. PLoS One 9, e87592. 24498147 10.1371/journal.pone.0087592 PMC3907550

[CIT0056] Mok DWS , Mok MC. 2001. Cytokinin metabolism and action. Annual Review of Plant Physiology and Plant Molecular Biology 52, 89–118. 10.1146/annurev.arplant.52.1.89 11337393

[CIT0057] Muchmore SW , Smith RA, Stewart AO, et al 2006. Crystal structures of human adenosine kinase inhibitor complexes reveal two distinct binding modes. Journal of Medicinal Chemistry 49, 6726–6731. 17154503 10.1021/jm060189a

[CIT0058] Pfefferkorn ER , Pfefferkorn LC. 1976. Arabinosyl nucleosides inhibit *Toxoplasma gondii* and allow the selection of resistant mutants. Journal of Parasitology 62, 993–999. 1003290

[CIT0059] Plačková L , Okleštková J, Pospíšková K, et al 2017. Microscale magnetic microparticle-based immunopurification of cytokinins from Arabidopsis root apex. The Plant Journal 89, 1065–1075. 27943492 10.1111/tpj.13443

[CIT0060] Reddy MCM , Palaninathan SK, Shetty ND, Owen JL, Watson MD, Sacchettini JC. 2007. High resolution crystal structures of *Mycobacterium tuberculosis* adenosine kinase: insights into the mechanism and specificity of this novel prokaryotic enzyme. Journal of Biological Chemistry 282, 27334–27342. 17597075 10.1074/jbc.M703290200

[CIT0061] Redillas MCFR , Bang SW, Lee DK, Kim YS, Jung H, Chung PJ, Suh JW, Kim JK. 2019. Allantoin accumulation through overexpression of ureide permease1 improves rice growth under limited nitrogen conditions. Plant Biotechnology Journal 17, 1289–1301. 30565833 10.1111/pbi.13054 PMC6577366

[CIT0062] Romanello L , Bachega JF, Cassago A, Brandão-Neto J, DeMarco R, Garratt RC, Pereira HD. 2013. Adenosine kinase from *Schistosoma mansoni*: structural basis for the differential incorporation of nucleoside analogues. Acta Crystallographica. Section D, Biological Crystallography 69, 126–136. 23275171 10.1107/S0907444912044800

[CIT0063] Sakakibara H. 2006. Cytokinins: activity, biosynthesis, and translocation. Annual Review of Plant Biology 57, 431–449. 10.1146/annurev.arplant.57.032905.105231 16669769

[CIT0064] Sauter M , Moffatt B, Saechao MC, Hell R, Wirtz M. 2013. Methionine salvage and S-adenosylmethionine: essential links between sulfur, ethylene and polyamine biosynthesis. The Biochemical Journal 451, 145–154. 23535167 10.1042/BJ20121744

[CIT0065] Schindelin J , Arganda-Carreras I, Frise E, et al 2012. Fiji: an open-source platform for biological-image analysis. Nature Methods 9, 676–682. 22743772 10.1038/nmeth.2019 PMC3855844

[CIT0066] Schmülling T , Werner T, Riefler M, Krupková E, Bartrina y Manns I. 2003. Structure and function of cytokinin oxidase/dehydrogenase genes of maize, rice, Arabidopsis and other species. Journal of Plant Research 116, 241–252. 12721786 10.1007/s10265-003-0096-4

[CIT0067] Schoor S , Farrow S, Blaschke H, Lee S, Perry G, von Schwartzenberg K, Emery N, Moffatt B. 2011. Adenosine kinase contributes to cytokinin interconversion in Arabidopsis. Plant Physiology 157, 659–672. 21803861 10.1104/pp.111.181560 PMC3192563

[CIT0068] Schuck P. 2000. Size-distribution analysis of macromolecules by sedimentation velocity ultracentrifugation and lamm equation modeling. Biophysical Journal 78, 1606–1619. 10692345 10.1016/S0006-3495(00)76713-0 PMC1300758

[CIT0069] Schumacher MA , Scott DM, Mathews II, Ealick SE, Roos DS, Ullman B, Brennan RG. 2000. Crystal structures of *Toxoplasma gondii* adenosine kinase reveal a novel catalytic mechanism and prodrug binding. Journal of Molecular Biology 298, 875–893. 10801355 10.1006/jmbi.2000.3753

[CIT0070] Schwartzman JD , Pfefferkorn ER. 1982. *Toxoplasma gondii*: purine synthesis and salvage in mutant host cells and parasites. Experimental Parasitology 53, 77–86. 7198995 10.1016/0014-4894(82)90094-7

[CIT0071] Storoni LC , McCoy AJ, Read RJ. 2004. Likelihood-enhanced fast rotation functions. Acta Crystallographica. Section D, Biological Crystallography 60, 432–438. 14993666 10.1107/S0907444903028956

[CIT0072] Stroganov OV , Novikov FN, Stroylov VS, Kulkov V, Chilov GG. 2008. Lead finder: an approach to improve accuracy of protein–ligand docking, binding energy estimation, and virtual screening. Journal of Chemical Information and Modeling 48, 2371–2385. 19007114 10.1021/ci800166p

[CIT0073] Supíková K , Kosinová A, Vavruša M, Kopliková L, François A, Pospíšil J, Zatloukal M, Wever R, Hartog A, Grúz J. 2022. Sulfated phenolic acids in plants. Planta 255, 124. 35562552 10.1007/s00425-022-03902-6

[CIT0074] Svačinová J , Novák O, Plačková L, Lenobel R, Holík J, Strnad M, Doležal K. 2012. A new approach for cytokinin isolation from Arabidopsis tissues using miniaturized purification: pipette tip solid-phase extraction. Plant Methods 8, 17. 22594941 10.1186/1746-4811-8-17 PMC3492005

[CIT0075] Tickle IJ , Flensburg C, Keller P, Paciorek W, Sharff A, Vonrhein C, Bricogne G. 2016. STARANISO. Cambridge: Global Phasing Ltd, http://staraniso.globalphasing.org/cgi-bin/staraniso.cgi

[CIT0076] Timm J , González-Pacanowska D, Wilson KS. 2014. Structures of adenosine kinase from *Trypanosoma brucei brucei*. Acta Crystallographica. Section F, Structural Biology Communications 70, 34–39. 24419613 10.1107/S2053230X13033621 PMC3943091

[CIT0077] Tranová L , Buček J, Zatloukal M, Cankař P, Stýskala J. 2019. Synthesis of [^15^ N_4_]purine labeled cytokinin glycosides derived from zeatins and topolins with 9-β-d, 7-β-d-glucopyranosyl, or 9-β-d-ribofuranosyl group. Journal of Labelled Compounds & Radiopharmaceuticals 62, 118–125. 10.1002/jlcr.3702 30592529

[CIT0078] van Leene J , Eeckhout D, Gadeyne A, et al 2022. Mapping of the plant SnRK1 kinase signalling network reveals a key regulatory role for the class II T6P synthase-like proteins. Nature Plants 8, 1245–1261. 36376753 10.1038/s41477-022-01269-w

[CIT0079] Vodnala M , Fijolek A, Rofougaran R, Mosimann M, Mäser P, Hofer A. 2008. Adenosine kinase mediates high affinity adenosine salvage in *Trypanosoma brucei*. Journal of Biological Chemistry 283, 5380–5388. 18167353 10.1074/jbc.M705603200

[CIT0080] von Schwartzenberg K , Kruse S, Reski R, Moffatt B, Laloue M. 1998. Cloning and characterization of an adenosine kinase from *Physcomitrella* involved in cytokinin metabolism. The Plant Journal 13, 249–257. 9680981 10.1046/j.1365-313x.1998.00011.x

[CIT0081] Wang H , Hao L, Shung CY, Sunter G, Bisaro DM. 2003. Adenosine kinase is inactivated by geminivirus AL2 and L2 proteins. The Plant Cell 15, 3020–3032. 14615595 10.1105/tpc.015180 PMC282852

[CIT0082] Weretilnyk EA , Alexander KJ, Drebenstedt M, Snider JD, Summers PS, Moffatt BA. 2001. Maintaining methylation activities during salt stress. The involvement of adenosine kinase. Plant Physiology 125, 856–865. 11161043 10.1104/pp.125.2.856 PMC64887

[CIT0083] Wielopolska A , Townley H, Moore I, Waterhouse P, Helliwell C. 2005. A high-throughput inducible RNAi vector for plants. Plant Biotechnology Journal 3, 583–590. 17147629 10.1111/j.1467-7652.2005.00149.x

[CIT0084] Wormit A , Traub M, Flörchinger M, Neuhaus HE, Möhlmann T. 2004. Characterization of three novel members of the *Arabidopsis thaliana* equilibrative nucleoside transporter (ENT) family. The Biochemical Journal 383, 19–26. 15228386 10.1042/BJ20040389 PMC1134039

[CIT0085] Xu J , Zhang HY, Xie CH, Xue HW, Dijkhuis P, Liu CM. 2005. *EMBRYONIC FACTOR 1* encodes an AMP deaminase and is essential for the zygote to embryo transition in Arabidopsis. The Plant Journal 42, 743–756. 15918887 10.1111/j.1365-313X.2005.02411.x

[CIT0086] Yamada Y , Goto H, Ogasawara N. 1980. Purification and properties of adenosine kinase from rat brain. Biochimica et Biophysica Acta 616, 199–207. 6260151 10.1016/0005-2744(80)90138-2

[CIT0087] Zhang Y , El Kouni MH, Ealick SE. 2007. Substrate analogs induce an intermediate conformational change in *Toxoplasma gondii* adenosine kinase. Acta Crystallographica. Section D, Biological Crystallography 63, 126–134. 17242506 10.1107/S0907444906043654

[CIT0088] Zrenner R , Stitt M, Sonnewald U, Boldt R. 2006. Pyrimidine and purine biosynthesis and degradation in plants. Annual Review of Plant Biology 57, 805–836. 10.1146/annurev.arplant.57.032905.105421 16669783

