# Supplementary material for: A monomer–dimer switch modulates the activity of plant adenosine kinase
Source: J Exp Bot. 2025 Mar 10;76(12):3457–79. doi: 10.1093/jxb/eraf094 (PMC12369479; doi:10.1093/jxb/eraf094)

## SUPPLEMENTARY DATA

# A monomer-dimer switch modulates the activity of plant adenosine kinase

David Jaroslav Kopečný[1], Armelle Vigouroux[2], Jakub Bělíček[1], Martina Kopečná[1], Radka Končitíková[1], Jaroslava Friedecká[3], Václav Mik[1], Klára Supíková[1], Jan František Humplík[4], Marine Le Berre[2], Stephan Plancqueel[2], Miroslav Strnad[3], Klaus von Schwartzenberg[5], Ondřej Novák[3], Solange Moréra[2,*], David Kopečný[1,*]

[1]Department of Experimental Biology, Faculty of Science, Palacký University, Olomouc CZ-78371, Czech Republic
[2]Université Paris-Saclay, CEA, CNRS, Institute for Integrative Biology of the Cell (I2BC,) Gif-sur-Yvette F-91198, France
[3]Laboratory of Growth Regulators, Faculty of Science, Palacký University & Institute of Experimental Botany of the Czech Academy of Sciences, Šlechtitelů 11, Olomouc CZ-78371, Czech Republic
[4]Department of Chemical Biology, Faculty of Science, Palacký University, Olomouc CZ-78371, Czech Republic
[5]Institute for Plant Science and Microbiology, Universität Hamburg, 22609 Hamburg, Germany

### *Corresponding authors

david.kopecny@upol.cz; Tel.: +420 585634840
solange.morera@i2bc.paris-saclay.fr; Tel.: +33 1 69824213

**Supplementary Table S1.** Primer pairs used for *ADK* cloning.

**Supplementary Table S2.** Data collection and refinement statistics of maize ADKs.

**Supplementary Table S3.** Primer pairs and probes used for RT-qPCR determination.

**Supplementary Table S4.** Results of docking calculations into both sites in selected ADK isoforms.

**Supplementary Table S5.** Transcript abundance of three *ADK* genes in maize tissues.

**Supplementary Table S6.** Transcript abundance of three *ADK* genes in moss.

**Supplementary Table S7.** Nucleoside levels in *A. thaliana ZmADK* overexpressors.

**Supplementary Table S8.** Total root area of *pOpOn::ZmADK* transgenic lines in nitrogen-varying conditions.

**Supplementary Table S9.** Change of leaf area in *pOpOn::ZmADK* transgenic lines in nitrogen-varying conditions.

**Supplementary Table S10.** Levels change among selected amino acids in *pOpOn::ZmADK* transgenic lines in the absence of nitrogen.

**Supplementary Fig. S1.** Thermal stability, molecular and kinetic properties focused on ZmADK2.

**Supplementary Fig. S2.** Reaction scheme for the synthesis of two purine ribosides.

**Supplementary Fig. S3.** NMR spectra of $N^6$-methylAdo.

**Supplementary Fig. S4.** NMR spectra of $N^6$, $N^6$-dimethylAdo.

**Supplementary Fig. S5.** NMR spectra of $N^6$-isopropylAdo.

**Supplementary Fig. S6.** NMR spectra of $N^6$-isobutylAdo.

**Supplementary Fig. S7.** Sequence alignment of the selected plant ADKs and human ADK.

**Supplementary Fig. S8.** *In silico* docking of cytokinin riboside in the active site of ADK.

**Supplementary Fig. S9.** Comparison between the two known ADK dimers.

**Supplementary Fig. S10.** Oligomeric state of ADK2 from *Arabidopsis thaliana*.

**Supplementary Table S1**. **Primer pairs used for *ADK* cloning.**

| Cloned *ADK* gene | Primer pairs |
|---|---|
| *ZmADK1 and ZmADK2* | 5′- ATTGAGCTCGATGGCGGCGAGCGAGGGCGT-3′ |
| | 5′- ACTGGTACCCTAGTTGAAGTCAGGCTTCT-3′ |
| *ZmADK3* | 5′- ATTGAGCTCGGCCAGCAGCGGCTACGAAGGGA-3′ |
| | 5′- ACTGGTACCCTAGTTGAAGTCAGGCTTCT-3′ |
| *PpADK1* | 5′- ATAGAGCTCGATGGCGTCCGAGGGTGTGCTTTT-3′ |
| | 5′- ACTGGTACCCTACTGACTTTCGAAGGATGGTT-3′ |
| *PpADK2* | 5′- CATGGATCCGATGGCGTCCGAAGGAGTG-3′ |
| | 5′- CATCTCGAGCTACTCCGACTGAAAACAAG-3′ |

**Supplementary Table S2. Data collection and refinement statistics.**

| Enzyme | **ZmADK2** | **ZmADK2** | **ZmADK3** | **PpADK1** |
|---|---|---|---|---|
| Observed ligand | - | AMP-PCP | AP5A | Ado + ADP |
| PDB code | 8RF7 | 8RGJ | 8RPA | 9FW6 |
| Space group | P4212 | P4212 | I212121 | P212121 |
| Asymmetric unit | 1 dimer | 1 dimer | 1 monomer | 2 monomers |
| Unit cell (Å) | | | | |
| a | 136.22 | 135.85 | 51.0 | 50.59 |
| b | 136.22 | 135.85 | 117.5 | 93.08 |
| c | 78.23 | 78.63 | 166.0 | 131.67 |
| α (°) | 90.0 | 90.0 | 90.0 | 90.0 |
| β (°) | 90.0 | 90.0 | 90.0 | 90.0 |
| γ (°) | 90.0 | 90.0 | 90.0 | 90.0 |
| Resolution (Å) | 96.3 – 2.05 (2.17 – 2.05) | 96.1 – 2.36 (2.51 – 2.36) | 48.0 – 2.26 (2.54 – 2.26) | 76.0 – 1.73 (1.93 – 1.73) |
| Resolution limits by Staraniso (Å) | 2.05, 2.05, 3.02 | 2.36, 2.36, 3.31 | 3.11, 2.52, 2.26 | 2.48, 1.73, 2.05 |
| Observed reflections | 800280 (41504) | 535990 (28034) | 162656 (6984) | 442676 (23441) |
| Unique reflections | 30369 (1520) | 20930 (1046) | 14056 (703) | 37232 (1862) |
| Completeness (spherical) (%) | 65.2 (19.2) | 67.7 (20.8) | 58.8 (10.2) | 56.7 (10.1) |
| Completeness (ellipsoidal) (%) | 94.9 (80.4) | 93.7 (78.9) | 89.8 (68.6) | 93.1 (82.2) |
| $I/\sigma(I)$ | 15.6 (1.5) | 12.4 (1.5) | 8.5 (1.4) | 6.1 (1.7) |
| $R_{sym}$ (%) | 20.4 (287.9) | 31.4 (285.6) | 26.3 (195.5) | 31.5 (233.5) |
| $R_{pim}$ (%) | 4.0 (55.9) | 6.3 (55.9) | 7.7 (57.1) | 9.5 (67.8) |
| $CC_{1/2}$ | 99.9 (40.5) | 99.7 (51.1) | 99.4 (65.2) | 98.4 (61.0) |
| Amino acid residues | 667 | 671 | 341 | 670 |
| Water molecules | 174 | 147 | 99 | 320 |
| Ligand molecules | - | 2 | 1 | 4 |
| $R_{cryst}$ (%)[c] | 23.4 | 26.0 | 20.8 | 22.0 |
| $R_{free}$ (%) | 26.1 | 27.8 | 25.7 | 25.5 |
| RMSD bond lengths (Å) | 0.08 | 0.007 | 0.007 | 0.009 |
| RMSD bond angles (°) | 0.97 | 0.88 | 0.88 | 1.00 |
| Mean B value (Å$^2$): | | | | |
| Overall | 48.9 | 56.2 | 31.2 | 30.3 |
| protein chain A/B | 49.9/48.5 | 55.5/55.8 | 31.6 | 33.1/27.8 |
| solvent molecules | 49.6 | 54.3 | 21.4 | 25.9 |
| ligand A/B | -/- | 74.6/84.2 (AMP-PCP) | 29.2 (AP5A) | 34.4/31.4 (ADP) 21.5/17.5 (Ado) |
| Ramachandran statistics (%)[d]: | | | | |
| Favored | 99.24 | 95.92 | 97.1 | 97.90 |
| Outliers | 0.0 | 0.3 | 0.0 | 0.0 |
| Clash score (PR)[d] | 1.27 | 1.16 | 0.75 | 0.39 |
| MolProbity score (PR)[d] | 0.85 | 1.11 | 1.10 | 0.70 |

[a] Numbers in parentheses represent values in the highest resolution shell.
[b] $CC_{1/2}$ represents a percentage of correlation between intensities from a random half-dataset.
[c] The 5% test set.
[d] Generated with MolProbity.

**Supplementary Table S3. Primer pairs and probes used for RT-qPCR determination.**

| | Primer pairs and probes for RT-qPCR |
|---|---|
| *ZmADK1* | 5´- AGGTCCTCCCGTATGCTGACT -3´, |
| | 5´- CTGTCTCCCATCCTCGAACTTT -3´, |
| | 5´-FAM- CATCTTCGGCAATGAAACCGAGGC -TAM -3´ |
| *ZmADK2* | 5´- AGGTGAAAACGTTCCCTGTGA -3´, |
| | 5´- CAATGCCCTTTCCTAGAACCAA -3´, |
| | 5´-FAM- ATGCTTTCGTTGGAGGCTTCCTCTCAC -TAM -3´ |
| *ZmADK3* | 5´- TCCCCGGATTCTATTCAACTTG -3´, |
| | 5´- GGAGCAGAAAGGTTCATCATAAACA -3´, |
| | 5´-FAM- TGCTGAGCATGCCGCTGCAAC -TAM -3´ |
| *PpADK1* | 5´- CGATACGATGTGGACGAGGAT -3´, |
| | 5´- CCAAGGACCTCTCTCCCTTCA -3´, |
| | 5´-FAM- TTCCCACTGGAACATGCGGCG -TAM -3´ |
| *PpADK2* | 5´- GGCTGCGTTGCCCAAA -3´, |
| | 5´- GGGATCAGTTCCCTGAGTGATAA -3´, |
| | 5´-FAM- CTAGCGGCACTCACAAGCGTGTTGC -TAM -3´ |
| *PpADK3* | 5´- TCATTTGTTTGTGAGCGTTTTAAAG -3´, |
| | 5´- GCCTCATTCCCAAACATGTAGTC -3´, |
| | 5´-FAM- TCCTCTCATGGCAGCCTTCCCATATG -TAM -3´ |
| *AtNRH1* | 5´-TCTAGATGAGAAGGTCGAAGAATATCC-3´, |
| | 5´-GGCTAATGCCAGGTTGGTTAGAG-3´, |
| | 5´-FAM-TGAAGTCACCATTCTCGCCCTCGG-TAM-3´ |
| *AtNRH2* | 5´-CCTCACTACTATCTTTGGAAACGTGTA-3´, |
| | 5´-CGCAACCTCCAACAAATGC-3´, |
| | 5´-FAM- CCACTCTCGCCACTCGAAACGCC-TAM-3´ |
| *AtADK1* | 5´-GTCGCTGAGGATGGAAAAGTG-3´, |
| | 5´-GCACCGTTGGTGTCAACAAG-3´, |
| | 5´-FAM- AGAAGTACCCAGTCATCCCTCTCCCCAA-TAM-3´ |
| *AtADK2* | 5´-TCGAACAAATAGCCATCAAGATTTC-3´, |
| | 5´-CAGCGCCCTGTGTAATCACA-3´, |
| | 5´-FAM- CCCAAGGCCACAGGAACATACAAGAGG-TAM-3´ |
| *AtAPT1* | 5´-CTCAAACCACCGTTCAACCA-3´, |
| | 5´-CGGCGTCAGATTTGCAAAC-3´, |
| | 5´-FAM- CCTCTTCTCCTCCGCCGGGTCTC-TAM-3´ |
| *AtAPT3* | 5´-GGCTCGTGGTTTCCTATTCG-3´, |
| | 5´-GTTTGCGCAGAGGAACAAATTT3´, |
| | 5´-FAM- TCCACCGATCGCGCTAGCCATT-TAM-3´ |
| *AtLOG7* | 5´-TATGCATCAAAGGAAAGCTGAAAT-3´, |
| | 5´-AACGTACCATACCCACCAGGAA-3´, |
| | 5´-FAM- CTCGCCAAGCCGACGCATTCA-TAM-3´ |
| *AtLOG8* | 5´-CAGCGAAGCAGATTCAGAAAAA-3´, |
| | 5´-GGCAGCATCACTGAAAACTTCTC-3´, |
| | 5´-FAM- TCTTTTGCGGAAGCCACTCTGGTCA-TAM-3´ |

**Supplementary Table S4. Results of docking calculations into both sites in selected ADK isoforms.** Docked using FLARE software (www.cresset-group.com/software/flare-docking/). The rank score is designed to provide the best score for the correct (experimentally observed) ligand pose; ΔG represents an accurate estimation of the free energy of protein-ligand binding for a given protein-ligand complex.

| | **Ligand** | ***ZmADK2* *(8RGJ, opened)*** | | ***ZmADK3* *(8RPA; closed)*** | | ***PpADK1* *(9FW6; closed)*** | | ***HsADK* *(1BX4; closed)*** | |
|---|---|---|---|---|---|---|---|---|---|
| | | Rank Score | Δ*G score* | Rank Score | Δ*G* | Rank Score | Δ*G* | Rank Score | Δ*G* |
| | | | | | (kcal mol$^{-1}$) | | | | |
| *Ado-site* | *Ado* | -4.6 | -4.4 | -10.3 | -7.5 | -10.1 | -8.5 | -10.4 | -8.0 |
| | *AMP* | -4.7 | -5.0 | -9.5 | -7.8 | -10.6 | -8.6 | -7.4 | -7.2 |
| | *iPR* | -5.2 | -5.5 | -8.6 | -8.6 | -9.8 | -9.8 | -9.3 | -9.0 |
| | *BAPR* | -5.9 | -4.9 | -9.2 | -8.3 | -10.0 | -9.3 | -9.6 | -8.1 |
| *ATP-site* | *Ado* | n. d. | n. d. | -5.0 | -5.3 | -6.6 | -5.9 | -7.0 | -6.4 |
| | *AMP* | -3.9 | -4.4 | -3.2 | -4.3 | -5.7 | -5.7 | -6.4 | -5.8 |
| | *ADP* | -3.3 | -4.4 | -3.3 | -4.7 | -6.9 | -6.1 | -6.0 | -5.6 |
| | *ATP* | -3.3 | -4.3 | -3.4 | -3.6 | -7.3 | -6.2 | -5.2 | -5.5 |

**Supplementary Table S5. Transcript abundance of three *ADK* genes in maize tissues.** Abundance is the number of transcripts per ng of total RNA amplified by qPCR. RNA from four biological replicates was transcribed in two independent reactions and PCR was performed in duplicate. Mean values ± standard deviations are shown. DAG, days after germination; DAP, days after pollination; DBP, days before pollination; M, months.

| | Gene (transcripts $ng^{-1}$ of total RNA) | | |
|---|---|---|---|
| **Sample** | ***ZmADK1*** | ***ZmADK2*** | ***ZmADK3*** |
| embryo | 106.2 ± 11.3 | 1160.6 ± 129.4 | 134.9 ± 11 |
| stem (9 DAG) | 187.8 ± 14.4 | 336.1 ± 16.8 | 9.9 ± 1.2 |
| leaf (9 DAG) | 256.2 ± 26 | 277.6 ± 27.7 | 71.3 ± 4.3 |
| root (9 DAG) | 50 ± 8.3 | 293.8 ± 23.6 | 12.1 ± 2.7 |
| stem (3 M) | 172.1 ± 17.4 | 883.8 ± 78.9 | 94.1 ± 5.7 |
| leaves (3 M) | 130.3 ±10.1 | 1145.4 ± 113.2 | 141.3 ± 31 |
| main root (3 M) | 88.5 ± 4.4 | 697.7 ± 20 | 28.9 ± 2.5 |
| tassels (5 DBP) | 98.6 ± 3.6 | 731.6 ± 120.4 | 28.9 ± 2.9 |
| tassels (0 DAP) | 555 ± 51.8 | 2923.6 ± 390.7 | 227.8 ± 50 |
| silks (0 DAP) | 163.9 ± 2.2 | 572.3 ± 103.9 | 53.2 ± 2.6 |
| silks (3 DAP) | 80 ± 2.3 | 2773.8 ± 314.6 | 337.1 ± 85.7 |
| silks (6 DAP) | 109.5 ± 3.2 | 2373.4 ± 356.9 | 1003.2 ± 141.1 |
| kernel (3 DBP) | 351.5 ± 20.9 | 649.1 ± 68.8 | 121.8 ± 14.8 |
| kernel (3 DAP) | 801.5 ± 23.7 | 1263.1 ± 177 | 504.9 ± 115.7 |
| kernel (9 DAP) | 1353.1 ± 417.1 | 1345 ± 157.5 | 313.4 ± 48.9 |
| kernel (15 DAP) | 7634.4 ± 425.3 | 10611 ± 1975.6 | 2371.2 ± 139.7 |
| kernel (20 DAP) | 1869.5 ± 163.5 | 3522.5 ± 514.4 | 1636.3 ± 150.4 |
| ***Stem*** | | | |
| 3 DAG | 140.5 ± 10.5 | 304.6 ± 30.2 | 10.2 ± 0.7 |
| 5 DAG | 94.7 ± 4.9 | 243.4 ± 13.9 | 5.7 ± 1.6 |
| 7 DAG | 75.1 ± 10.3 | 215.5 ± 33.0 | 3.8 ± 0.9 |
| 9 DAG | 205.9 ± 34.4 | 385.1 ± 35.1 | 5.7 ± 0.5 |
| 11 DAG | 187.8 ± 16.8 | 336.1 ± 58.1 | 9.9 ± 1.1 |
| 13 DAG | 202.1 ± 23.3 | 279.4 ± 9.0 | 13.2 ± 3.1 |
| ***Leaves*** | | | |
| 3 DAG | 28.3 ± 4.5 | 153.6 ± 20.8 | 257.8 ± 18.9 |
| 5 DAG | 11.4 ± 1.4 | 78.5 ± 6.7 | 157.3 ± 25.1 |
| 7 DAG | 10.5 ± 0.9 | 163.1 ± 5.3 | 120.3 ± 19.2 |
| 9 DAG | 40.2 ± 5.8 | 310.8 ± 32.1 | 249.6 ± 39.9 |
| 11 DAG | 50.2 ± 4.3 | 293.8 ± 19.8 | 459.8 ± 73.1 |
| 13 DAG | 62.5 ± 3.2 | 286.5 ± 37.7 | 140.8 ± 22.5 |
| ***Roots*** | | | |
| 3 DAG | 71.5 ± 11.5 | 298.2 ± 17.2 | 23.7 ± 3.7 |
| 5 DAG | 118.8 ± 15.6 | 515.6 ± 22.9 | 74.1 ± 11.8 |
| 7 DAG | 135.5 ± 15.5 | 370.3 ± 25.6 | 78.9 ± 12.6 |
| 9 DAG | 138.2 ± 9.0 | 216.1 ± 18.7 | 32.2 ± 1.9 |
| 11 DAG | 365.3 ± 56.6 | 277.6 ± 14.5 | 44.1 ± 2.2 |
| 13 DAG | 144.3 ± 18.9 | 383.7 ± 31.6 | 36.9 ± 7.3 |
| ***Leaves at 9 DAG*** | | | |
| No treatment | 156.2 ± 26.2 | 277.6 ± 27.7 | 23.6 ± 1.1 |
| + 200 mM NaCl | 215 ± 15.1 | 175.9 ± 11.8 | 71.7 ± 5.9 |
| - Nitrogen | 76.7 ± 8.4 | 281.7 ± 24.6 | 68.6 ± 8.2 |
| + 1 µM *t*Z | 109.6 ± 13.2 | 254.3 ± 33.0 | 49.8 ± 5.7 |
| ***Roots at 9 DAG*** | | | |
| No treatment | 120 ± 8.3 | 293.8 ± 23.6 | 32.1 ± 2.7 |
| + 200 mM NaCl | 220.6 ± 15.4 | 227.5 ± 20.8 | 26.4 ± 1.8 |
| - Nitrogen | 46.3 ± 4.7 | 84 ± 9.6 | 28.5 ± 3.1 |
| + 1 µM *t*Z | 89.3 ± 10.7 | 141.6 ± 12.3 | 49.3 ± 5.9 |

**Supplementary Table S6. Transcript abundance of three *ADK* genes in moss.** Abundance is the number of transcripts per ng of total RNA amplified by qPCR. RNA from three technical replicates. Mean values ± standard deviations are shown. N.D., not detected

| | Gene (transcripts $ng^{-1}$ of total RNA) | | |
|---|---|---|---|
| **Sample** | ***PpADK1*** | ***PpADK2*** | ***PpADK3*** |
| Control | 13853 ± 266 | 78 ± 3 | N.D. |
| + 200 mM NaCl | 22342 ± 826 | 120 ± 3 | N.D. |
| - Nitrogen | 14011 ± 579 | 64 ± 6 | N.D. |
| + Mannitol | 30570 ± 832 | 156 ± 10 | N.D. |
| + 10 µM ABA | 18401 ± 516 | 170 ± 10 | N.D. |
| + 10 µM cytokinin (BAP) | 15309 ± 181 | 87 ± 6 | N.D. |
| + 10 µM auxin (2,4-D) | 8482 ± 314 | 61 ± 1 | 3 ± 0 |

**Supplementary Table S7. Nucleoside levels in *A. thaliana ZmADK* overexpressors.** Data were measured after dexamethasone induction at 72 hours. All values are given in pmol $g^{-1}$ FW. Asterisks indicate statistically significant differences in treated lines versus the controls (non-treated plants) in a paired Student's t-test (t-test; *, **, and *** correspond to P-values of 0.05 > p > 0.01, 0.01 > p > 0.001, and p < 0.001, respectively). Levels of IMP, XMP, GMP, DHZRMP, DHZ and DHZROG were below the limit of detection.

| | Metabolite levels (pmol $g^{-1}$ FW) | | | | | |
|---|---|---|---|---|---|---|
| | **WT** | ***ZmADK1 #10-6*** | ***ZmADK1 #11-4*** | ***ZmADK2 #2-4*** | ***ZmADK3 #3-3*** | ***ZmADK3 #11-14*** |
| Ade | 1910.7 ± 306 | 1750.1 ± 145.3 | 1515.4 ± 164.3 | 1427.3 ± 49.8* | 1446.5 ± 78.4 | 1801.0 ± 31.0 |
| Ado | 8.4 ± 1.2 | 5.4 ± 0.5* | 4.6 ± 1.2** | 4.8 ± 1.1** | 3.4 ± 1.0** | 4.7 ± 1.2** |
| Guanosine | 194.4 ± 14.7 | 138.3 ± 7.3** | 155.4 ± 12.7* | 131.6 ± 9.7** | 154.8 ± 16.7* | 154.1 ± 9.7* |
| Xanthosine | 361.5 ± 13.9 | 364.7 ± 10.1 | 238.4 ± 4.9*** | 248.5 ± 16.1*** | 184.9 ± 7.5*** | 221.6 ± 9.9*** |
| Inosine | 52.9 ± 2.5 | 82.4 ± 1.9 | 113.1 ± 6.5** | 133.9 ± 2.3*** | 77.3 ± 6.2 | 51.2 ± 2.3 |
| Cytokinin bases | 0.51 ± 0.06 | 0.50 ± 0.05 | 0.59 ± 0.05 | 0.59 ± 0.03 | 0.49 ± 0.04 | 0.49 ± 0.03 |
| Cytokinin ribosides | 4.33 ± 0.25 | 2.99 ± 0.13** | 2.08 ± 0.27*** | 2.24 ± 0.28*** | 2.16 ± 0.05*** | 2.62 ± 0.10*** |
| Cytokinin monophosphates | 14.08 ± 0.13 | 12.78 ± 0.30** | 13.40 ± 0.33* | 12.40 ± 0.28*** | 12.83 ± 0.65* | 13.89 ± 0.14 |
| iP | 0.32 ± 0.05 | 0.32 ± 0.04 | 0.37 ± 0.06 | 0.36 ± 0.05 | 0.29 ± 0.03 | 0.30 ± 0.03 |
| iPR | 1.24 ± 0.07 | 0.94 ± 0.05** | 0.75 ± 0.16** | 0.74 ± 0.14** | 0.85 ± 0.09** | 0.90 ± 0.14* |
| iPRMP | 7.89 ± 0.32 | 6.67 ± 0.32** | 8.54 ± 0.13* | 7.70 ± 0.23 | 8.05 ± 0.32 | 8.02 ± 0.10 |
| iP7G | 23.81 ± 1.38 | 21.55 ± 0.90 | 25.80 ± 0.06 | 22.14 ± 0.44 | 22.92 ± 0.29 | 20.96 ± 1.10* |
| iP9G | 2.33 ± 0.10 | 2.19 ± 0.07 | 2.62 ± 0.12* | 2.16 ± 0.03 | 2.25 ± 0.07 | 2.17 ± 0.08 |
| *c*Z | 0.15 ± 0.01 | 0.15 ± 0.01 | 0.16 ± 0.01 | 0.17 ± 0.01 | 0.15 ± 0.01 | 0.17 ± 0.01 |
| *c*ZR | 1.78 ± 0.16 | 1.27 ± 0.10** | 0.58 ± 0.07*** | 0.73 ± 0.15** | 0.69 ± 0.18** | 1.06 ± 0.06** |
| *c*ZROG | 1.76 ± 0.14 | 1.75 ± 0.18 | 1.54 ± 0.21 | 1.53 ± 0.31 | 1.45 ± 0.05* | 1.69 ± 0.19 |
| *c*ZRMP | 5.37 ± 0.21 | 5.62 ± 0.31 | 4.35 ± 0.38* | 4.17 ± 0.14** | 4.26 ± 0.31** | 5.34 ± 0.24 |
| *c*Z7G | 29.60 ± 1.20 | 28.88 ± 1.35 | 30.37 ± 0.54 | 25.91 ± 0.74* | 25.43 ± 0.811* | 26.60 ± 1.10* |
| *c*Z9G | 0.60 ± 0.02 | 0.66 ± 0.04 | 0.67 ± 0.02* | 0.58 ± 0.01 | 0.56 ± 0.02 | 0.54 ± 0.02* |
| *t*ZR | 1.20 ± 0.16 | 0.71 ± 0.09** | 0.70 ± 0.04** | 0.72 ± 0.05** | 0.58 ± 0.05** | 0.61 ± 0.04** |
| *t*ZROG | 0.24 ± 0.04 | 0.18 ± 0.02* | 0.17 ± 0.02* | 0.20 ± 0.01 | 0.17 ± 0.01* | 0.18 ± 0.03 |
| *t*Z7G | 11.84 ± 0.43 | 11.19 ± 0.50 | 12.71 ± 0.66 | 10.53 ± 0.31* | 11.12 ± 0.68 | 10.45 ± 0.29* |
| *t*Z9G | 4.27 ± 0.34 | 3.97 ± 0.24 | 4.50 ± 0.28 | 3.69 ± 0.11* | 3.73 ± 0.17 | 3.58 ± 0.07* |
| DHZR | 0.11 ± 0.01 | 0.08 ± 0.01* | 0.05 ± 0.01** | 0.04 ± 0.01*** | 0.03 ± 0.01*** | 0.07 ± 0.02 |
| DHZ7G | 4.08 ± 0.15 | 3.44 ± 0.11** | 3.95 ± 0.27 | 3.37 ± 0.06** | 3.45 ± 0.29* | 2.87 ± 0.13** |
| DHZ9G | 0.29 ± 0.02 | 0.29 ± 0.05 | 0.27 ± 0.04 | 0.25 ± 0.02 | 0.27 ± 0.04 | 0.20 ± 0.02** |

**Supplementary Table S8. Total root area of *pOpOn::ZmADK* transgenic lines in nitrogen-varying conditions.** At least 24 seedlings (biological replicates) per and growth condition were included. Median values, mean and standard error values are shown. Asterisks indicate statistically significant differences in transgenic lines versus the WT controls in a paired Student's t-test (t-test; *, **, and *** correspond to P-values of 0.05 > p > 0.01, 0.01 > p > 0.001, and p < 0.001).

| ***No nitrogen*** | *Stage* | *Median* | *Mean* | *St. error* | *p-value* |
|---|---|---|---|---|---|
| *WT* | 3 DAG | 223.0 | 227.0 | 9.1 | |
| *ZmADK1 #11-4* | 3 DAG | 282.0 | 283.9 | 10.2 | *** |
| *ZmADK3 #3-3* | 3 DAG | 261.0 | 298.3 | 18.8 | *** |
| *ZmADK3 #11-14* | 3 DAG | 263.0 | 258.5 | 9.1 | * |
| *WT* | 6 DAG | 795.5 | 712.6 | 44.4 | |
| *ZmADK1 #11-4* | 6 DAG | 982.0 | 890.6 | 57.9 | ** |
| *ZmADK3 #3-3* | 6 DAG | 1075.0 | 1064.0 | 52.6 | *** |
| *ZmADK3 #11-14* | 6 DAG | 885.0 | 856.7 | 35.9 | * |
| *WT* | 9 DAG | 1907.0 | 1730.4 | 157.0 | |
| *ZmADK1 #11-4* | 9 DAG | 2609.0 | 2315.6 | 205.0 | * |
| *ZmADK3 #3-3* | 9 DAG | 3052.0 | 2773.5 | 139.7 | *** |
| *ZmADK3 #11-14* | 9 DAG | 2270.0 | 2149.9 | 138.4 | * |
| *WT* | 11 DAG | 2917.5 | 2544.7 | 240.7 | |
| *ZmADK1 #11-4* | 11 DAG | 3802.0 | 3290.1 | 263.9 | * |
| *ZmADK3 #3-3* | 11 DAG | 4319.0 | 3915.9 | 198.1 | *** |
| *ZmADK3 #11-14* | 11 DAG | 3249.0 | 3268.5 | 180.3 | * |
| ***Normal nitrogen*** | | | | | |
| *WT* | 3 DAG | 266.5 | 264.1 | 9.1 | |
| *ZmADK1 #11-4* | 3 DAG | 241.0 | 246.1 | 15.5 | - |
| *ZmADK3 #3-3* | 3 DAG | 273.0 | 293.9 | 16.4 | - |
| *ZmADK3 #11-14* | 3 DAG | 277.5 | 281.4 | 10.5 | - |
| *WT* | 6 DAG | 709.5 | 650.3 | 43.7 | |
| *ZmADK1 #11-4* | 6 DAG | 682.0 | 678.8 | 39.4 | - |
| *ZmADK3 #3-3* | 6 DAG | 786.0 | 751.2 | 44.9 | * |
| *ZmADK3 #11-14* | 6 DAG | 768.5 | 714.5 | 38.1 | * |
| *WT* | 9 DAG | 1467.5 | 1504.8 | 131.3 | |
| *ZmADK1 #11-4* | 9 DAG | 1404.5 | 1478.7 | 126.3 | - |
| *ZmADK3 #3-3* | 9 DAG | 1944.0 | 1869.6 | 132.2 | ** |
| *ZmADK3 #11-14* | 9 DAG | 1904.0 | 1787.6 | 139.4 | * |
| *WT* | 11 DAG | 2309.0 | 2165.5 | 228.2 | |
| *ZmADK1 #11-4* | 11 DAG | 2241.0 | 2284.3 | 250.7 | - |
| *ZmADK3 #3-3* | 11 DAG | 2979.0 | 2855.8 | 191.1 | ** |
| *ZmADK3 #11-14* | 11 DAG | 2733.5 | 2579.3 | 177.0 | * |
| ***Double nitrogen*** | | | | | |
| *WT* | 3 DAG | 268.5 | 277.7 | 12.4 | |
| *ZmADK1 #11-4* | 3 DAG | 277.5 | 278.3 | 7.6 | - |
| *ZmADK3 #3-3* | 3 DAG | 267.0 | 266.1 | 11.6 | - |
| *ZmADK3 #11-14* | 3 DAG | 269.0 | 281.7 | 10.4 | - |
| *WT* | 6 DAG | 619.0 | 601.3 | 42.3 | |
| *ZmADK1 #11-4* | 6 DAG | 716.5 | 687.1 | 20.0 | * |
| *ZmADK3 #3-3* | 6 DAG | 667.5 | 655.2 | 32.5 | - |
| *ZmADK3 #11-14* | 6 DAG | 705.5 | 651.7 | 32.9 | - |
| *WT* | 9 DAG | 1308.0 | 1228.5 | 114.2 | |
| *ZmADK1 #11-4* | 9 DAG | 1623.0 | 1561.4 | 64.8 | * |
| *ZmADK3 #3-3* | 9 DAG | 1575.0 | 1534.8 | 97.4 | * |
| *ZmADK3 #11-14* | 9 DAG | 1525.5 | 1388.0 | 106.9 | * |
| *WT* | 11 DAG | 2002.5 | 1911.7 | 165.2 | |
| *ZmADK1 #11-4* | 11 DAG | 2466.0 | 2381.4 | 116.0 | - |
| *ZmADK3 #3-3* | 11 DAG | 2516.5 | 2293.8 | 175.0 | * |
| *ZmADK3 #11-14* | 11 DAG | 2548.5 | 2197.0 | 154.0 | * |

**Supplementary Table S9. Change of leaf area in *pOpOn::ZmADK* transgenic lines in nitrogen-varying conditions.** At least 24 seedlings (biological replicates) per and growth condition were included. Median values, mean and standard error values are shown. Asterisks indicate statistically significant differences in transgenic lines versus the WT controls in a paired Student's t-test (t-test; *, **, and *** correspond to P-values of 0.05 > p > 0.01, 0.01 > p > 0.001, and p < 0.001).

| ***No nitrogen*** | *Stage* | *Median* | *Mean* | *St. error* | *p-value* |
|---|---|---|---|---|---|
| *WT* | 6 DAG | 307 | 330.8 | 26.4 | |
| *ZmADK1 #11-4* | 6 DAG | 348 | 363.3 | 27.8 | - |
| *ZmADK3 #3-3* | 6 DAG | 316 | 302.3 | 33.1 | - |
| *ZmADK3 #11-14* | 6 DAG | 285 | 294.4 | 21.8 | - |
| *WT* | 8 DAG | 480 | 518.5 | 43.5 | |
| *ZmADK1 #11-4* | 8 DAG | 534 | 554.0 | 65.4 | - |
| *ZmADK3 #3-3* | 8 DAG | 531 | 574.5 | 44.7 | - |
| *ZmADK3 #11-14* | 8 DAG | 467 | 468.2 | 29.7 | - |
| *WT* | 10 DAG | 638 | 646.0 | 57.2 | |
| *ZmADK1 #11-4* | 10 DAG | 610 | 627.3 | 87.6 | - |
| *ZmADK3 #3-3* | 10 DAG | 710 | 668.0 | 97.4 | - |
| *ZmADK3 #11-14* | 10 DAG | 600 | 643.5 | 72.4 | - |
| *WT* | 14 DAG | 944 | 915.7 | 82.6 | |
| *ZmADK1 #11-4* | 14 DAG | 970 | 1054.2 | 90.1 | * |
| *ZmADK3 #3-3* | 14 DAG | 916 | 971.3 | 99.6 | - |
| *ZmADK3 #11-14* | 14 DAG | 920 | 914.3 | 50.9 | - |
| ***Normal nitrogen*** | | | | | |
| *WT* | 6 DAG | 3940 | 3910.5 | 240.4 | |
| *ZmADK1 #11-4* | 6 DAG | 6147 | 5644.3 | 336.7 | *** |
| *ZmADK3 #3-3* | 6 DAG | 5544 | 5361.0 | 314.8 | ** |
| *ZmADK3 #11-14* | 6 DAG | 3145 | 3518.0 | 402.4 | - |
| *WT* | 8 DAG | 8480 | 8393.7 | 544.9 | |
| *ZmADK1 #11-4* | 8 DAG | 12239 | 11251.9 | 689.5 | ** |
| *ZmADK3 #3-3* | 8 DAG | 11132 | 11067.7 | 623.5 | ** |
| *ZmADK3 #11-14* | 8 DAG | 8555 | 8253.5 | 814.6 | - |
| *WT* | 10 DAG | 11028 | 11402.0 | 759.5 | |
| *ZmADK1 #11-4* | 10 DAG | 18318 | 17168.5 | 881.4 | *** |
| *ZmADK3 #3-3* | 10 DAG | 15492 | 15389.6 | 894.3 | ** |
| *ZmADK3 #11-14* | 10 DAG | 11586 | 12115.8 | 1450.2 | - |
| *WT* | 14 DAG | 28241 | 27831.4 | 2098.1 | |
| *ZmADK1 #11-4* | 14 DAG | 28703 | 28835.3 | 1668.4 | - |
| *ZmADK3 #3-3* | 14 DAG | 25451 | 27827.6 | 2034.2 | - |
| *ZmADK3 #11-14* | 14 DAG | 25399 | 27175.2 | 3194.5 | - |
| ***Double nitrogen*** | | | | | |
| *WT* | 6 DAG | 3124 | 3315.4 | 259.4 | |
| *ZmADK1 #11-4* | 6 DAG | 4884 | 4544.3 | 315.1 | ** |
| *ZmADK3 #3-3* | 6 DAG | 4140 | 4878.8 | 418.6 | ** |
| *ZmADK3 #11-14* | 6 DAG | 3989 | 3776.8 | 304.7 | - |
| *WT* | 8 DAG | 8247 | 7452.1 | 587.1 | |
| *ZmADK1 #11-4* | 8 DAG | 10156 | 9330.8 | 692.2 | * |
| *ZmADK3 #3-3* | 8 DAG | 9180 | 9520.3 | 775.0 | * |
| *ZmADK3 #11-14* | 8 DAG | 7480 | 6891.2 | 549.9 | - |
| *WT* | 10 DAG | 9982 | 10176.9 | 791.8 | |
| *ZmADK1 #11-4* | 10 DAG | 14209 | 13534.8 | 872.4 | ** |
| *ZmADK3 #3-3* | 10 DAG | 13368 | 12810.3 | 900.7 | * |
| *ZmADK3 #11-14* | 10 DAG | 11332 | 10355.1 | 797.1 | - |
| *WT* | 14 DAG | 15286 | 16580.6 | 1675.2 | |
| *ZmADK1 #11-4* | 14 DAG | 23796 | 23057.8 | 1383.9 | ** |
| *ZmADK2 #2-4* | 14 DAG | 23537 | 23816.0 | 1531.6 | ** |
| *ZmADK3 #3-3* | 14 DAG | 21076 | 20896.9 | 1631.2 | * |
| *ZmADK3 #11-14* | 14 DAG | 19198 | 18943.2 | 1585.0 | - |

**Supplementary Table S10. Levels change among selected amino acids in pOpOn::ZmADK transgenic lines in the absence of nitrogen.** Values are given in nmol $g^{-1}$ DW with standard errors. Data were measured after dexamethasone induction for 48 hours in technical triplicates. Asterisks indicate statistically significant differences in transgenic lines versus the WT controls in a paired Student's t-test (t-test; *, **, and *** correspond to P-values of 0.05 > p > 0.01, 0.01 > p > 0.001, and p < 0.001).

| | **Metabolites (nmol $g^{-1}$ DW)** | | | | | |
|---|---|---|---|---|---|---|
| | Arg | Citrulline | Gln | Glu | Asn | Asp |
| WT | 363.0 ± 53.5 | 107.2 ± 15.6 | 48.2 ± 23.8 | 62.1 ± 8.9 | 151.2 ± 7.1 | 81.1 ± 16.5 |
| *ZmADK1 #10-6* | 470.5 ± 18.1* | 116.5 ± 9.5 | 103.2 ± 3.1* | 72.3 ± 29.1 | 179.5 ± 6.2* | 91.4 ± 12.0 |
| *ZmADK1 #11-4* | 552.4 ± 104.0* | 128.9 ± 18.8 | 121.4 ± 11.8** | 81.5 ± 33.2 | 188.3 ± 2.4** | 102.7 ± 12.1 |
| *ZmADK2 #2-4* | 529.3 ± 13.3** | 125.2 ± 8.7 | 145.9 ± 25.8** | 73.5 ± 28.0 | 161.5 ± 7.3 | 86.8 ± 20.9 |
| *ZmADK3 #3-3* | 516.5 ± 90.2 | 125.3 ± 20.4 | 95.8 ± 14.3* | 86.9 ± 23.0 | 169.1 ± 16.5 | 99.5 ± 10.8 |
| *ZmADK3 #11-14* | 564.9 ± 62.7* | 154.9 ± 20.1* | 99.7 ± 6.0* | 84.7 ± 9.7* | 164.7 ± 21.8 | 101.6 ± 6.0 |

**Supplementary Fig. S1. Thermal stability, molecular and kinetic properties of ZmADK2. (A)** Thermal stability of five plant ADKs measured by nanoDSF. The curves for apoforms (in 150 mM HEPES pH 7.2, 100 mM NaCl, 10 mM $MgCl_2$) and ATP complexes at 4 mM concentrations are shown. **(B)** Gel filtration of highly concentrated ZmADK2 (at 30 mg $ml^{-1}$) on Superdex S200 column. The chromatogram shows the existence of a small peak corresponding to the double molecular weight of the monomer. **(C)** Binding curves of Ado derivatives measured by MST in 50 mM HEPES buffer pH 7.5, 1 mM $MgCl_2$ and 0.1% Tween. **(D)** Saturation curves of Ado derivatives measured in a coupled reaction with PK and LDH at 30°C in 50 mM Tris-HCl buffer pH 7.5 using 10 mM ATP.

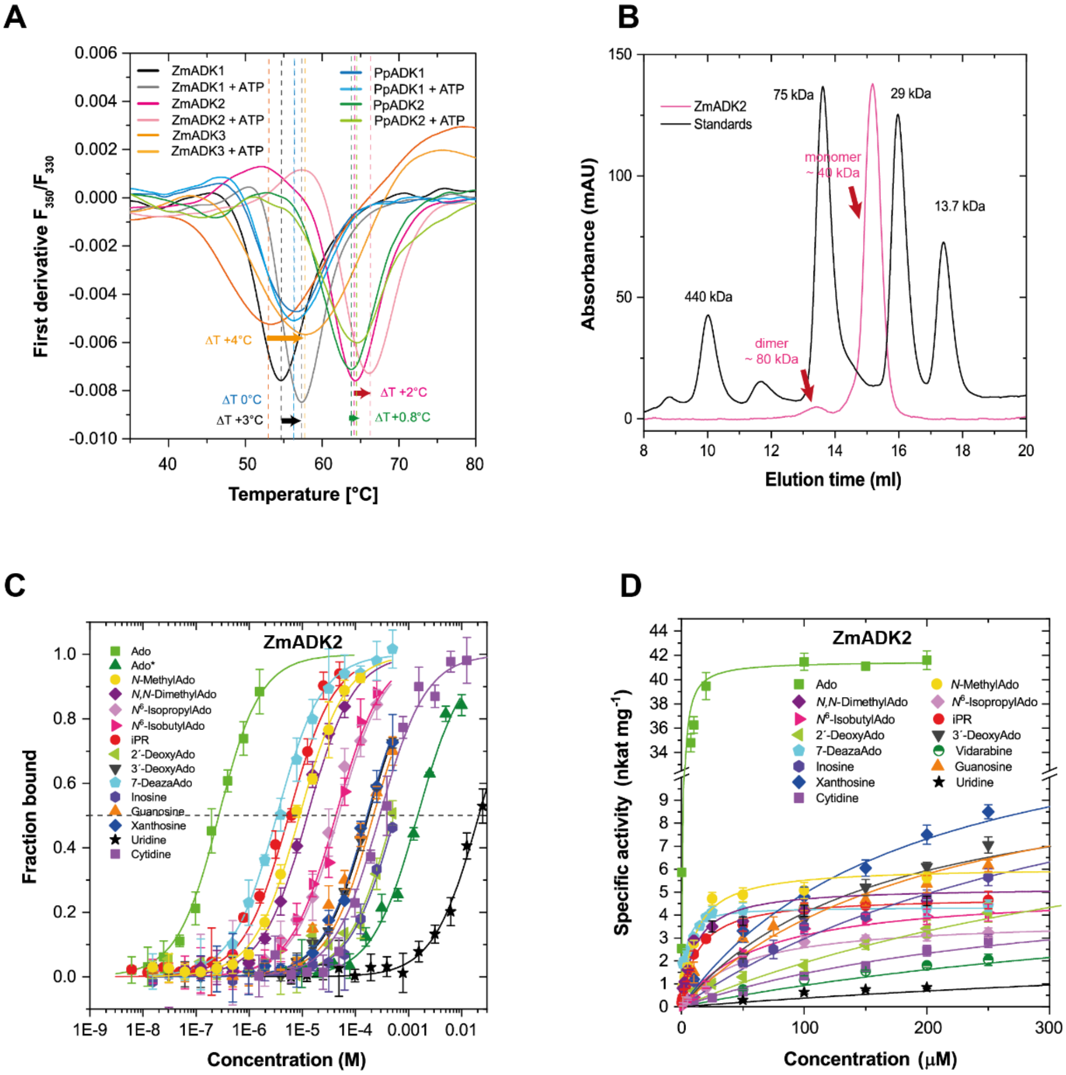

**Supplementary Fig. S2. Reaction scheme for the synthesis of two purine ribosides.** Purine ribosides were prepared in a one-step reaction by heating 6-chloropurine riboside with the corresponding amine in the presence or absence of triethylamine as an auxiliary base. Reagents and conditions: i) methylamine, ethanol, 90 °C, 4 h; ii) dimethylamine hydrochloride, triethylamine, methanol, 100 °C, 4 h; iii) triethylamine, n-propanol, 85 °C, 4 h and isopropylamine (c) or isobutylamine (d).

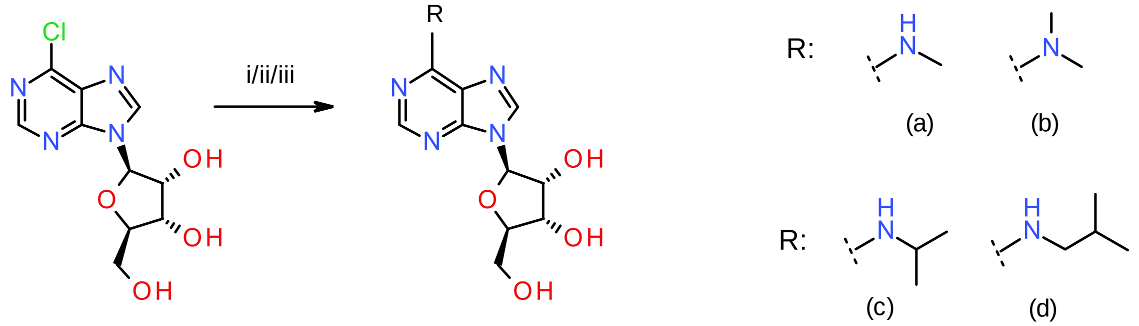


6-chloropurine riboside

*N*6-methyladenosine (a)
*N*6, *N*6-dimethyladenosine (b)
*N*6-isopropyladenosine (c)
*N*6-isobutyladenosine (d)

**Supplementary Fig. S3. NMR spectra of *N*$^6$-methylAdo.** $^1$H-NMR (500 MHz, DMSO-$d_6$) δ (ppm): 2.94 (s, 3H, -NHC<u>H</u>$_3$), 3.54 (ddd, *J* = 12.2, 7.3, 3.7 Hz, 1H, rib H5'), 3.66 (dt, *J* = 12.0, 3.8 Hz, 1H, rib H5'), 3.95 (q, *J* = 3.3 Hz, 1H, rib H4'), 4.13 (td, *J* = 4.6, 3.1 Hz, 1H, rib H3'), 4.59 (q, *J* = 5.9 Hz, 1H, rib H2'), 5.18 (d, *J* = 4.3 Hz, 1H, rib C3'O<u>H</u>), 5.42-5.44 (m, 2H, rib C2' O<u>H</u>, rib C5' O<u>H</u>), 5.87 (d, *J* = 6.7 Hz, 1H, rib H1'), 7.83 (bs, 1H, -N<u>H</u>CH$_3$), 8.22 (s, 1H, pur H2), 8.33 (s, 1H, pur H8). $^{13}$C-NMR (125 MHz, DMSO-$d_6$) δ (ppm): 27.0 (-NH<u>C</u>H$_3$), 61.7 (rib C5'), 70.7 (rib C3'), 73.5 (rib C2'), 85.9 (rib C4'), 87.9 (rib C2'), 119.9 (pur C5), 139.7 (pur C8), 148.0 (pur C4), 152.5 (pur C2), 155.1 (pur C6). HPLC-UV/VIS retention time, purity (min., %): 4.73, 99.9. ESI$^+$-MS *m/z* (rel. int. %, ion): 282.4 (100, [M+H]$^+$). HRMS (ESI/TOF) m/z: [M + H]$^+$ Calcd for $C_{11}H_{16}N_5O_4$ 282.1197; Found 282.1199.

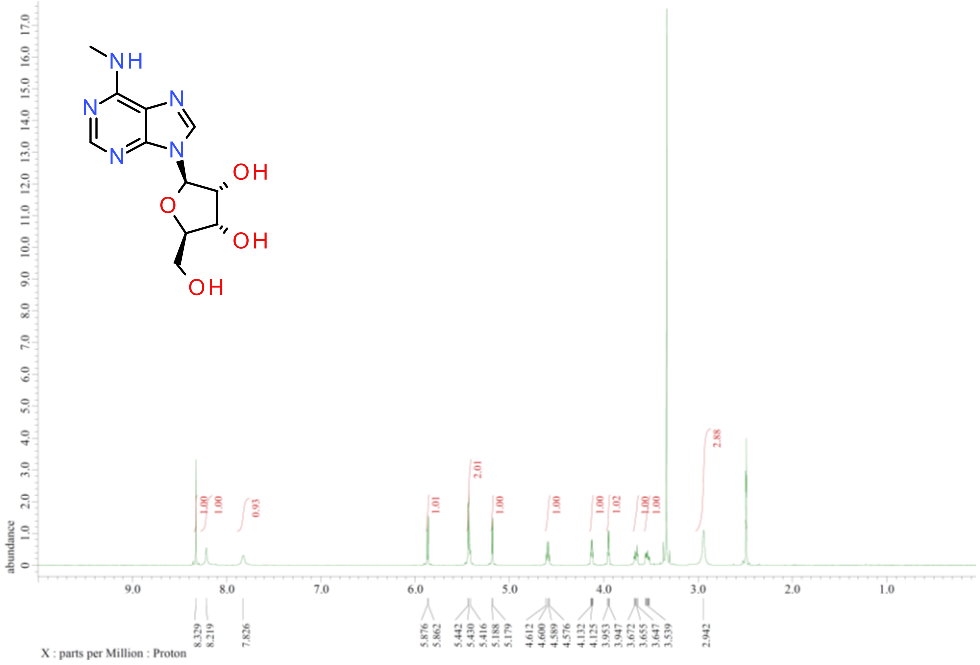


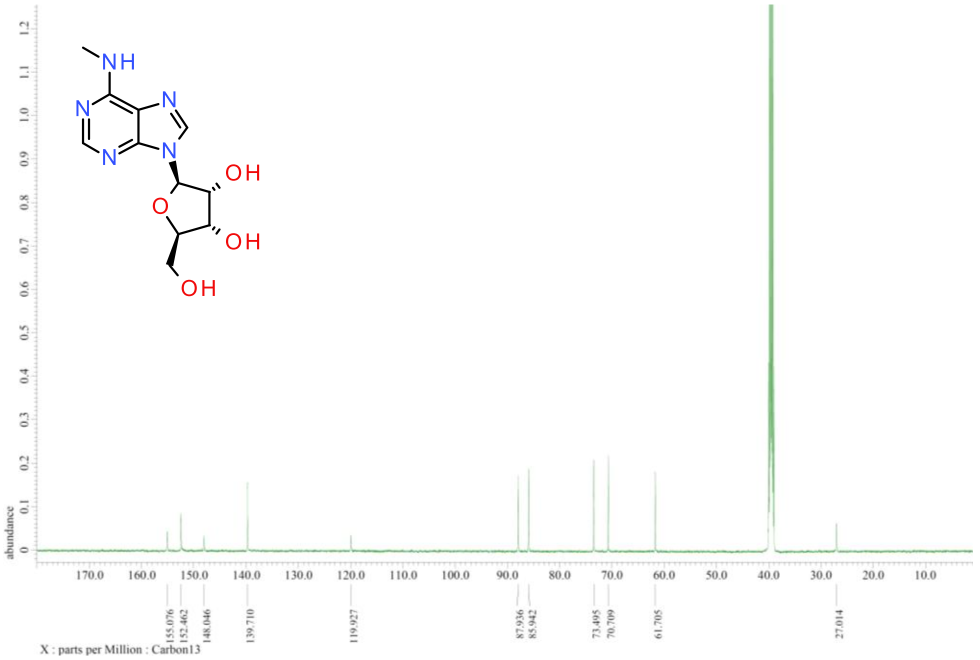

**Supplementary Fig. S4. NMR spectra of $N^6$, $N^6$-dimethylAdo.** $^1$H-NMR (500 MHz, DMSO-$d_6$) δ (ppm): 3.44 (vbs, 6H, -NH(C<u>H</u>$_3$)$_2$), 3.54 (ddd, $J$ = 12.0, 7.0, 3.7 Hz, 1H, rib H5'), 3.66 (dt, $J$ = 12.1, 4.1 Hz, 1H, rib H5'), 3.95 (q, $J$ = 3.4 Hz, 1H, rib H4'), 4.13 (td, $J$ = 4.7, 3.4 Hz, 1H, rib H3'), 4.56 (q, $J$ = 5.7 Hz, 1H, rib H2'), 5.17 (d, $J$ = 4.6 Hz, 1H, rib C3' O<u>H</u>), 5.36 (dd, $J$ = 6.7, 4.6 Hz, 1H, rib C5' O<u>H</u>), 5.43 (d, $J$ = 6.4 Hz, 1H, rib C2' O<u>H</u>), 5.90 (d, $J$ = 6.1 Hz, 1H, rib H1'), 8.20 (s, 1H, pur H2), 8.36 (s, 1H, pur H8). $^{13}$C-NMR (125 MHz, DMSO-$d_6$) δ (ppm): 37.9 (2× C, -N(<u>C</u>H$_3$)$_2$), 61.5 (rib C5'), 70.5 (rib C3'), 73.5 (rib C2'), 85.7 (rib C4'), 87.8 (rib C1'), 119.8 (pur C5), 138.6 (pur C8), 149.9 (pur C4), 151.7 (pur C2), 154.3 (pur C6). HPLC-UV/VIS retention time, purity (min., %): 6.44, 99.9. ESI$^+$-MS $m/z$ (rel. int. %, ion): 296.4 (100, $[M+H]^+$). HRMS (ESI/TOF) m/z: $[M + H]^+$ Calcd for $C_{12}H_{18}N_5O_4$ 296.1353; Found 296.1351.

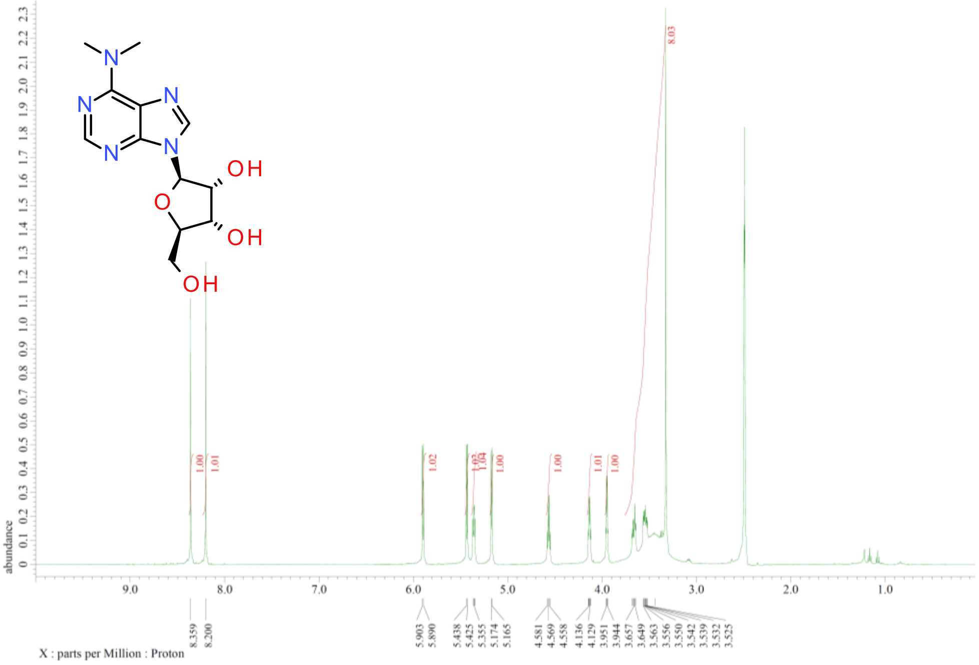


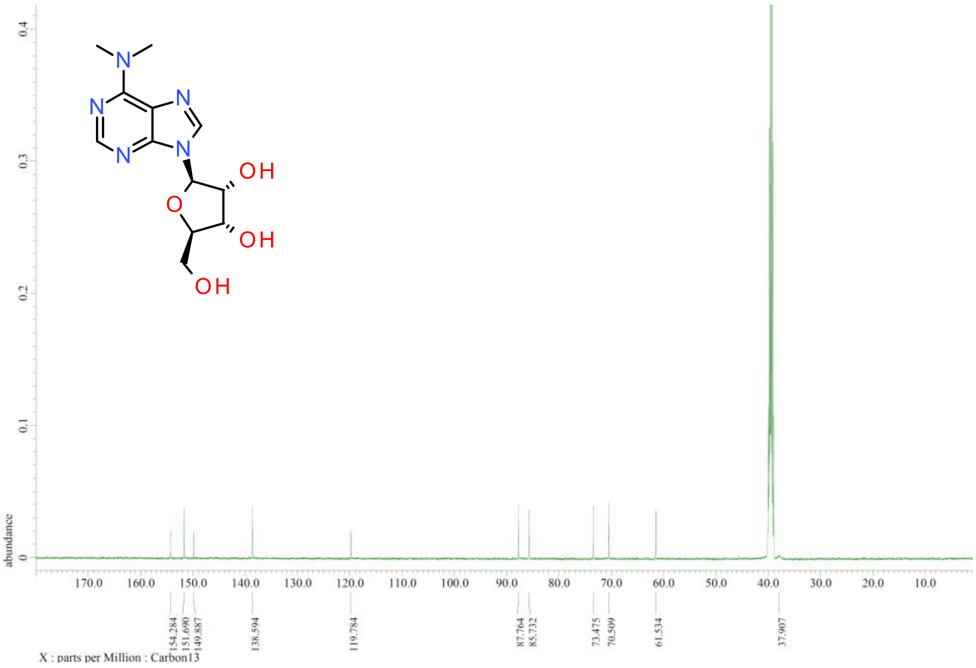

**Supplementary Fig. S5. NMR spectra of *N*$^6$-isopropylAdo.** $^1$H-NMR (500 MHz, DMSO-$d_6$) δ (ppm): 1.20 (d, *J* = 6.7 Hz, 6H, -NHCH(C<u>H</u>$_3$)$_2$), 3.54 (ddd, *J* = 12.1, 7.5, 3.7 Hz, 1H, rib H5'), 3.66 (dt, *J* = 12.1, 4.1 Hz, 1H, rib H5'), 3.95 (q, *J* = 3.3 Hz, 1H, rib H4'), 4.13 (td, *J* = 4.8, 3.0 Hz, 1H, rib H3'), 4.43 (bs, 1H, -NHC<u>H</u>(CH$_3$)$_2$), 4.60 (td, *J* = 6.3, 5.0 Hz, 1H, rib H2'), 5.19 (d, *J* = 4.6 Hz, 1H, rib C3' O<u>H</u>), 5.43-5.46 (m, 2H, rib C2' O<u>H</u>, rib C5' O<u>H</u>), 5.86 (d, *J* = 6.1 Hz, 1H, rib H1'), 7.66 (bd, *J* = 4.9 Hz, 1H, -N<u>H</u>CH(CH$_3$)$_2$), 8.19 (bs, 1H, pur H2), 8.33 (s, 1H, pur H8). $^{13}$C-NMR (125 MHz, DMSO-$d_6$) δ (ppm): 22.3 (2× C, -NHCH(<u>C</u>H$_3$)$_2$), 41.3 (-NH<u>C</u>H(CH$_3$)$_2$), 61.7 (rib C5'), 70.7 (rib C3'), 73.4 (rib C2'), 85.9 (rib C4'), 87.9 (rib C1'), 119.7 (pur C5), 139.6 (pur C8), 148.3 (pur C4), 152.4 (pur C2), 153.9 (pur C6). HPLC-UV/VIS retention time, purity (min., %): 7.41, 97.3. ESI$^+$-MS *m/z* (rel. int. %, ion): 310.3 (100, [M+H]$^+$). HRMS (ESI/TOF) m/z: [M + H]$^+$ Calcd for $C_{13}H_{20}N_5O_4$ 310.1510; Found 310.1525.

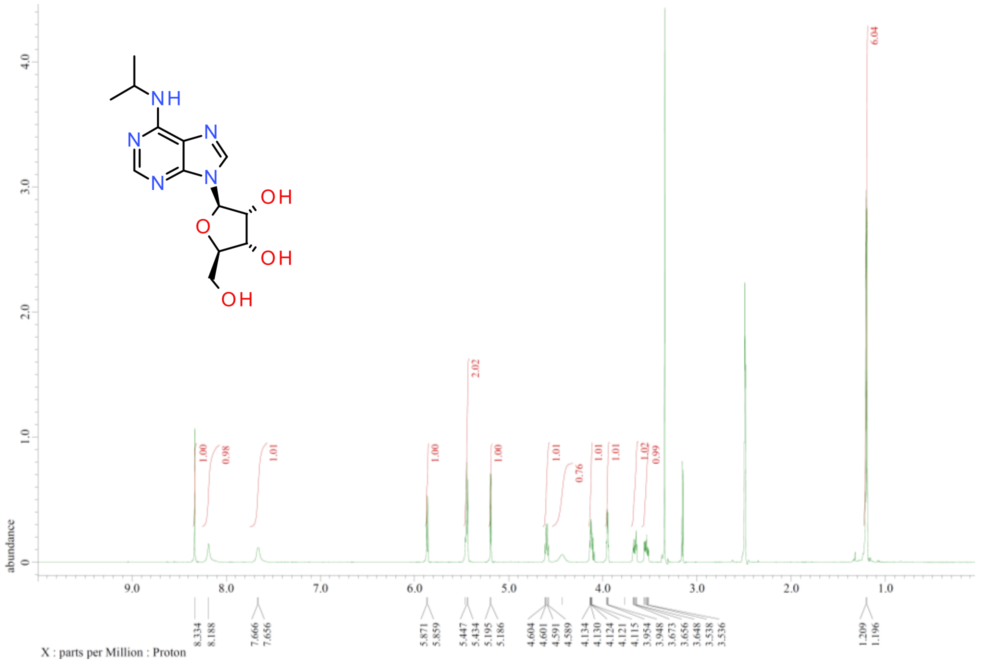


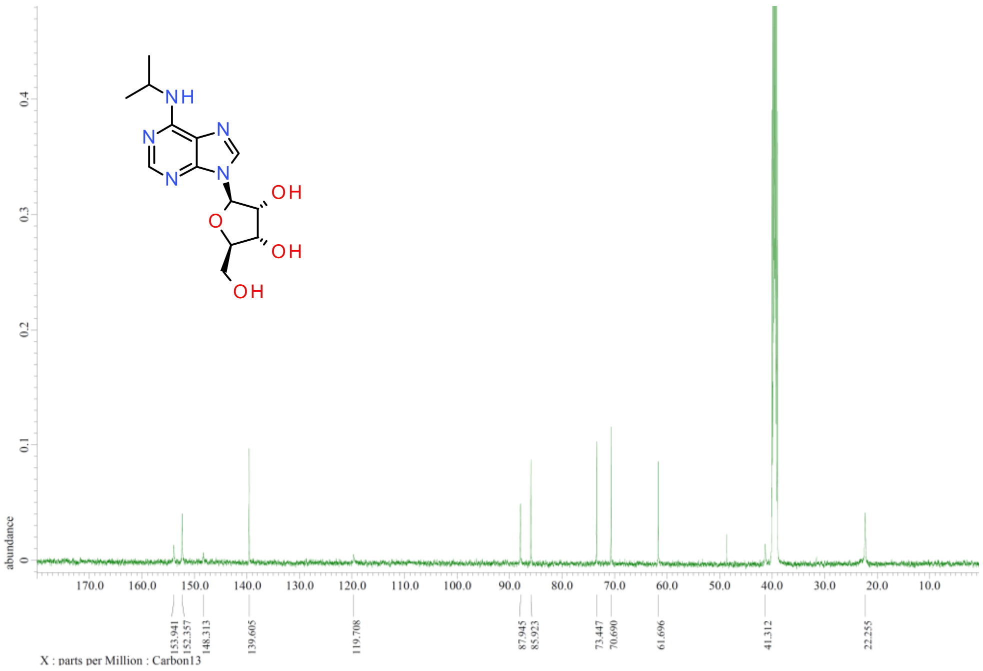

**Supplementary Fig. S6. NMR spectra of *N*$^{6}$-isobutylAdo.** $^{1}$H-NMR (500 MHz, DMSO-$d_6$) δ (ppm): 0.87 (d, $J$ = 6.9 Hz, 6H, $-NHCH_2CH(C\underline{H}_3)_2$), 1.92-1.98 (m, 1H, $-NHCH_2C\underline{H}(CH_3)_2$), 3.28 (bt, $J$ = 5.3 Hz, 2H, $-N\underline{H}CH_2CH(CH_3)_2$), 3.54 (ddd, $J$ = 12.2, 7.4, 3.7 Hz, 1H, rib H5'), 3.66 (dt, $J$ = 12.1, 3.9 Hz, 1H, rib H5'), 3.95 (q, $J$ = 3.2 Hz, 1H, rib H4'), 4.13 (td, $J$ = 4.5, 3.2 Hz, 1H, rib H3'), 4.59-4.62 (m, 1H, rib H2'), 5.18 (d, J = 4.6 Hz, 1H, rib C3' O$\underline{H}$), 5.44 (bd, $J$ = 6.4 Hz, 2H, rib C2' O$\underline{H}$, rib C5' O$\underline{H}$), 5.86 (d, $J$ = 6.4 Hz, 1H, rib H1'), 7.95 (bt, $J$ = 5.3 Hz, 1H, $-N\underline{H}CH_2CH(CH_3)_2$), 8.18 (bs, 1H, pur H2), 8.33 (s, 1H, pur H8). $^{13}$C-NMR (125 MHz, DMSO-$d_6$) δ (ppm): 20.1 (2× C, $-NHCH_2CH(\underline{C}H_3)_2$), 27.8 ($-NHCH_2\underline{C}H(CH_3)_2$), 47.2 ($-NH\underline{C}H_2CH(CH_3)_2$), 61.7 (rib C5'), 70.7 (rib C3'), 73.4 (rib C2'), 86.0 (rib C4'), 88.0 (rib C1'), 119.7 (pur C5), 139.7 (pur C8), 148.2 (pur C4), 152.3 (pur C2), 154.8 (pur C6). HPLC-UV/VIS retention time, purity (min., %): 9.09, 99.9. ESI$^{+}$-MS *m/z* (rel. int. %, ion): 324.4 (100, $[M+H]^{+}$). HRMS (ESI/TOF) m/z: $[M + H]^{+}$ Calcd for $C_{14}H_{22}N_5O_4$ 324.1666; Found 324.1665.

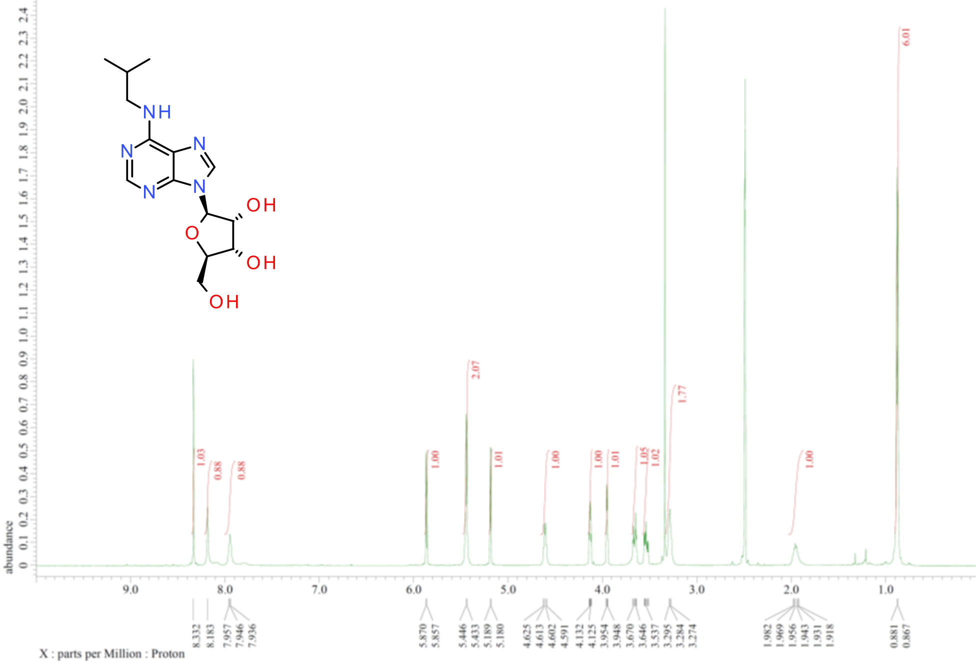


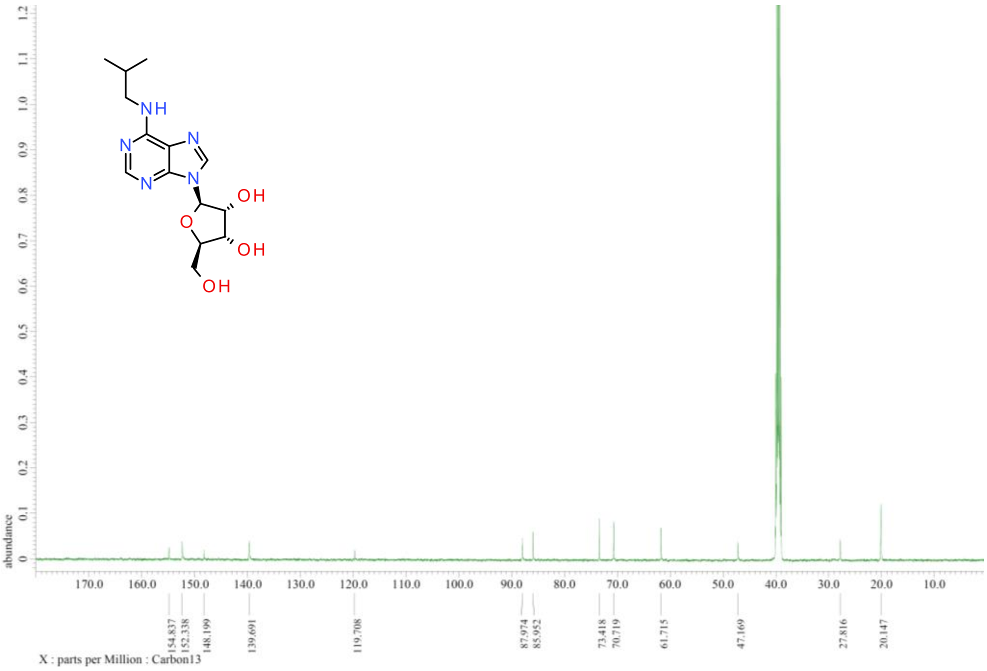

**Supplementary Fig. S7. Sequence alignment of the selected plant ADKs and human ADK.** Sequence accession numbers are as follows: ZmADK1 (Zm00001d051157), ZmADK2 (Zm00001d017271), ZmADK3 (Zm00001d003017), PpADK1 (Pp3c3_10800), PpADK2 (Pp3c13_10550), PpADK3 (Pp3c8_25260), AtADK1 (At3g09820), AtADK2 (At5g03300) and human ADK (Uniprot accession: P55263). The motif found in ADK sequences is shown in a red rectangle, while residues forming the dimer interface are highlighted in green.

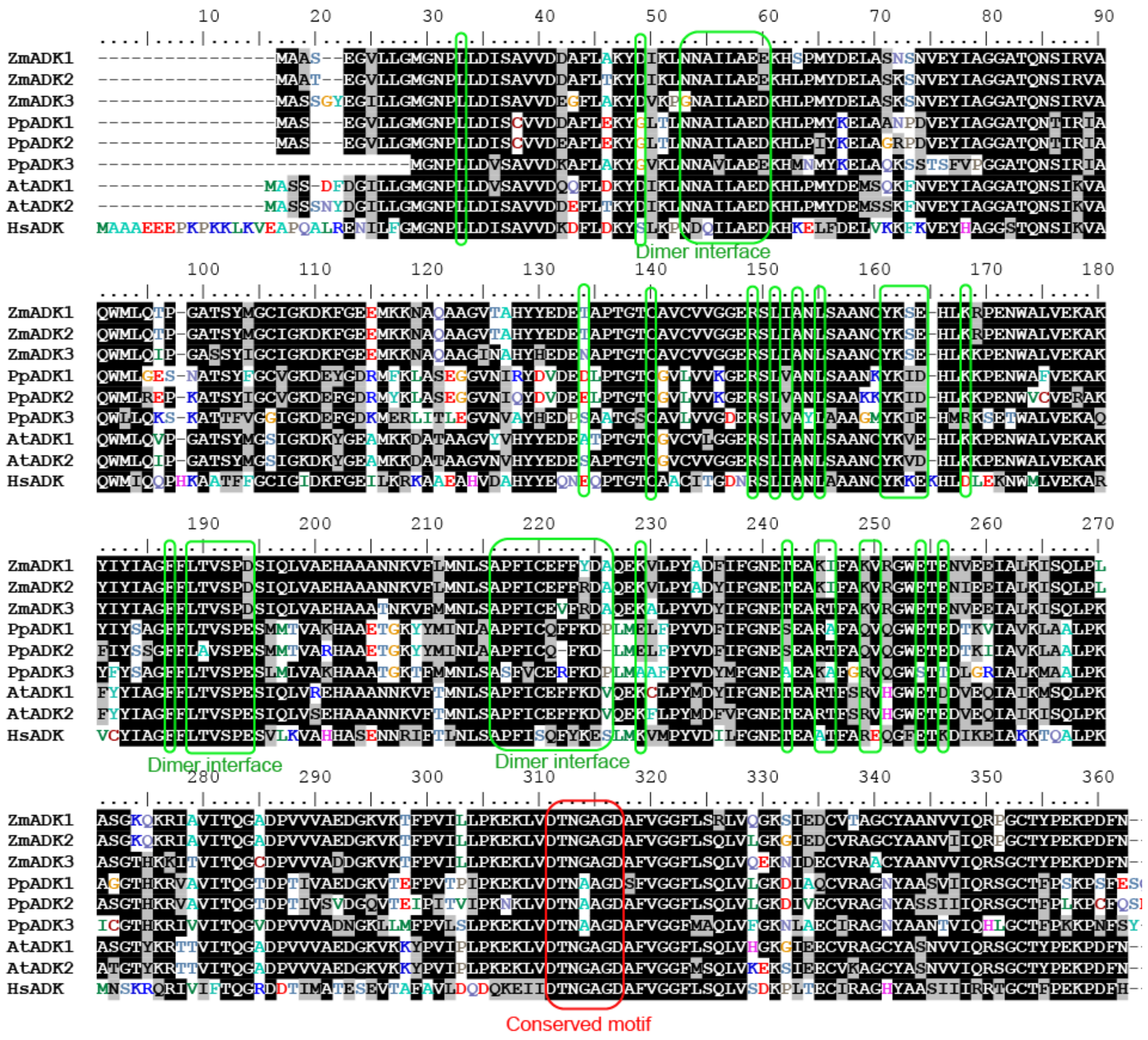

**Supplementary Fig. S8.** ***In silico docking*** **of cytokinin riboside in the active site of plant ADK. (A)** The active site of ZmADK3 (PDB 8RPA) with docked molecules of iPR (green) and Ado (yellow), neighboring residues are labeled, those that were flexible are colored in orange. **(B)** The active site of PpADK1 (PDB 9FW6) with docked molecules of iPR (green) and Ado (yellow). **(C)** The active site of human ADK (PDB 1BX4) with docked molecules of iPR (green) and Ado (yellow), neighboring residues are labeled.

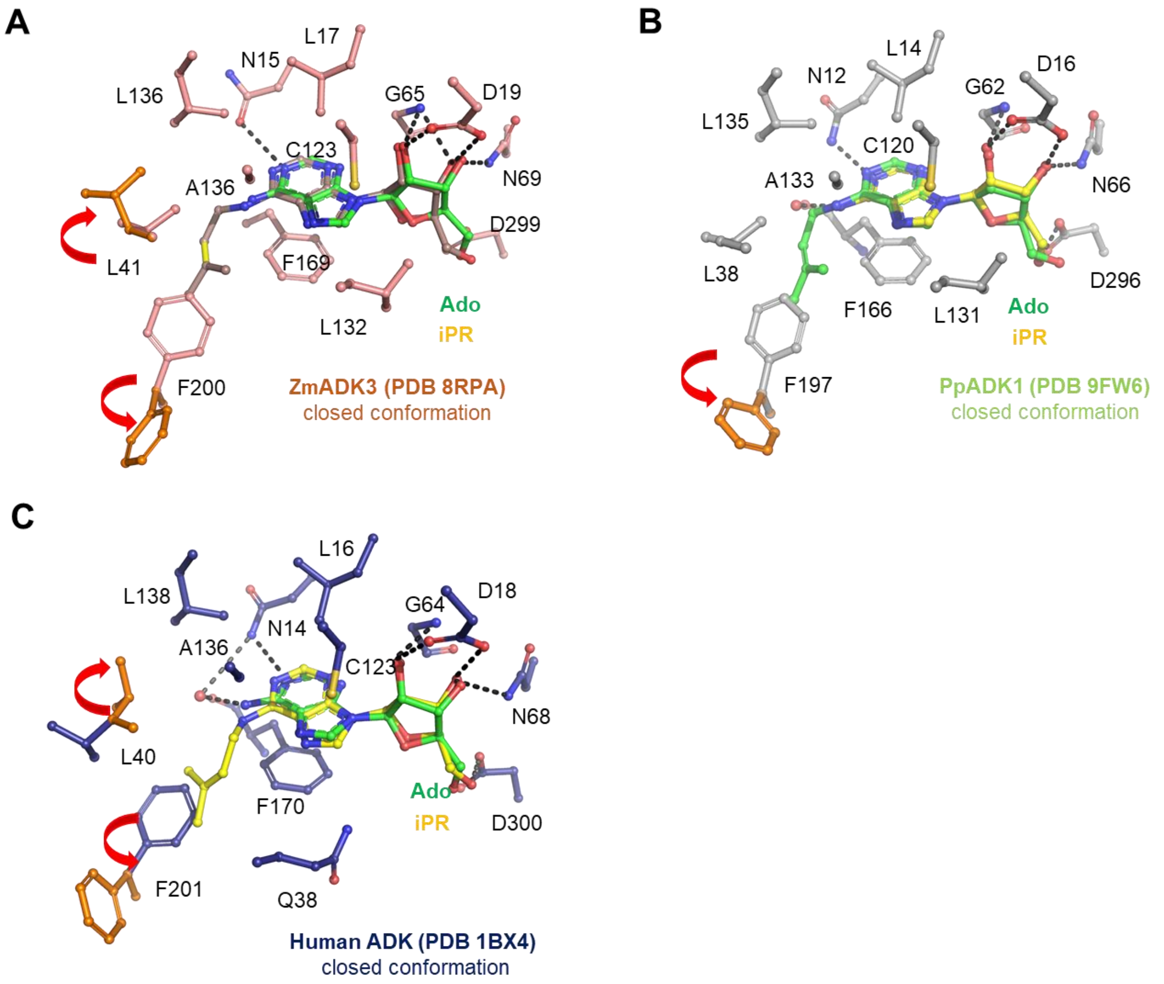

**Supplementary Fig. S9. Comparison between the two known ADK dimers.** The figure shows **(A)** the surface representation of ADK dimer from *Mycobacterium tuberculosis* in the closed conformation (PDB 2PKK, 22% sequence identity with ZmADKs), **(B)** ADK dimer from *Mycobacterium tuberculosis* in the open conformation (PDB 2PKN) **(C)** plant ADK2 dimer from *Zea mays* in the open conformation (PDB 8RF7, this work).

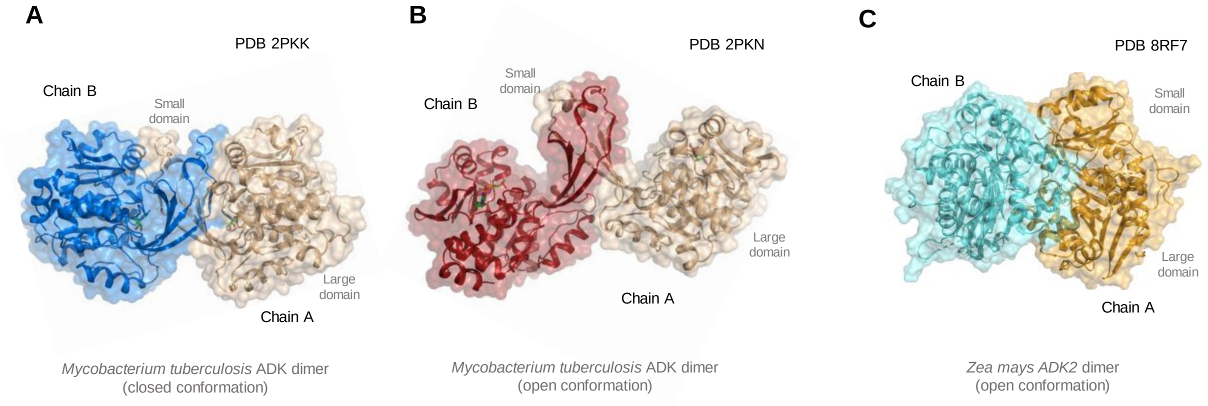

**Supplementary Fig. S10. Oligomeric state of ADK2 from *Arabidopsis thaliana*. (A)** Nu-PAGE of 6His-AtADK2 purified by NiNTA. **(B)** Gel permeation chromatography profile of 6His-AtADK2 (1.2 mg ml$^{-1}$) on a Superdex 200 10/30 HR column in 20 mM Tris-HCl pH 7.5, 100 mM NaCl, with calibration performed using a gel filtration standard (Bio-Rad). **(C)** The hydrodynamic diameter of AtADK2 determined by DLS, measured in 20 mM Tris-HCl pH 7.5 and 1 mM $MgCl_2$ at 22°C at two concentrations (0.5 and 5.0 mg ml$^{-1}$). **(D)** An AlphaFold3 prediction model of the AtSnRK1-AtADK2 (alpha subunit) heterodimer. The model was constructed using the sequences AT3G29160 and AT5G03300 along with one ATP and two AMP molecules.

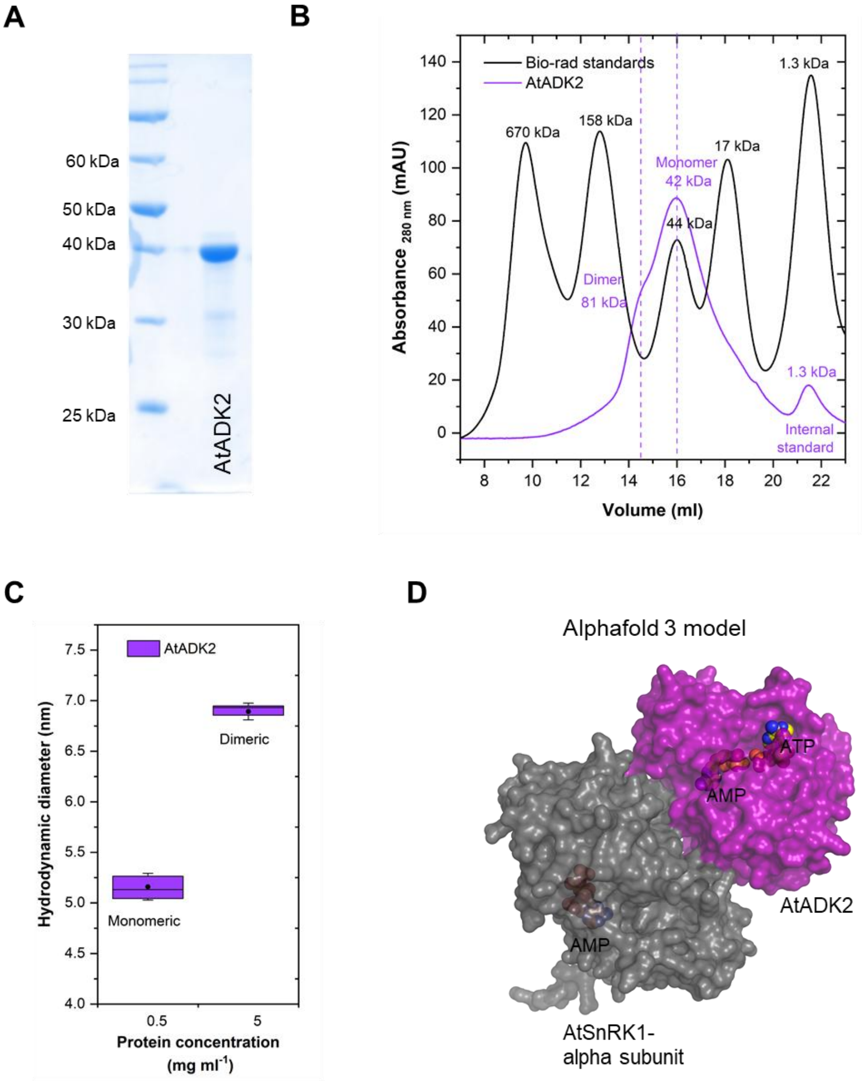

Supplement: eraf094_suppl_Supplementary_Tables_S1-S9_Figures_S1-S10 [file eraf094_suppl_supplementary_tables_s1-s9_figures_s1-s10.pdf]
